# Mapping European Welfare Models: State of the Art of Strategies for Professional Integration and Reintegration of Persons with Chronic Diseases

**DOI:** 10.3390/ijerph15040781

**Published:** 2018-04-17

**Authors:** Chiara Scaratti, Matilde Leonardi, Fabiola Silvaggi, Carolina C. Ávila, Amalia Muñoz-Murillo, Panayiota Stavroussi, Olga Roka, Helena Burger, Klemens Fheodoroff, Beata Tobiasz-Adamczyk, Carla Sabariego, Eva Esteban, Sonja Gruber, Olga Svestkova, Rune Halvorsen, Asel Kadyrbaeva, Sabrina Ferraina

**Affiliations:** 1 Neurology, Public Health and Disability Unit, Neurological Institute C. Besta IRCCS Foundation, 20133 Milan, Italy; matilde.leonardi@istituto-besta.it (M.L.); fabiola.silvaggi@istituto-besta.it (F.S.); 2 Department of Psychiatry, Universidad Autónoma de Madrid and CIBER of Mental Health (CIBERSAM), 28028 Madrid, Spain; carolina_avila@hotmail.com; 3 Innovation and Teaching Unit, Institut de Recerca Sant Joan de Déu, Esplugues de Llobregat, 08830 Barcelona, Spain; a.munoz@pssjd.org; 4 Parc Sanitari Sant Joan de Déu, Universitat de Barcelona, Fundació Sant Joan de Déu, Sant Boi de Llobregat, 08830 Barcelona, Spain; 5 Department of Special Education, University of Thessaly, 38221 Volos, Greece; stavrusi@uth.gr (P.S.); olgaroc1@yahoo.gr (O.R.); 6 The University Rehabilitation Institute, Republic of Slovenia, 1000 Ljubljana, Slovenia; helena.burger@ir-rs.si; 7 Gailtal Klinik—Neurologische Rehabilitation, 9620 Hermagor, Austria; klemens.fheodoroff@gailtal-klinik.at; 8 Department of Epidemiology and Preventive Medicine, Jagiellonian University Medical College, 31-008 Krakow, Poland; mytobias@cyf-kr.edu.pl; 9 Chair for Public Health and Health Services Research, Research Unit for Biopsychosocial Health, Department of Medical Information Processing, Biometry and Epidemiology (IBE), Ludwig-Maximilians-Universität (LMU), 81377 Munich, Germany; carla.sabariego@med.lmu.de (C.S.); eva.esteban@med.lmu.de (E.E.); 10 Disability and Diversity Studies, Carinthia University of Applied Science (CUAS), 9020 Klagenfurt, Austria; s.gruber@fh-kaernten.at; 11 Department of Rehabilitation Medicine 1th Medical Faculty Charles University and University Hospital in Prague, 12800 Praha, Czech Republic; olga.svestkova@lf1.cuni.cz; 12 Department of Social Work, Child Welfare and Social Policy, Oslo and Akershus University College of Applied Sciences, 0130 Oslo, Norway; rune.halvorsen@hioa.no; 13 European Association of Service providers for Persons with Disabilities (EASPD), 1040 Brussels, Belgium; asel.kadyrbaeva@easpd.eu (A.K.); sabrina.ferraina@easpd.eu (S.F.)

**Keywords:** chronic diseases, employment, professional (re)integration, welfare models, strategies, policies, systems, services

## Abstract

*Background:* Persons with chronic diseases (PwCDs) often experience work-related problems, and innovative actions to improve their participation in the labor market are needed. In the frame of the European (EU) Pathways Project, the aim of the study is to compare existing strategies (policies, systems, and services) for professional (re-)integration of PwCDs and mental health conditions available at both European and national level between different European welfare models: Scandinavian, Continental, Anglo-Saxon, Mediterranean, and “Post-Communist”. *Method*: The European strategies were identified by an overview of relevant academic and grey literature searched through Medline and internet searches, while national strategies were explored through questionnaires and in-depth interviews with national relevant stakeholders. *Results*: The mapping of existing strategies revealed that, both at European and national level, PwCDs are often considered as part of the group of “persons with disabilities” and only in this case they can receive employment support. European countries put in place actions to support greater labor market participation, but these differ from country to country. *Conclusion*: Strategies targeting “persons with disabilities” do not necessarily address all the needs of persons with chronic diseases. Countries should consider the importance of employment for all to achieve smart, sustainable, and inclusive growth.

## 1. Introduction

Chronic diseases, or non-communicable diseases (NCDs), are broadly defined by the World Health Organization (WHO) as diseases of long duration and generally slow progression and are the result of a combination of genetic, physiological, environmental and behaviors factors [1]. Based on this definition, that provides the more holistic framework to approach the complexities involved in the relation of chronic diseases and employment, the terms “non-communicable diseases” and “chronic diseases” are interchangeably used in this manuscript. 

Over one-third of the European population aged 15 years or older lives with a chronic disease and 23.5% of the working population in the EU suffer from a chronic illness, while two out of three people at retirement age have at least two chronic conditions [2]. Evidence shows that chronic diseases have a significant impact on labor supply in terms of workforce participation, hours worked, job turnover and early retirement [3]. For individuals with chronic conditions, those diseases also mean barriers to employment and stigma, with consequences on wages, earnings, and positions reached/level of seniority in an organization [4]. Moreover, it has been extensively observed that chronically ill employees have reduced employment prospects, as many of them experience difficulties either staying at work or returning to work after a period of absence [5]. Persons with longstanding health problems, in fact, face higher rates of unemployment and inactivity [5]. Based on the data of the 2011 ad hoc module of the EU Labor Force Survey [6], the employment rate in EU-28 for persons with limitations in work caused by a health condition (38.1%) was 29.6 percentage points less than for people with no such limitations (67.7%). According to a recent systematic review [7] a poor health state and presence of a chronic disease are important predictors of exit out of paid job due to entering the disability pension, unemployment, or early retirement schemes. A poor health and a chronic disease can negatively influence the likelihood of entering paid jobs among unemployed people [8].

However, work is a protective factor for PwCDs. Carlier et al. demonstrated that those who re-entered paid work were three times more likely to change from poor to good health and twice more likely to change from poor to good quality of life than those who continued to be unemployed [9]. Other studies confirmed that entering paid employment had a positive effect on physical and mental health [10,11]. Moreover, encouraging the rapid return to work for PwCDs is a fundamental objective for the economy in every work context [3]. Therefore, allocation of resources for professional integration or re-integration of PwCDs can be considered as investment.

Even if the relationship between poor health and unemployment is consistent across Europe, previous studies have highlighted that it seems to vary across the type of welfare state regime [7,8,12]. The consequences of poor health on employment status, in fact, also depend on social and labor market circumstances, e.g., the level of protection for workers with chronic diseases against workforce exclusion, the rehabilitation policies, the inclusion of people with poor health in regular or sheltered employment.

It is important to consider that, following the definition of disability of the Convention on the Rights of Persons with Disabilities (PwD) [13] and due to the burden they experience in daily life, many PwCDs can be as well considered PwD. This fact has been corroborated by diverse studies [14,15], and becomes also clear if we look at the Global Burden of Disease Study [16,17], which overwhelmingly identified NCDs, many times chronic conditions, as the ones mostly associated to disability. In fact, most people who receive disability benefits in Europe have chronic diseases.

Considering the above, the objective of this study is to compare existing strategies for professional integration and reintegration of persons with chronic diseases, including mental health conditions, available at both European and national level between different European regions, considering cultural and social differences. This comparison of existing strategies provides relevant stakeholders, especially policy makers, with an overview including a set of useful practices that could be transferred between countries or used across European countries.

## 2. Materials and Methods 

This study was carried out in the frame of the EU-funded project PATHWAYS (PArticipation To Healthy Workplaces And Inclusive Strategies in the work sector), a 3-year project that involves 12 partners from 10 different European countries, namely Austria (AT), Belgium (BE), Czech Republic (CZ), Germany (DE), Greece (EL), Italy (IT), Norway (NO), Poland (PL), Slovenia (SI) and Spain (ES), with the aim to develop innovative approaches to promote the professional integration and reintegration of people with chronic diseases and improve their employability (www.path-ways.eu).

For the purposes of this study, persons with chronic diseases in general and persons with disability in general were considered; persons with disability were included as usually most people who receive disability benefits have chronic diseases and experience significant levels of disability in daily life [18]. Moreover, the disease groups that constitute mental disorders, musculoskeletal disorders, cancer, neurological, metabolic, and respiratory and cardiovascular diseases were selected based on their impact on labor market participation and on their contribution to years lost due to disability (YLD) in Europe [19].

The study analyzed existing strategies that are currently operating at EU and at national level in ten countries: the nine countries from which partners of the consortium belong to, with the exclusion of Belgium (because the partner is an European Association that does not operate at National level) and the inclusion of United Kingdom (UK), in order to represent the five European welfare models:

- Anglo-Saxon model: United Kingdom
- Scandinavian model: Norway
- Continental model: Austria, Germany, Slovenia
- Mediterranean model: Greece, Italy, Spain
- Post-Communist model: Czech Republic, Poland.

Table 1 reports the main features of the five European welfare models analyzed.

Strategies considered in this study included the levels of policies, systems, and services. Policies are binding and non-binding legislative frameworks, provisions and approaches that set a course or a principle of action at local, regional, national, or international level (e.g., anti-discrimination law). System strategies include supports, programs, or schemes (including financial support) aimed at promoting employment. Services strategies encompass activities by private or public entities aimed at assisting jobseekers in finding employment as well as social services that directly or indirectly contribute to the employability of persons with chronic diseases.

### 2.1. European Level Strategies

The European policies, systems and services were identified by an overview of relevant academic and grey literature searched through Medline and internet searches on the web from May 2015 until April 2016. Data from sources such as Eurostat, European Statistics of income and Living conditions, the Academic Network of European disability experts (ANED), The Organization for Economic Co-operation and Development (OECD) and European Commission reports were included. The internet searches were done in English for materials published within the past ten years (since 2005). Webpages of relevant European and International organizations were also consulted. Examples of search terms were “chronic diseases” (in general) and specific disease groups: “mental disorders”, “musculoskeletal disorders”, “cancer”, “neurological”, “metabolic”, “respiratory” and “cardiovascular diseases”; employment, integration, reintegration, return to work; job maintenance. To have a more comprehensive overview of European policies on the inclusion of persons with ill-health in the labor market, it was decided to consider a wide range of policies areas, including policies on the rights of persons with disabilities, inclusion and anti-discrimination, and employment. Although these policies do not necessarily specifically address chronic illnesses, they do provide overarching frameworks that may promote work (re-)integration policies for persons with chronic diseases. The study takes a closer look at frameworks shaped by European institutions with an objective to improve the employment of persons with chronic diseases. Policies supporting employment (re-)integration of persons with chronic diseases both directly (e.g., specifically targeted at PwCDs) and indirectly (i.e., PwCDs as parts of broader categories, disability or other) were considered.

It should be noted that this study considers only those policies, measures and services that deal with employment. Thus, the study does not consider policies focusing solely on the health aspects of NCD prevention and control.

### 2.2. National Level Strategies

National strategies were collected in the ten countries mentioned above (Austria, Czech Republic, Germany, Greece, Italy, Norway, Poland, Slovenia, Spain, and United Kingdom) through a multi-step approach. The first step was the distribution of questionnaires to national experts, in local languages, carried out by the partners of the Pathways project. The questionnaire included questions about national-level legislation regulating the employment of persons with reduced work capacity, disease/disability-specific legislation, schemes, and services (questionnaire is available on request). In each country, 10 questionnaires were distributed among the national experts, selected by each partner, in different fields related to employment and chronic diseases (both in general and specific chronic diseases), to ensure the coverage of all 7 chronic disease categories selected. The second step was in-depth interviews conducted by all partners of the Consortium with representatives of three main categories of key stakeholders: Users (persons with chronic diseases or advocacy groups), Professionals (healthcare or social care professionals, including medical practitioners) and Authorities (national, regional, local governments or policy makers). Interviews focused on the same areas of the questionnaire but allowed more in-depth exploration of the national situation and the filling in of possible gaps derived from the questionnaires. Whenever indicated or suggested from the expert stakeholders interviewed, we searched for also national grey literature in the local language to integrate the information collected. Convenience sampling was used as the main sampling procedure both for questionnaires and interviews; specifically, stakeholders from national organizations with expertise on employment and health issues or specialized on the different diseases (with focus on patients’ associations/self-help groups) were invited to complete the questionnaire and to conduct in-depth interviews. It was decided to involve stakeholders dealing with chronic diseases in general, or/and with the above-mentioned disease groups: mental disorders, musculoskeletal disorders, cancer, neurological, metabolic, and respiratory and cardiovascular diseases; moreover, also stakeholders dealing with employment and disability in general were included. Stakeholders invited included: policy makers, experts/professionals in the field of employment re-integration of PwCDs, employers in the private sector, and representatives of patients’ associations - located in the ten European countries of the project Consortium. The third step was a validation of the final findings of the study, emerged from the analysis of questionnaires and interviews, through two focus groups in April 2016 involving the partners. Project partners provided their expert knowledge at national level to fill in the gaps in identified strategies (prior to the focus group, they were asked to conduct an additional research on internet and ask follow-up questions to respondents). They were also asked to provide feedback on the identified strategies and engaged in the discussion on the final classification of the strategies.

The available strategies captured through questionnaires and interviews were classified into policies, systems, and services. Based on these categories, they were compared across different welfare models. The mapping of strategies for professional (re-)integration of persons with NCDs in the ten selected countries has been carried out following the structure outlined below:

Policies:

- Availability of legislative frameworks on chronic diseases, mental health, and employment;
- Availability of legislative frameworks on disability and employment;
- Policy provisions on mainstream and specialist employment programs;
- Policy provisions on access to employment support;
- Policy provisions promoting persons-centered approach and individualized service provision;
- Policy provisions on localized and accessible employment service provision;

Systems:

- Employment support in the open labor market;
- Employment support through social enterprises or social cooperatives;
- Employment support through sheltered work;
- Incentives for persons with NCDs to participate in activation programs;
- Financial incentives for employers to recruit/retain persons with NCDs;
- Non-financial incentives for employers to recruit/retain persons with NCDs;
- Duties of persons with NCDs to participate in activation programs;
- Duties of employers (e.g., quota systems);

Services:

- Availability of general and specialized employment services for persons with NCDs.

## 3. Results

### 3.1. European Level Strategies

The detailed comparative results on European level strategies found are shown in Table 2, considering both policies supporting employment (re-)integration of persons with chronic diseases directly and indirectly. In general, the overview of the European strategies shows that the focus is almost exclusively on policies that concern the high level of European framework definition. The search has revealed that to a large extent the employment activation of persons with NCDs is targeted through:

- Policy frameworks on the employment of persons with disabilities (e.g., EU Directive on Employment Equality 2000/78/EC, European Disability Strategy 2010–2020);
- Policy frameworks on employment activation and inclusion in the labor market (e.g., Europe 2020: the European Union strategy for growth and employment, Council Recommendation on the integration of the long-term unemployed into the labor market, Commission Recommendation 2008/867/EC on the active inclusion of people excluded from the labor market).

Policy provisions specifically focusing on the professional (re-)integration of PwCDs are often part of broader policy frameworks. For example, the EU Strategic Framework on Health and Safety at Work 2014–2020 specifically mentions supports in recruitment and return to work of people with a chronic or rare disease, disability or mental conditions, and the use of integrated employment measures such as individualized support, counselling, guidance, access to general and vocational education and training, and other. 

There is also a number of policy reports and actions specifically targeted at chronic diseases or at particular chronic conditions (e.g., Reflection Process on Chronic diseases: Final Report, Joint Action on Chronic Diseases (JA-CHRODIS), Green Paper on Improving the mental health of the population, Joint Action Mental health and Well-being, CANCON Joint Action 2014–2017).

The detailed descriptions of all the European strategies found, is available on the website of the project (www.path-ways.eu) and as Supplementary Material to the manuscript.

### 3.2. National Strategies

In total, 84 questionnaires and 31 interviews were carried out for the following countries: United Kingdom (Anglo-Saxon model); Norway (Scandinavian model); Austria, Germany, and Slovenia (Continental model); Greece, Italy, and Spain (Mediterranean model); Czech Republic and Poland (Post-Communist model). The complete lists of respondents per country are included in the appendix (Questionnaire: Appendix A (Table A1), and interviews: Appendix B (Table A2)).

Table 3 reports the detailed results about national policies and systems, emerged from the analyses of questionnaires and interviews.

#### 3.2.1. National Policies

##### Legislative Frameworks specifically on Chronic Diseases and Employment 

According to the responses to questionnaires and interviews, in all the countries reviewed in this study, there are no legal frameworks specifically covering the employment integration of PwCDs. In most cases persons with NCDs are considered as part of a group of persons with disabilities and reduced work capacity. Therefore, they might be covered by legislation for persons with disabilities to some extent—depending on type of chronic disease and type of national classification system.

##### Legislative Frameworks on Mental Health and Employment 

In the case of mental health conditions, more specific frameworks are available. For instance, Norway has in place a National Strategic plan on Work and Mental Health, and in the UK, a national strategy, “Five Year Forward View for Mental Health”, for the National Health Service in England has been published. 

##### Legislative Frameworks on Disability and Employment

Legislative frameworks on disability in all countries provide a solid foundation for fighting against discrimination in employment and employment services. All countries have ratified the United Nations Convention of Rights of Persons with Disabilities (UNCRPD) and at least at policy level it provides the framework to recognize the rights of persons with disabilities to equal work opportunities. Depending on the definition of disability in different countries, persons with chronic health conditions can be recognized as “disabled” and be protected from unfair treatments and inequalities or benefit from additional supports.

##### Policy Provisions on Mainstream and Specialist Employment Programs

In all countries Public Employment Services aim to provide services to persons with reduced work capacity and have units or personnel that can refer jobseekers with specific needs to specialized services.

##### Policy Provisions Allowing Access to Employment Rehabilitation Support for Persons with NCD, without Making Disability a Prerequisite

All countries provide support to persons with disabilities or persons from vulnerable social groups in finding, getting, and staying in employment. However, in most countries this support is not automatically available for persons with chronic diseases. In all countries from the Mediterranean welfare model, disability is a prerequisite for additional support in job seeking. In Greece, for example, persons with NCDs with a disability level below 50% cannot access employment rehabilitation programs, regardless if they still have support needs. A similar situation exists in Austria, but the assessment of disability varies in both countries. In some cases, as in Poland, people have no chance to work at all if they are found “incapable of doing any gainful work”. In Spain, persons with chronic diseases, however, still have a possibility to access assistance in job adaptation. In the UK and Norway, persons with health conditions are included in provisions for employment support. For example, in the UK, persons with cancer would automatically get access to same services that are available for persons with disabilities.

##### Policy Provisions Promoting Stakeholder Cooperation and Integration of Services

Differences exist in the degree of cooperation between healthcare and employment bodies in defining rehabilitation plans for persons with NCDs. At the stage of assessment, some countries still rely on a medical approach, without considering other factors (IT, SI, PL, EL). 

In terms of the cooperation between companies and healthcare professionals, countries like UK and Norway have return-to-work or long-term absence management mechanisms that enforces the link between these stakeholders. In this sense, the IA-Agreement in Norway is an example of the government’s attempt to ensure a greater involvement of employers. The Corporate Integration Management System (BEM) in Germany is another example.

##### Policy Provisions Promoting Persons-Centered Approach and Individualized Service Provision

The understanding of the importance of a person-centered approach and individualized services is seemingly shared by all countries, at least on paper. However, when it comes to the actual implementation of such services, a lot depends on the personnel of the employment services handling the case. Like in the case of Czech Republic, a lack of sufficient funding overwhelms labor offices, thus creating a risk that the needs of jobseekers are not adequately assessed.

##### Policy Provisions on Localized and Accessible Employment Service Provision

Mediterranean countries such as Italy and Spain have differences between regions in terms of supports and services available. While difference can be regarded as a possibility to provide more diverse and locally suited support mechanisms, it may also create unequal services for people depending on where they live. In the UK and Norway, employment services that operate through local branches, are in this sense more uniform. 

#### 3.2.2. National Systems

##### Employment Support in the Open Labor Market

Policies in all countries are targeted to integration into the open labor market. However, the effectiveness of such measures and the quality of their implementation can vary. 

Supported employment schemes are embedded in national policies and strategies in some countries (DE, AT, SI, ES) and takes different forms while keeping to the same principles in others. In Poland, for example, supported employment services are not mainstreamed but are rather available through individual projects (often funded by the European Commission).

##### Employment Support through Social Enterprises or Social Cooperatives

Social enterprises take different forms across countries. One of the most varied and business-focused forms of social economy presented in this study is the one of the UK. The market approach can be seen in the business approach and terminology that is used regarding social enterprises as they draw resources from social “investments”, rather than government “subsidies.” In some cases, social enterprises are so dependent on government support or not interested in economic sustainability, that their “commercial” activities can be questioned (e.g., some Vocational Rehabilitation Facilities in Poland).

##### Employment Support through Sheltered Work

In most countries, sheltered works are considered as the last resource for persons who are not able to be employed in the open labor market. They are often targeted at persons with (severe) intellectual/developmental disabilities and some people with mental health conditions but are not entirely relevant for persons with other chronic diseases.

##### Incentives for Persons with NCDs to Participate in Activation Programs

There are basically two ways in which persons with NCDs can be incentivized:

- Benefits that are conditional on participating in work-related activities (e.g., Work assessment Allowance, Qualification benefit, and Support when participating in measures in Norway; Employment and support allowance for certain recipients in the UK);
- Possibility to keep benefits while working. Most countries give this possibility depending on the degree of disability (e.g., DE, CZ, and ES).

##### Financial Incentives for Employers to Recruit/Retain Persons with NCDs

Most countries provide wage subsidies, nearly all temporary, with exception of the UK.

##### Non-Financial Incentives for Employers to Recruit/Retain Persons with NCDs

Non-financial incentives mainly take a form of corporate social responsibility commitments (CSR). Larger companies that have occupational health therapists also have a possibility to provide return-to-work programs and manage long-term sick leaves with an aim to have a positive impact on their workforce. In the UK and Norway, where there are no quota obligations or substantial financial incentives for employers, emphasis on non-financial incentives is important, such promoting diversity at workplace and highlighting the benefits of hiring and keeping persons with health problems.

##### Duties of Persons with NCDs

There are three main groups of countries regarding the responsibility of persons with disabilities (including NCDs) to participate in activation measures:

- Countries that have a rehabilitation-before-benefits rule (NO, UK);
- Countries that have rehabilitation-before-benefits provisions in place but are not (adequately) implemented (DE, AT);
- Countries that do not have the rehabilitation-before-benefits rule (All Mediterranean and Post-Communist model countries reviewed in this report).

##### Duties of Employers

Most countries have quota systems for persons with disability, thus some including people with NCDs, with exception of the United Kingdom and Norway.

#### 3.2.3. National Services

##### General and Specialized Employment Services for Persons with NCDs

Among the categories of chronic health conditions considered in this study, mental health condition is the one that most frequently has specialized services. Employment services for persons with mental health conditions are specific in a way that they require more psychological support and follow up from personal coaches. For the rest of the categories of NCDs, the services are less specialized and persons with those condition use general services available for persons with disabilities. Exceptions are made when patients’ associations, specialized in specific diseases, provide services to their target users (e.g., cancer associations, associations of persons with respiratory system problems). Such services usually focus on provision of information, support in coping strategies, etc.). A detailed description of all the services available per country is available on the website of the project (www.path-ways.eu) and as Supplementary Material to the manuscript.

## 4. Discussion

The objective of this study was to compare existing strategies for professional integration and reintegration of persons with chronic diseases available at both European and national level between different European regions. The mapping of policies, systems and services facilitating the inclusion of PwCDs at European and national level has revealed that in most countries individuals from this group are considered as part of a group of persons with disabilities, including persons with reduced work capacity due to illnesses. Persons with specific chronic health conditions considered in this study can mainly receive support in employment if their condition can be recognized as a “disability” in their countries (reaching a certain eligible degree of disability) or have a negative impact on their work ability. The fact that legislation for persons with disabilities does not always benefit people with chronic diseases could be related to the way how disability is defined in the country: the population that benefits from disability policies is considerably restricted for those countries that use a narrow definition of disability as a personal characteristic of a minority, while is broader for those countries adopting a more inclusive definition of disability, in line with the definition proposed by WHO [28]. Basing on the definition of disability of the Convention on the Rights of Persons with Disabilities, persons with disabilities are persons who have “long-term physical, mental, intellectual or sensory impairments which in interaction with various barriers may hinder their full and effective participation in society on an equal basis with others” [13]. Following this definition and due to the burden they experience in daily life, many persons with chronic diseases can be as well considered PwD. Persons with chronic conditions in fact experience considerable disability in daily life, ranging from problems in body functions to limitations in activities, and important restrictions in their participation in society.

The study shows that there is a general consistence between European and national legal frameworks regarding the activation of persons with disabilities and disadvantaged groups. Countries considered in this report do put in place provisions to support activation and greater labor market participation, but they do it in different ways.

In terms of policies, all countries have legislative frameworks against discrimination and provide some support to persons with disabilities and illnesses. Policy-level strategies targeted at activating PwCDs, are, on the other hand, more limited. They are targeted through strategies for broader groups (persons with disabilities, vulnerable social groups, elderly, etc.). Most policies highlight the significance of availability of mainstreamed, person-centered, integrated, and accessible employment services. However, the implementation of policies often does not go in line with the initial commitments, thus hampering the effectiveness of policies and programs. In addition, the existence of legal initiatives on work activation of PwCDs does not necessarily coincide with a change in attitudes towards their employment in the society. 

In terms of systems, countries differ from each other based on how much emphasis they put on supports, incentives or obligations in order to facilitate the integration of persons with disabilities and reduced work capacity. For instance, as an integration policy-oriented country, the UK provides fewer categorized support services, no financial incentives to employers in a form of wage subsidies and requires unemployed persons with reduced work capacity to participate in work-related activities. Norway, a Nordic welfare state, operates in a similar way, but it does provide wage subsidies to employers and provides a wide range of services aimed at empowering workers with health problems. Continental welfare states considered in this study have more categorization in terms of disability recognition, which makes the access to certain employment supports more difficult. These countries provide financial incentives and use quotas to activate employers but do not impose additional requirements on jobseekers. In Mediterranean welfare states the situation is fairly similar. Greece, however, due to financial difficulties, has very limited supports and activation measures. There, as well as in Post-Communist states considered in this report, funding from the EU plays an important role in providing support. 

In terms of services, the range of specialized services for most categories of chronic conditions is limited. Persons with chronic conditions receive mainstream employment services or services tailored for persons with disabilities or reduced work capacity. Out of all the categories of chronic conditions considered, for mental health conditions there are more specialized strategies in place. This may be explained by the markedly different needs of persons with such conditions and the fact that mental health has been high on the international agenda.

The recent difficult economic situation in Europe has led to the reduction in social protection expenditure and restricted the access to sickness and disability benefits in 2011 in most EU Member States [3]. A study by Saltman and others found that financial pressure and slower economic growth in Europe have led to decreased funding for healthcare and necessitated reforms to improve the sustainability of public funding of healthcare [29]. Financial pressure has led to reforms in pension schemes that aim to extend working life. Such reforms have made the withdrawal of older workers from the labor market in case of unemployment less likely than before [30].

It can be hypothesized that budgetary constraints and the impacts of the economic crisis have led to the contracting of the passive compensation-oriented policy and the expansion of the integration-oriented policy in European countries, although at different scales in different states. Despite having an overall tendency that is headed in the same direction—the direction of activation—the pathway of each country towards promoting employment integration is unique. Comparisons are difficult to make, due to differences among countries in cultural, historical, and economic backgrounds, in institutional and social settings, in approaches to chronic diseases and disabilities, etc. 

Moreover, a European study has identified that the supply of support in terms of adaptations of workplace and work content does not necessarily meet the needs of persons with chronic diseases [5]. In other words, not everybody with support needs is actually provided with such assistance. For example, in Belgium, 53% of workers with chronic diseases requested an adaptation of tasks, but only 34% of them obtained this support; in the Czech Republic 27.6% reported work adjustment needs but only 11.4% received support [5].

Another issue to be considered, is related to the ambivalent function of the social benefits, that can lead to the risk of the benefit trap, making people with ill-health more dependent on passive income supports and discouraging them from entering the labor market. The reduction of the labor force, in turn, has a negative impact on the economic growth. According to a study [31], there will be a potential shortfall of around 35 million workers, or about 15% of the total labor demand, by 2050. For this reason, it is important to ensure an inclusive labor market that would be able to meet the future labor demand and contribute to sustainable growth. Such inclusive markets can be made possible if every person at working age is given a possibility to participate in the open labor market and is provided with adequate support in doing so.

Participation of PwCDs in the open labor market can contribute to tackling the above-mentioned socio-economic challenges. As explained above, it has a potential to alleviate poverty and social exclusion, to encourage higher employment rates and labor supply, and to reduce public spending on disability benefits. Besides this, employment can have a positive impact on the well-being and mental health [32].

The results of our Pathways study are in line with previous one that confirms that, in addition to health-related obstacles, there are also non-medical factors that perpetuate long-term sick leaves and prevent persons with chronic conditions from returning to work, including personal, societal, and work-related obstacles [33]. Such factors include older age, lack of vocational rehabilitation counseling, and lack of cooperation from employers in modifying working conditions. In contrast, factors such as a better control of individual working conditions, personal guidance and support from health authorities and health professionals, and a positive attitude of the persons have been among factors that facilitate the return to work [33].

Therefore, the types of support provided to persons with NCDs in returning to or staying in employment should not be limited to health-related rehabilitation only but should encompass environmental adaptations and accommodations, thus adopting a biopsychosocial approach to employment.

Reduced unemployment, social equality, and higher labor market participation are among the main priorities set by the EU’s Europe 2020 strategy, in which the importance of participation of all working-age people regardless of their skill level in the labor market has been widely acknowledged [34]. To achieve inclusive and sustainable growth, everyone should be given an opportunity to enter and remain in the open labor market, including persons with NCDs. Hence, there is a need for implementing effective strategies to ensure their maintenance, integration, or reintegration in the labor market.

Furthermore, these results stressed the importance of employment for ensuring the quality of life of persons with chronic diseases and for achieving smart, sustainable, and inclusive growth under the Europe 2020 Strategy. Work in fact does not only have an impact on the quality of life of individuals, but also contributes to social cohesion by making people feel that they are part of society. 

Some limitations of this study should be mentioned. First, even though our search was extensive, it was not systematic, and we therefore cannot be sure that all relevant articles and reports were included. Second, the use of a convenience sampling to contact national stakeholders as respondents to questionnaires and interviews, can limit the generalizability of our results. Another limitation is related to the complexity of the situation in terms of employment strategies for persons with chronic diseases. We are aware that, within both the professional communities and the academic literature, there is a large degree of variation in the use of the term “chronic disease”, in the diseases that are included under the umbrella term “chronic disease”, and in the time a disease must be present for something to be referred to as chronic; this variation in meaning is amplified when viewed in an international context. Moreover, big differences exist between different categories of chronic diseases, individual diseases included in each category, as well as personal characteristics of each person with a chronic disease. On top of this, co-morbidities are also widespread. Including more conditions would have been ideal, but the number of CDs to be included could not be too broad because of the limitations imposed by the three years funding of the project. In this sense, the complexity of the topic and research scope was one of the main challenges of the present study. Finally, from a theoretical point of view, the definition of “welfare models” adopted in our project is complex, because a lot of factors exist that can modify these systems in a context where European social models face several challenges such as the financial sustainability, the globalization, and the social changes; again, the framework of our project imposed us to follow this categorization.

Despite these limitations, the study presents the following strengths: first, it provides a unique overview of employment strategies for persons with chronic diseases in various European countries and at different levels (policies, systems, services): identifying strategies in countries from different welfare models allowed exploration of potential commonalities and differences and identifying possible trends in the region. Second, it covers a wide range of chronic diseases, geographical locations, welfare models, levels of implementation (European and national). 

Our results constitute an important starting point, but further research is needed to explore in detail the efficacy of the existing strategies and study the peculiarity of each country and the specific pathway of different chronic diseases.

## 5. Conclusions

Statistics for Europe clearly show that NCDs pose a serious problem to society by negatively affecting labor market participation. The developmental risks associated with chronic diseases require high level policy intervention.

To a large extent, existing European and national policy frameworks on employment activation are not specifically targeted to accept a decrease in functioning of workers due to health conditions, thus allowing all (including PwCDs) to be included in employment. Instead, they target broader categories, such as persons with disabilities, long-term unemployed, vulnerable groups, etc. Emphasis should be made on the fact that strategies targeting persons with disabilities do not necessarily address the needs of patients with chronic diseases and mental health conditions since the employment needs of these groups are not always the same. Identifying the work-related needs of PwCDs and developing tailored interventions may be important for secondary prevention of illness becoming chronic. Furthermore, a more integrated and favorable service provision environment (employment support integrated with healthcare, social and psychological support), as well as more involvement from the part of employers, is crucial to promote a real inclusion of PwCDs in the labor market.

## Figures and Tables

**Table 1:** Features of European welfare models.

Model Name	Features	Country Examples
Scandinavian Model	Emphasis on egalitarianism and universal welfare provision [20]; Universal and generous benefits and a strong redistributive social security system [21,22]; Extensive fiscal intervention using active labor market policies, strong employment orientation [23].	Norway
Continental Model	Benefits tied to employment, financed mainly by employer and employee [24]; Minimal redistribution [24]; Social security is organized as insurance system [25].	Austria, Germany, Slovenia
Anglo-Saxon Model	Relatively large social assistance of the last resort [23]; Cash transfers are mainly oriented to people in working age [23]; Schemes conditioning access to benefits to regular employment and emphasis on activation measures [23]; A low level of government spending on social protection, modest benefits, usually means-tested [21,22]; Little redistribution of incomes [22]; High incidence of low-pay employment [23].	United Kingdom
Mediterranean Model	A dualist system of welfare provision, which strongly protects part of the population while under-protecting another [26]; High segmentation of entitlements and conditioned access to social provisions [27]; Welfare and social policies in fighting poverty are ineffective and fragmented [25]; Less generous benefits in comparison to the Continental model and not all the branches of social insurance are equally developed [25]; High dependence on informal, charitable, and family care [21].	Greece, Spain, Italy
“Post-Communist” Model	Generally low governmental spending on social programs, mostly financed through social contributions [26]; Relatively limited health service provision and poor overall population health system [21]; On-going transition process from institutional to community-based care [27]; Insufficient implementation and monitoring of the developed legislation, plans and strategies concerning the well-being of persons with disabilities [27]; Lower levels of governmental programs and the social situation [22]; Generally incoherent legal framework.	Czech Republic, Poland

**Table 2:** European level strategies: policies and their recipients.

Strategies Identified	Policies Supporting Employment (Re-)integration of Persons with Chronic Diseases Indirectly (i.e., PwCDs are Part of Broader Categories)		Policies Supporting Employment (Re-) integration of Persons with Chronic Diseases Directly	
	Policies Targeted at Persons with Disabilities ^1^	Policies Targeted at Other Categories	Policies Specifically Targeted at Persons with CD (CDs in General)	Policies Targeted at Specific Categories of CD (e.g., Mental Health ^2^, Neurological, Musculoskeletal, Respiratory, Cardiovascular, etc.)
EU Directive on Employment Equality 2000/78/EC	•	• Elderly		
Equal opportunities for people with disabilities: a European action plan (2004–2010)	•			
Disability Action Plan 2006–2015	•			
Community strategy 2007–2012 on health and safety at work	•	• Workers excluded from the workplace for a long period of time because of an accident at work, an occupational illness, or a disability		• Mental health
EU Strategic Framework on Health and Safety at Work 2014–2020	•		•	• Mental health
European Commission White Paper “Together for Health: A strategic approach for the EU 2008–2013”.		• People inactive due to ill-health		
Commission Recommendation 2008/867/EC on the active inclusion of people excluded from the labor market		• People excluded from the labor market		
European Disability Strategy 2010–2020	•			
Europe 2020: the European Union strategy for growth and employment	•	• Vulnerable workers, elderly		
Reflection Process on Chronic diseases: Final Report			•	
Council Recommendation on the integration of the long-term unemployed into the labor market		• Long-term unemployed		
European Accessibility Act	•			
Joint Action on Chronic Diseases (JA-CHRODIS)			•	
Green Paper on Improving the mental health of the population: Towards a strategy on mental health for the European Union				• Mental health
European Pact for Mental Health and Well-being				• Mental health
Joint Action Mental health and Well-being				• Mental health
European Parliament resolution of 19 February 2009 on Mental Health				• Mental health
Declaration of the European Parliament of 13 September 2012 on tackling multiple sclerosis in Europe				• Multiple sclerosis
CanCon Joint Action 2014–2017 (cancer)				• Cancer

Note: ^1^ In European policies, the definition of disability includes mental disability. In cases, where mental disability is included in the definition of disability, the strategies where not indicated separately in the last column of this table as “Policies targeted at specific categories of CD”.^2^ Mental health is included in this column only when mentioned specifically, separately or outside the broader definition of disability. PwCDs: Persons with chronic diseases, CD: chronic diseases. CanCon: Cancer Control.

**Table 3:** National level strategies: Policies and systems.

Strategies Identified	Nordic	Continental			Anglo-Saxon	Mediterranean			“Post-Communist”	
	NO	AT	DE	SI	UK	EL	ES	IT	CZ	PL
Legislative frameworks specifically on chronic diseases and employment										
Legislative frameworks on mental health and employment	•				•					
Legislative frameworks on disability and employment	•	•	•	•	•	•	•	•	•	•
Mainstream and specialist employment programs	•	•	•	•	•	•	•	•	•	•
Provisions allowing access to employment rehabilitation support for persons with NCD, without making disability a prerequisite	•				•					
Policy provisions on stakeholder cooperation (e.g., healthcare, employment services, social services, employers)	•		•		•					
Policy provisions on persons-centered approach and individualized employment service provision	•	•	•	•	•	•	•	•	•	•
Employment support in the open labor market (Supported employment)	•	•	•	•	•	•	•	•	•	•
Employment support through social enterprises or social cooperatives;	•	•	•	•	•	•	•	•	•	•
Employment support through sheltered work;	•	•	•	•		•	•	•	•	•
Incentives for persons with NCDs to participate in activation programs;	•	•	•	•	•	•	•	•	•	
Financial incentives for employers to recruit/retain persons with NCDs (wage subsidy);	•	•	•	•		•	•	•	•	•
Non-financial incentives for employers to recruit/retain persons with NCDs;	•	•	•		•	•	•	•	•	•
Obligatory participation in activation programs to receive benefits;	•	•	•		•					
Duties of employers (e.g., quota systems).		•	•	•		•	•	•	•	•

Note: (AT) Austria, (CZ) Czech Republic, (DE) Germany, (EL) Greece, (IT) Italy, (NO) Norway, (PL) Poland, (SI) Slovenia, (ES) Spain, (UK) United Kingdom, NCD: non-communicable diseases.

## Supplementary Materials

The following are available online at http://www.mdpi.com/1660-4601/15/4/781/s1, File S1: The full report “Comparison of available strategies for professional integration and reintegration of persons with chronic diseases and mental health. Report based on five categories of social welfare models in Europe”, that includes the detailed reports for each Country, is available online at www.path-ways.eu.

## Appendix A

**Table A1:** Questionnaire respondents.

Country	Organization	Position
AT	Österreichische Diabetikervereinigung	Chairwoman at national level
	Österreichischer Herzverband (Landesverband Kärnten)	President at regional level in Kärnten (retired)
	Österreichische Krebshilfe Wien	Managing director of Krebshilfe
	SHÖ Schlaganfallhilfe Österreich	Chairwoman SHÖ—Schlaganfallhilfe Österreich (stroke help Austria) (retired)
	Pro mente oberösterreich	Head of “pro mente arbeit” (branch of the organization addressing issues around mental health issues and work/profession)
	Ottirol umbrella organization for support groups	Head of regional umbrella association of support groups (Osttirol)
	Support group headaches	Assistant of managing director (head of support group)
	Behindertenanwaltschaft	“Behindertenanwalt” on national level
	Dachverband Selbsthilfe Kärnten	Managing director
CZ	General Labor Office of Czech Republic	Representative
	NGO (Non-Governmental Organization) Cerebrum	Representative
	Government Committee for people with disabilities in Czech Republic	Representative
	Department of Rehabilitation Medicine	Representative
	Occupational therapists	Occupational therapists
	Department of Rehabilitation Medicine	Head physician
	First Medical Faculty	Head of education
	Rehalb Health & Medical	Director
	Prague committee of wheelchairs users	User
	Department of Rehabilitation Medicine, founder of prevocational rehabilitation	Former head
DE	Südwestfälische Industrie- und Handelskammer zu Hagen (SIHK) (South Westphalian Chamber of Industry and Commerce in Hagen)	Inclusion consultant
	CBP—Caritas Behindertenhilfe und Psychiatrie e.V. (Caritas Mental Help and Psychiatry)	Lawyer, Deputy Director
	Sozialverband Deutschland (SoVD) (Social Association of Germany)	Lawyer, Deputy Director of the Disability Policy Department
	Bundesarbeitsgemeinschaft Integrationsfirmen e.V. (bag if) (National Working Group Integrative Companies)	Social worker, managing director
	Bundesagentur für Arbeit (Federal Employment Agency)	Physician in the department Medical Service
EL	Department of Special Education, University of Thessaly	Professors and Researcher A
	Manpower Employment Organization—Employment Office for Special Social Groups	Job consultant
	The Greek Ombudsman	Scientific staff—Human Rights Department
	Panhellenic Union for the Psychosocial Rehabilitation and Work Integration	Employment counsellor
	Multiple Sclerosis Panthessalic Union	Secretarial and administrative support - unemployed
	Panhellenic Federation of Unions-Associations of persons with Diabetes Mellitus	Biologist
	Bone Health Society “Butterfly”	General Practitioner
	Association “Hellenic Pulmonary Hypertension”	President of the Association “Hellenic Pulmonary Hypertension”
	Social Cooperative Firm “La petite cantine”	Representative of a Social Cooperative Firm
	Greek Anticancer Society	General Practitioner
ES	Instituto de Mayores y Servicios Sociales (Institute for the Elderly and Social Services IMSERSO)	Worker of the State General Administration
	Asociación Española Contra el Cáncer (aecc) (Spanish Association Against Cancer (AECC))	Social worker
	Intecserveis Centros Especiales de Trabajo (CET) (Fundació Germà Benito Menni) (Brother Benito Menni Foundation)	Social worker
	Parc Sanitari Sant Joan de Déu (PSSJD)	Job developer/Labor insertor
	Parc Sanitari Sant Joan de Déu (PSSJD)	Neurologist
	Provincial Directorate of the National Social Security Institute	Provincial Deputy Director of Support and Information of the National Institute of Social Security Barcelona
	APACOR—Asociación De Pacientes Coronarios (Association of coronary patients)	Retired Voluntareer
	Fundación Lovexair (Lovexair foundation)	Psychologist
	PSSJD	Social Worker
	Servicio Andaluz de Saluz (SAS) (Andalusian Healthcare Service)	Doctor
	La Paz Hospital	Traumatologist
	Virgen del Rocio Hospital	Neurologist
	Foundation Carmen Pardo—Valcarce	Director of Employment services
	Hospital La princesa	MD
	Hospital La princesa	MD, Respiratory unit
	Universidad Autónoma de Madrid and Hospital La princesa	Psychiatrist
	Hospital La princesa	MD
IT	Fondazione IRCCS, Istituto Neurologico Carlo Besta	Social worker
	LEDHA (Disabled persons’s rights Association)	Social worker
	A.O. Luigi Sacco—Hospital	Psychiatric Consultant
	A.O. Mellino Mellini—Hospital	Mental Health Department Director
	A.O. San Carlo Borromeo—Hospital	Neurologist, Hospital Unit Director
	ADPMI (Associazione Diabetici della Provincia di Milano) Association for patients with diabetes in Milan	President of the Milan Diabetes Association and Coordinator
	Rehabilitation Centre Villa Beretta—Ospedale Valduce	Social worker
	AIMaC, (Associazione Italiana Malati di Cancro) Italian Association for patients with cancer, caregivers and friends	Vice President
	U.O. Broncopneumologia, A.O. di Busto Arsizio—Tradate—Saronno (Hospital, Bronchopneumology Unit)	Healthcare assistent
	AICCA Onlus, Associazione Italiana dei Cardiopatici Congeniti Adulti. (Italian association of congenital heart patients)	Counselor—Peer Counselor
NO	Norwegian Labour and Welfare Administration (NAV) Buskerud	Advisor for persons with chronic diseases, HR (human resources) management
	NAV Hamar	Advisor
SI	Ministry of Labor, Family, Social Affairs and Equal Opportunities (Ministrstvo za delo, družino, scialne zadeve in enake možnosti)	Secretary at the Ministry
	Employment Service of Slovenia (Zavod Republike Slovenije za zaposlovanje)	Adviser
	University Rehabilitation Institute, Republic of Slovenia (Univerzitetni rehabilitacijski inštitut Republike Slovenije—Soča	Head of Development Centre of Vocational Rehabilitation
	Šentprima—Institute for Rehabilitation and Education (Šentprima—Zavod za svetovanje, usposabljanje in rehabilitacijo invalidov)	Director
	SONČEK—Cerebral Palsy Association of Slovenia (SONČEK—Zveza društvev za cerebralno paralizo Slovenije)	Social worker
	Federation of Disabled Workers of Slovenia (Združenje delovnih invalidov Slovenije)	President
	Slovenian Paraplegic Association	Project/program manager
	ŠENT (Slovensko združenje za duševno zdravje)—Slovenian Association for Mental Health (ŠENT—Slovensko združenje za duševno zdravje)	President of ŠENT Users Council of people with mental health problems
	Muscular Dystrophy Association of Slovenia (Društvo distrofikov Slovenije)	Secretary
	Centerkontura; Slovenian Association of Vocational Rehabilitation Providers	Director of Centerkontura (vocational rehabilitation service provider) and president of Slovenian Association of Vocational Rehabilitation Providers
PL	Defeat cancer	Chemist
	Jagiellonian University	University Disability Officer, head of the Jagiellonian University Disability Support Service
	Association for Development Community Psychiatry and Care (Stowarzyszenie na Rzecz Rozwoju Psychiatrii i Opieki Środowiskowej)	Psychiatrist
	The University Hospital	Physician
	Department of Metabolic Diseases, The University Hospital	Physician
	I Chair of General Surgery and Department of General Surgery, Oncology and Gastroenterology—Jagiellonian University Medical College	Physician
	National Union of the Vocational rehabilitation facilities and public enterprise	Manager
	MATIO Foundation for Families and Patients with Cystic Fibrosis	Physiotherapist
	Jagiellonian University Medical College	Physician
	Municipal labor office—department of stimulation of disabled people	Client advisor—job placement agent

Note: SoVD: Sozialverband Deutschland; IRCCS: Istituto di Ricovero e Cura a Carattere Scientifico.

## Appendix B

**Table A2:** Interview respondents.

Country	Organization	Occupation
AT	Austrian Chamber of Labor (Arbeiterkammer), Department of Labor Market and Integration	Expert
	Austrian Chamber of Labor (Arbeiterkammer), Department of Social Insurance	Expert
	Anwaltschaft für Menschen mit Behinderung	Expert
CZ	CEREBRUM—Brain Injured and their Families Czech Republic	Representative
	The Labor Office of the Czech Republic—General directorate	Representative
	Government committee for persons with disabilities	Head
DE	Klinikum der Universität München (University Hospital of Munich)	Head of the department—Corporate Integration Management
	Christlich-Soziale Union (CSU) (political party Christian Social Union)	Regional Chair of the Health Policy Working Group
	Berufsförderungswerk München (Vocational (Re)training Center Munich)	Director of Vocational Training
EL	EDRA Social Cooperative Action for Vulnerable Groups	Representatives
	National Confederation of People with Disabilities	Member
	Special Service for Social Inclusion and Social Economy (EYKEKO)	Representative
ES	Madrid’s health area	Psychiatrist, responsible for the continuity
	User, owner of enterprise of breast and feat prosthesis	Psychologist
	Mental Health Regional Office of Madrid	Deputy Director
	Patient with Obsessive Compulsory Disease	User
	Job placement service of Esplugues de Llobregat	Individual Placement and Support Programme Worker
	Guidance Insertion Service for People with Disabilities, Sant Boi de Llobregat Council	Guidance service team
IT	Ministry of Labor and Social Policy	Representative
	A&I—Onlus	Member
	AISM (Italian Multiple Sclerosis Society)	User
NO	“Stop the Discrimination”	Activist in disabled people’s movement
	Norwegian Welfare Administration (NAV)	Job counsellor
	Norwegian Welfare Administration (NAV)	Sociologist
PL	Marschal’s Office of Malopolska Voivodeship	Representative
	Foundation Activation	Representative
	Polish Association of Disabled People, University of John Paul II in Krakow	Psychologist and career counselor
SI	Cveto Uršič	Representative
	Slovenian Association of Vocational Rehabilitation Service Providers	President of the Association
	Association of Persons with Disabilities	Vocational rehabilitation service user
UK	Remploy	Representatives
