# Supplementary material for: Mapping European Welfare Models: State of the Art of Strategies for Professional Integration and Reintegration of Persons with Chronic Diseases
Source: Int J Environ Res Public Health. 2018 Apr 17;15(4):781. doi: 10.3390/ijerph15040781 (PMC5923823; doi:10.3390/ijerph15040781)
Supplement: Supplementary file 1 [file ijerph-15-00781-s001.pdf]

Participation To Healthy Workplaces And inclusive Strategies in the Work Sector

PATHWAYS PROJECT

COMPARISON OF AVAILABLE STRATEGIES FOR PROFESSIONAL INTEGRATION AND REINTEGRATION OF PERSONS WITH CHRONIC DISEASES AND MENTAL HEALTH

REPORT BASED ON FIVE CATEGORIES OF SOCIAL WELFARE MODELS IN EUROPE

PATHWAYS project has received funding from the European Union's Health Programme (2014-2020)

Grant agreement n. 663474

www.path-ways.eu

Fondazione IRCCS Istituto Neurologico Carlo Besta – Sistema Sanitario Regione Lombardia
EASPD – Improving services improving lives
University of Thessaly
Jagiellonian University in Kraków
Oslo and Akershus University College of Applied Sciences
LMU Ludwig-Maximilians-Universität München
KABEG Gailtal-Klinik
Parc Sanitari Sant Joan de Déu
Carinthia University of Applied Sciences – Fachhochschule Kärnten
UAM Universidad Autonoma de Madrid
University Rehabili[illegible] Republic of Slovenia
Všeobecná fakultní nemocnice v Praze

PATHWAYS PROJECT

PArticipation To Healthy Workplaces And inclusive Strategies in the Work Sector

Deliverable number 4.1

Deliverable title :

REPORT ON THE COMPARISON OF THE AVAILABLE STRATEGIES FOR PROFESSIONAL INTEGRATION AND REINTEGRATION OF PERSONS WITH CHRONIC DISEASES AND MENTAL HEALTH ISSUES

BASED ON FIVE CATEGORIES OF SOCIAL WELFARE MODELS IN EUROPE

**Deliverable type**	Report
**Deliverable responsible partner**	EASPD
**Contractual date of delivery**	Month 12
**Actual date of delivery**	Month 12
**Dissemination level**	Public
**Status of deliverable**	Submitted

List of abbreviations

AAP	Work assessment allowance, Arbeidsavklaringspenger (Norway)
ADL	Activities of daily living
AMS	Public Employment Service of Austria
ANED	Academic Network of European Disability experts
AVRE	Association of Vocational Rehabilitation Enterprises (Norway)
CEE	Central and Eastern Europe
CELAV	Centre for Job Coaching (Italy)
COPD	Chronic obstructive pulmonary disease
DWP	Department for Work and Pensions (UK)
EASPD	European Association of Service providers to Persons with Disabilities
ESA	Employment and support allowance (UK)
ESF	European Social Fund
EU	European Union
EU-OSHA	European Agency for Safety and Health at Work
EU-SILC	European Statistics of Income and Living Condition
EUR	Euro
GBP	British pound
GDP	Gross domestic product
IA Agreement	Inclusive Work Environment Agreement
ICF	International Classification of Functioning, Disability and Health
ILO	International Labour Organisation
IPS	Individual Placement and Support
KoinSEp	Social Cooperative Enterprises (Greece)
KoiSPE	Limited Liability Social Cooperatives (Greece)
LFS	Labour Force Survey
LHPAD	Longstanding health problem and/or a basic activity difficulty
MSD(-s)	Musculoskeletal disorder(-s)
NAV	Norwegian Labour and Welfare Administration
NCD(-s)	Non-communicable disease(-s)
NGO	Non-governmental organisation
NOK	Norwegian krone
OAED	Manpower Employment Organisation (Greece)
OECD	Organisation for Economic Co-operation and Development
PES	Public Employment Service
PFRON	State Fund for Rehabilitation of Disabled Persons (Poland)
PLN	Polish złoty
SMS	Sozialministeriumservice (Austria)
TOPEKO	Local Actions for Social Integration of Vulnerable Groups (Greece)
UN	United Nations
UNCRPD	United Nations Convention on the Rights of Persons with Disabilities
VRF(-s)	Vocational Rehabilitation Facility(-ies)
WHO	World Health Organisation
YLL	Years of life lost
YLD	Years lost to disability

Country abbreviations

AT	Austria	ES	Spain	MT	Malta
BE	Belgium	FI	Finland	NL	Netherlands
BG	Bulgaria	FR	France	NO	Norway
CH	Switzerland	HR	Croatia	PL	Poland
CY	Cyprus	HU	Hungary	PT	Portugal
CZ	Czech Republic	IE	Ireland	RO	Romania
DE	Germany	IT	Italy	SE	Sweden
DN	Denmark	LT	Lithuania	SI	Slovenia
EE	Estonia	LU	Luxemburg	SK	Slovakia
EL	Greece	LV	Latvia	UK	United Kingdom

Executive Summary

Chronic diseases, or non-communicable diseases (NCDs), are broadly defined by the World Health Organization (WHO) as diseases of long duration and generally slow progression that are not passed from person to person[1]. NCDs pose a serious threat to society and future development. Long-term health problems constitute to a greater risk of income poverty, social exclusion, severe material deprivation, and lower work intensity (ANED, 2013). Persons with longstanding health problem face higher rates of unemployment and inactivity (Corral et al., 2014). Based on the data of the 2011 ad hoc module of the EU Labour Force Survey, the employment rate in EU-28 for persons with limitations in work caused by a health condition was 29.6 percentage points less than for people with no such limitations[2].

This need for implementing strategies helping persons with chronic diseases to stay in employment is reflected in the European Union's Europe 2020 strategy, which highlights the importance of participation of all working-age people regardless of their skill level in the labour market. To achieve inclusive and sustainable growth, everyone should be given an opportunity to enter and remain in the open labour market, including persons with NCDs.

Report objective

The objective of this report is to map various strategies for professional (re-)integration of persons with chronic diseases and mental health issues available at both European and national level. Strategies considered in this study include strategies at the level of policies, systems, and services. The mapping of professional (re-)integration strategies carried out in this report is expected to lead in the later phases of the PATHWAYS project to the development of guidelines supporting the implementation of effective professional (re-)integration strategies for persons with NCDs (read more about PATHWAYS here: www.path-ways.eu)

Report scope

The following seven categories of NCDs were selected based on their contribution to *years lost to disability* (using the 2012 estimates of the WHO): mental health issues[3], neurological diseases (with

[1] http://www.who.int/mediacentre/factsheets/fs355/en/
[2] Source: Eurostat, hlth_dlm010 2011, both sexes, age group of 15-64 years.
[3] The term 'mental health issues' is used in this report to replace the term 'mental disorders'. Despite being widely used in literature, the term 'mental disorders' is not in line with the principles of the UN Convention on the Rights of Persons with Disabilities.

the focus on headache disorders), metabolic disorders, musculoskeletal disorders (MSDs), respiratory diseases, cardiovascular diseases (CVDs) and cancer.

The countries considered in the study are: Austria, Czech Republic, Germany, Greece, Italy, Norway, Poland, Slovenia, Spain and the United Kingdom. The countries represent one of five European welfare models: Scandinavian, Continental, Anglo-Saxon, Mediterranean, and "Post-Communist" models. Identifying strategies in countries from different welfare models allows exploring potential commonalities and differences and identifying possible trends in the region.

European policy frameworks

A review of European policies has revealed that to a large extent the employment activation of persons with NCDs is implemented through:

- Policy frameworks on the employment of persons with disabilities (e.g. EU Directive on Employment Equality 2000/78/EC, European Disability Strategy 2010-2020);
- Policy frameworks on employment activation and inclusion in the labour market (e.g. Europe 2020: the European Union strategy for growth and employment, Council Recommendation on the integration of the long-term unemployed into the labour market, Commission Recommendation 2008/867/EC on the active inclusion of people excluded from the labour market).

Policy provisions specifically focusing on the professional (re-)integration of persons with NCDs are often part of broader policy frameworks. For example, the EU Strategic Framework on Health and Safety at Work 2014-2020 specifically mentions supports in recruitment and return to work of people with a chronic or rare disease, disability or mental issues, and the use of integrated employment measures such as individualised support, counselling, guidance, access to general and vocational education and training, and other.

There is also a number of policy reports and actions specifically targeted at chronic diseases or at particular chronic conditions (e.g. Reflection Process on Chronic diseases: Final Report, Joint Action on Chronic Diseases (JA-CHRODIS), Green Paper on Improving the mental health of the population, Joint Action Mental health and Well-being, CANCON Joint Action 2014-2017).

National strategies

Policy approaches set at European level are certainly reflected at national-level polices, too. Budgetary constraints and the impacts of the economic crisis have led to the contracting of the passive compensation-oriented policy and the expansion of the integration-oriented policy in European countries, although at different scales in different states. Despite having an overall

tendency that is headed in the same direction – the direction of activation - the pathway of each country towards promoting employment integration is unique. Comparisons are difficult to make due to differences among countries in cultural, historical and economic backgrounds, in institutional and social settings, in approaches to chronic diseases and disabilities, etc.

The mapping of strategies for professional (re-)integration of persons with NCDs in the ten selected countries has been carried out following the structure outlined below:

Policies:

- Availability of legislative frameworks on chronic diseases, mental health and employment;
- Availability of legislative frameworks on disability and employment;
- Policy provisions on mainstream and specialist employment programmes;
- Policy provisions on access to employment support;
- Policy provisions promoting persons-centred approach and individualised service provision;
- Policy provisions on localised and accessible employment service provision;

Systems:

- Employment support in the open labour market;
- Employment support through Social enterprises or social cooperatives;
- Employment support through sheltered work;
- Incentives for persons with NCDs to participate in activation programmes;
- Financial incentives for employers to recruit/retain persons with NCDs;
- Non-financial incentives for employers to recruit/retain persons with NCDs;
- Duties of persons with NCDs to participate in activation programmes;
- Duties of employers (e.g. quota systems);

Services:

- Availability of general and specialised employment services for persons with NCDs.

The mapping of policies, systems and services facilitating the inclusion of persons with NCDs has revealed that in most cases, people from this group are considered as part of a group of persons with disabilities, including persons with reduced work capacity due to illnesses. In many cases, persons with chronic health problems are eligible for specialised support in employment only when their condition is recognised as a disability (reaching a certain eligible degree of disability) or has a negative impact on their work ability, depending on national and regional regulations.

The study shows that countries considered in this report do put in place provisions to support activation and greater labour market participation by vulnerable groups, but they do it in different ways.

In terms of policies, all countries have legislative frameworks against discrimination and provide some support to persons with disabilities. Policy-level strategies targeted at activating persons with chronic diseases, are, on the other hand, more limited. They are targeted through strategies for broader groups (persons with disabilities, vulnerable social groups, elderly, etc.). Most policies highlight the significance of availability of mainstreamed, person-centred, integrated and accessible employment services. However, the implementation of policies often does not go in line with the initial commitments, thus hampering the effectiveness of policies and programmes. In addition, the existence of legal initiatives on work activation of persons with chronic conditions does not necessarily coincide with a change in attitudes towards their employment in the society.

In terms of systems, countries differ from each other based on how much emphasis they put on supports, incentives or obligations in order to facilitate the integration of persons with disabilities and reduced work capacity. For instance, as an integration policy-oriented country, the UK provides less categorised support services, no financial incentives to employers in a form of wage subsidies, and requires unemployed persons with reduced work capacity to participate in work-related activities. Norway, a Nordic welfare state, operates in a similar way, but it does provide wage subsidies to employers and provides a wide range of services aimed at empowering workers with health problems. Continental welfare states considered in this study have more categorisation in terms of disability recognition, which makes the access to certain employment supports more difficult. These countries provide financial incentives and use quotas to activate employers but do not impose additional requirements on jobseekers. In Mediterranean welfare states the situation is fairly similar. Greece, however, due to financial difficulties has very limited supports and activation measures. There, as well as in Post-Communist states considered in this report, funding from the EU plays an important role in providing support.

In terms of systems, the range of specialised services for most categories of chronic conditions is limited. Persons with chronic conditions receive mainstream employment services or services tailored for persons with disabilities or reduced work capacity. Out of all the categories of chronic conditions considered, for mental health issues there are more specialised strategies in place. This may be explained by the markedly different needs of persons with such conditions and the fact that mental health has been high on the international agenda.

Recommendations

Based on the findings of the mapping exercise, the following recommendations can be made:

More focus on chronic diseases:

- The growing prevalence of chronic diseases and their impact on productivity and labour market participation necessitates an increased awareness of the need for extensive policy level strategies for the inclusion of persons with chronic conditions in employment.
- Emphasis should be made on the lack of policy-level strategies specifically targeting the employment activation of persons with NCDs and on the fact that strategies targeting persons with disabilities do not necessarily address the needs of patients with NCDs since the employment needs of these two groups are not the same.
- More research is needed into the effectiveness of existing strategies for (re-)integration of persons with disabilities when these are used for persons with NCDs.
- More research is needed into the needs of persons with different chronic diseases in employment support.
- Innovative and needs-based programmes and support measures for professional (re-)integration of persons with NCDs should be put in place.

A more integrated and favourable service provision environment:

- Persons with NCDs require integrated employment services because their needs encompass different areas such as healthcare, social and psychological support. Therefore, provisions for integrated employment support should be promoted.
- Better coordination between healthcare and employment should be ensured in order to ensure a better understanding of the relationship between work and chronic diseases.
- Health professionals should be trained to provide work-related advice to persons with NCDs to facilitate a quicker return to work.
- Better cooperation is also needed between employers and healthcare and employment service professionals as employers need to be informed about specific health-related needs of workers in terms of work adjustments and overall inclusion in the labour force.
- Persons with NCDs should be able to access adequate and customised employment services in their localities. In addition, measures should be taken to ensure equal level of services availability in different regions.

- Provisions should be put in place to promote the development and availability of public and private employment service provision, ensuring the link between the sectors through cooperation.
- Systems facilitating employment (re-)integration of persons with NCDs should not consider support programmes, systems of incentives and systems of obligations as separate elements. Instead, these should be seen as part of a comprehensive strategy.
- Emphasis should be made on employment in the open labour market. The move away from unsustainable sheltered work should be encouraged towards sustainable work.
- Labour market policies should be made more flexible with regard to the entry and exit of persons with NCDs and disabilities since the rigidity of overly protective measures may discourage employers from employing persons with health conditions.
- Support in financing workplace adjustments (including removal of physical and non-physical barriers) should be available not only to persons with a recognised disability but also to persons diagnosed with NCDs.
- Support measures such as job coaching, mentoring, counselling should be available for persons at all stages of employment (i.e. finding, getting and staying at a job).
- The economic sustainability of social enterprises should be encouraged through measures creating favourable conditions for competitive and commercial activity as well as through the availability of social investments.
- Sheltered workshops should be transformed in order to target the transition towards the open labour market.

More empowerment for persons with NCDs:

- Employees and jobseekers with NCDs should be better informed about their work-related rights and about the availability of support. In most cases, employed persons who acquire chronic conditions often do not need basic labour market integration training but rather need help in understanding their conditions and the ways in which they can cope with the barriers created by their conditions in the work environment. In such cases, support from psychologists or peers with similar conditions may prove more useful.
- Legislative frameworks should ensure the accessibility of existing employment support to persons with NCDs. The eligibility criteria for employment support need to be more flexible and not conditional on disability certification, especially if the assessment is based on the medical approach to disability.

- Policy provisions should focus on the capacity to work and on the tasks that persons with NCDs can perform rather than on the inability to work.
- Specialised support to persons with reduced work capacity, including persons with NCDs, should not be segregated from mainstream employment services. The segregation may lead to the false perception by employers and jobseekers themselves that persons with NCDs and persons with disabilities in general are of lower grade.
- The person-centred approach towards supported employment service provision needs to be further promoted since, as mentioned above, persons with different chronic conditions may have different employment needs. Furthermore, supported employment should be explicitly made available for persons with NCDs.
- Persons with NCDs should be encouraged to first participate in employment activation programmes before considering to become passive recipients of disability benefits.
- Passive benefits payment should be avoided when there are other options as there is evidence that in countries with no permanent or temporary disability benefits, the inactivity rate among people with chronic diseases is lower (OECD, 2007).
- Measures need to be put in place to financially support persons with NCDs during the time when they participate in employment activation programmes.
- Measures allowing persons with NCDs to keep disability or similar benefits while working need to be in place. Persons with NCDs may receive lower salaries or less salary due to a reduced working capacity and therefore, they may still need welfare support. The possibility to combine work and financial assistance is important for avoiding the benefit trap.

More involvement from the part of employers:

- Policies and systems should focus on a greater involvement of employers as they are the ones who at the end of the day should provide employment to persons with NCDs. Their cooperation is vital and there is a need for developing strong, innovative and consistent strategies for ensuring their commitment to the inclusion of persons with NCDs.
- The efficiency of financial incentives paid to employers in the form of wage subsidies and tax reductions should be further explored with regard to persons with chronic conditions.
- Efforts to increase the understanding among employers about operational benefits of employing and keeping persons with NCDs should be further promoted with emphasis on advantages for businesses such as the availability of a larger pool of talents, greater work

commitment and loyalty, a possibility to increase a customer base by having a more diverse staff, corporate social responsibility, a greater work-satisfaction among the workforce, etc.

- Further exploration is needed of the applicability and effectiveness of the quota system for the integration of persons with NCDs in work.
- The needs of both employers and (potential) employees should be equally considered for effective job matching, which means that job matching should not be subject to unnecessary impositions and mandatory placements that disregard task-related requirements.
- Services aimed at supporting awareness raising and training for staff and management to better understand the needs of persons with NCDs should be strongly encouraged.
- More services for employers should be offered to better manage illness-related long-term absences and return-to work mechanisms.

Table of content

I. Introduction **16**
II. Analytical Framework **20**
2.1. Definition of main concepts 20
2.2. Explaining relationships between concepts 21
2.3. Description of categories of chronic diseases 22
2.4. Description of European welfare models 27
III. European strategies **30**
3.1. Chronic diseases and employment: European state of play in numbers 30
3.2. European policy frameworks on the employment of persons with chronic diseases 36
3.3. EU funding programmes 42
3.4. Policy tendencies in Europe 43
IV. National strategies **45**
4.1. Scandinavian model 48
4.1.1. Norway 48
4.2. Anglo-Saxon model 57
4.2.1. United Kingdom 57
4.3. Continental model 65
4.3.1. Austria 65
4.3.2. Germany 73
4.3.3. Slovenia 80
4.4. Mediterranean model 88
4.4.1. Greece 88
4.4.2. Italy 98
4.4.3. Spain 110
4.5. "Post-Communist" model 119
4.5.1. Czech Republic 119
4.5.2. Poland 126
4.6. Comparative analysis 134
V. Conclusions and recommendations **140**

Bibliography **144**

Annexes

Annex 1: Questionnaire and interview respondents 150
Annex 2: Questionnaire template 154
Annex 3: Interview guidelines 161
Annex 4: Statistical data on prevalence of and mortality from chronic diseases 164
Annex 5: Employment rate for chronic disease sub-categories 169
Annex 6: Descriptive statistics for seven disease categories 172
Annex 7: International frameworks 188

// Acknowledgments

Acknowledgments

The European Association of Service providers for Persons with Disabilities (EASPD) would like to thank all the partners of the PATHWAYS project for their contribution to this report:

- Fondazione IRCSS Istituto Neurologico Carlo Besta, Milan, ITALY
- Universidad Autónoma de Madrid, Madrid, SPAIN
- Parc Sanitari Sant Joan de Déu, Barcelona, SPAIN
- Panepistimio Thessalias, Volos, GREECE
- University Rehabilitation Institute, Ljubljana, SLOVENIA
- Hogskolen I Oslo Og Akershus, Oslo, NORWAY
- Gailtal Klinik - Neurologische Rehabilitation, Hermagor, AUSTRIA
- Uniwersytet Jagiellonski, Krakow, POLAND
- Ludwig-Maximilians-Universitaet Muenchen, Munich, GERMANY
- Vseobecna fakultni nemocnice v Praze, Praha, CZECH REPUBLIC
- Carinthia University of Applied Sciences, Klagenfurt, AUSTRIA

In addition, we would like to thank all the questionnaire and interview participants for sharing their knowledge and experiences.

This report also benefitted from the efforts of Erik H. Samoy, who holds a PhD in sociology from the Catholic University of Louvain in Belgium (KU Leuven) on employment policies for the disabled and has a lifelong experience in social research.

Brussels, 2016

I. Introduction

People with chronic diseases, or non-communicable diseases (NCDs), face difficulties in employment due to various issues, including absence from work due to medical treatment or health conditions limiting their ability to perform certain tasks. The current economic crisis and rising unemployment in Europe makes it even more challenging for all people, including persons with NCDs, to find or retain jobs. In order to improve the participation of persons with chronic diseases in the labour market, it is important to have in place adequate strategies facilitating their professional (re-) integration. The aim of this report is to identify these strategies at European and national level. The report is part of the PATHWAYS project (for more information about the project please consult www.path-ways.eu).

NCDs are broadly defined by the World Health Organisation (WHO) as diseases of long duration and generally slow progression that are not passed from person to person[4]. This report focuses on the following categories of NCDs: mental health issues[5], neurological diseases (with the focus on headache disorders), metabolic disorders, musculoskeletal disorders (MSDs), respiratory diseases, cardiovascular diseases (CVDs) and cancer. The categories have been selected based on their impact on labour market participation.

NCDs pose a serious threat to society and future development as they are responsible for more deaths than any other causes of death taken together. In 2012 alone, about 38 million people died from NCDs, which represents about 68% of all deaths in the world (WHO, 2014: 9). The incidence of NCDs is expected to rise in the future due to the ageing of the European population, given the fact that elderly people are highly affected by chronic diseases. According to WHO estimates, in 2030, chronic diseases will be responsible for 52 million deaths worldwide (ibid.).

NCDs are also strongly associated with societal inequality. The burden and risks of NCDs have been internationally acknowledged as one of the "major challenges for development in the twenty-first century" in the United Nations' (UN) Political Declaration on the Prevention and Control of Non-communicable Diseases (UN, 2011: 1). According to a report of the Academic Network of European Disability experts (ANED, 2013), persons with long-term health problems face a greater risk of

[4] http://www.who.int/mediacentre/factsheets/fs355/en/

[5] The term 'mental health issues' is used in this report to replace the term 'mental disorders'. Despite being widely used in literature, the term 'mental disorders' is not in line with the principles of the UN Convention on the Rights of Persons with Disabilities.

income poverty, social exclusion, severe material deprivation, and lower work intensity. The same is confirmed by the study of the Organisation for Economic Co-operation and Development (OECD, 2010) on sickness, disability and work. The socio-economic impact of chronic diseases on development goals is evident since poor health not only requires significant expenditures on health-care and medical treatment, but may also drive affected persons into unemployment, poverty and social exclusion, if the environment does not accommodate their special needs.

The economic cost of output loss from NCDs, such as CVDs, chronic respiratory diseases, cancer, diabetes and mental health issues, has been estimated to be USD 47 trillion over the course of the next two decades (Bloom et al., 2011: 6). Approximately 70-80% of healthcare costs is spent on NCDs, which corresponds to EUR 700 billion in the European Union (EU) (Economist Intelligence Unit, 2012: 5).

NCDs are also closely linked to growing state expenditures on disability benefits. Persons with reduced work capacity due to health conditions can be recognized as disabled by their governments and thus be eligible for disability pensions. Public expenditure on disability benefits creates a significant financial pressure on countries. For example, in countries such as Denmark, Sweden and Norway, 3.8-4.4% of the GDP was spent on disability benefits in 2008; while in the EU-27 the figure was around 2% on average[6]. Difficult economic situation had eventually led to the need to reduce social protection expenditure in 2011 in most EU Member States (European Commission, 2015: 67). This includes the pressing need for reduction in benefits for the long-term unemployed, as well as reductions in expenditure on sickness and disability.

Furthermore, social benefits can lead to the risk of the benefit trap, making people with ill-health more dependent on passive income supports and discouraging them from entering the labour market. The reduction of the labour force, in turn, has a negative impact on the economic growth. According to a study commissioned by Randstad (Berkhout et al., 2012), there will be a potential shortfall of around 35 million workers, or about 15% of the total labour demand, by 2050. For this reason, it is important to ensure an inclusive labour market that would be able to meet the future labour demand and contribute to sustainable growth. Such inclusive markets can be made possible if every person of working age is given a possibility to participate in the open labour market and is provided with adequate support in doing so.

---

[6] Source: Eurostat, Social protection statistics http://ec.europa.eu/eurostat/statistics-explained/index.php/Social_protection_statistics_-_social_benefits_by_function

Participation of persons with chronic diseases in the open labour market can contribute to tackling the above-mentioned socio-economic challenges. As explained above, it has a potential to alleviate poverty and social exclusion, to encourage higher employment rates and labour supply, and to reduce public spending on disability benefits. Besides this, employment can have a positive impact on the well-being and mental health (OECD, 2010).

Consequences of health conditions may lead to a reduced work capacity and make it difficult for persons with NCDs to engage in work-related activities. In addition to health-related obstacles, there are also nonmedical factors that perpetuate long-term sick leaves and prevent persons with chronic conditions from returning to work, including personal, societal and work-related obstacles (Dekkers-Sánchez et al., 2010). Therefore, the types of support provided to persons with NCDs in returning to or staying in employment should not be limited to health-related rehabilitation only but should encompass environmental adaptations and accommodations.

Reduced unemployment, social equality and higher labour market participation are among the main priorities set by the EU's Europe 2020 strategy, in which the importance of participation of all working-age people regardless of their skill level in the labour market has been widely acknowledged (European Commission, 2010). To achieve inclusive and sustainable growth, everyone should be given an opportunity to enter and remain in the open labour market, including persons with NCDs. Hence, there is a need for implementing effective strategies to ensure their (re-)integration in the labour market.

The objective of this report is to map various strategies available at European level and at national level in ten countries: Austria (AT), Czech Republic (CZ), Germany (DE), Greece (EL), Italy (IT) Norway (NO), Poland (PL), Slovenia (SI), Spain (ES) and the United Kingdom (UK). The countries represent one of five European welfare models: Scandinavian, Continental, Anglo-Saxon, Mediterranean, and "Post-Communist" models. Identifying strategies in countries from different welfare models allows exploring potential commonalities and differences and identifying possible trends in the region. Strategies considered in this study include strategies at the level of policies, systems, and services. The mapping of professional (re-)integration strategies carried out in this report will lead in the later phases of the PATHWAYS project to the development of guidelines supporting the implementation of effective professional (re-)integration strategies for persons with NCDs.

The report is structured in the following way:

- **Part II** presents the definition of main concepts and explains the relationships between them. It also provides the descriptions of the seven categories of NCDs considered in this report as well as the descriptions of the five European welfare models.
- **Part III** is the review of European strategies. This part of the report presents (i) the statistical overview of the situation in Europe regarding the employment of persons with chronic diseases; (ii) the list of European policy frameworks concerning the employment of persons with NCDs; (iii) European funding programmes supporting the employment of persons with NCDs; and (iv) general policy tendencies in Europe.
- **Part IV** presents the overview of national strategies in ten countries representing five European welfare models. This part of the report also provides a comparison of strategies across countries.
- **Part V** concludes the report and provides relevant recommendations.

Methodology:

- The European strategies listed in Part III of the report have been identified through a desk research and a review of relevant statistical data and academic literature. The reviewed data sources included Eurostat, European Statistics of Income and Living Condition (EU-SILC), ANED, OECD and European Commission reports. In addition, a review of grey literature and articles from peer-reviewed journals has been carried out.
- National strategies presented in Part IV have been collected through questionnaires distributed by the partners of the PATHWAYS project in their respective countries. In addition, all partners conducted in-depth interviews with key stakeholders (service providers, users and public authorities). The lists of questionnaire and interview respondents can be found in Annex 1. The template of the questionnaire is in Annex 2 and the interview guidelines are provided in Annex 3.
- The findings of the report have been agreed upon during two focus groups involving the partners of the PATHWAYS project.

II. Analytical Framework

2.1. Definition of main concepts

Non-communicable diseases are defined by the WHO as diseases of long duration and generally slow progression that are not passed from person to person[7]. The terms non-communicable diseases and chronic diseases are used interchangeably in this report.

Persons with NCDs are defined in the framework of this report as persons with a long-standing chronic or mental health condition (or conditions) that cause a reduced capacity to participate in the open labour market[8]. Persons with NCDs can be employed, unemployed or inactive.

Persons with disabilities are persons who have “long-term physical, mental, intellectual or sensory impairments which in interaction with various barriers may hinder their full and effective participation in society on an equal basis with others”, according to Article 1 of the UN Convention on the Rights of Persons with Disabilities (UNCRPD). It should be noted that an illness or an injury alone does not constitute a disability unless it is accompanied by obstructive societal or environmental factors. Therefore, ill-health should be seen as a component of disability. It is important to note, however, that on a country level, governments may use their own definitions of disability, based on various factors such as the severity of health or working capacity impairment, in order to allocate disability benefits and supports. In such cases, persons with impairments caused by a NCD may or may not be assessed as disabled, depending on the laws of each country. Therefore, some persons with NCDs can be included in the group of persons with disabilities, while others are seen as non-disabled persons who receive no disability benefits, regardless of their needs for support. A distinction can be made between persons with NCDs whose health condition could qualify them for a disability status, but they do not want to be recognised as disabled for various reasons, and persons with NCDs whose health condition could qualify them for a disability status.

Professional (re-)integration strategies – policies, measures and services aimed at encouraging, facilitating and supporting persons with NCDs in joining or remaining in the open labour market.

---

[7] http://www.who.int/mediacentre/factsheets/fs355/en/

[8] For the purpose of the study we take a more narrow definition that is linked to employment. It should be noted, however, that in general, the definition of *persons with NCDs* is much broader and not limited to employment.

Policies – binding and non-binding legislative frameworks, provisions and policy approaches that set a course or a principle of action at international, regional, national or local level.

Systems – supports, programmes or schemes (including financial support) aimed at activating unemployed and inactive persons in obtaining or returning to work; the employed persons in remaining in work; and the employers and employment services in facilitating the participation of persons with chronic diseases in the labour market.

Services – services and activities by private or public entities aimed at assisting jobseekers in finding employment as well as social services that directly or indirectly contribute to the employability of persons with NCDs. In other words, services include both mainstream and specialised employment services.

2.2. Explaining relationships between concepts

The analytical framework of this report is schematically illustrated in Figure 1. It consists of the study *population*, of *interventions* directed at the population and of the *outcome* resulting from the interventions.

The target *population* of this report are persons with NCDs, including specifically the following seven disease categories: mental health issues, neurological diseases (with the focus on headache disorders), metabolic diseases, MSDs, respiratory diseases, CVDs and cancer. As mentioned earlier, some persons with NCDs can be recognised as persons with disabilities, depending on the legislation of individual countries, as shown in Figure 1. When this is the case, employment strategies for persons with disabilities are applicable to persons with NCDs, too. However, there are also persons with NCDs who, depending on their national regulations, do not fall under the category of persons with disabilities and are not covered by employment services, systems and policies that are in place for persons with disabilities. In order to produce a comprehensive map of European strategies helping persons with chronic conditions to obtain, retain and return to work, both of these groups of people should be considered.

The *interventions* are professional (re-)integration strategies. This study looks into three levels of strategies: policies, systems, and services. These three interventions are linked together: policies influence systems because systems are practically instruments allowing the achievement of policy

objectives. In turn, systems allow transforming policy goals into services and thus influence the types of services provided by public or private entities.

The *outcome* is the participation of persons with chronic diseases and mental health issues in the open labour market.

In short, the report explores policies, systems, and services that are targeted at persons with NCDs and/or persons with disabilities, and that aim to improve their participation in the open labour market.

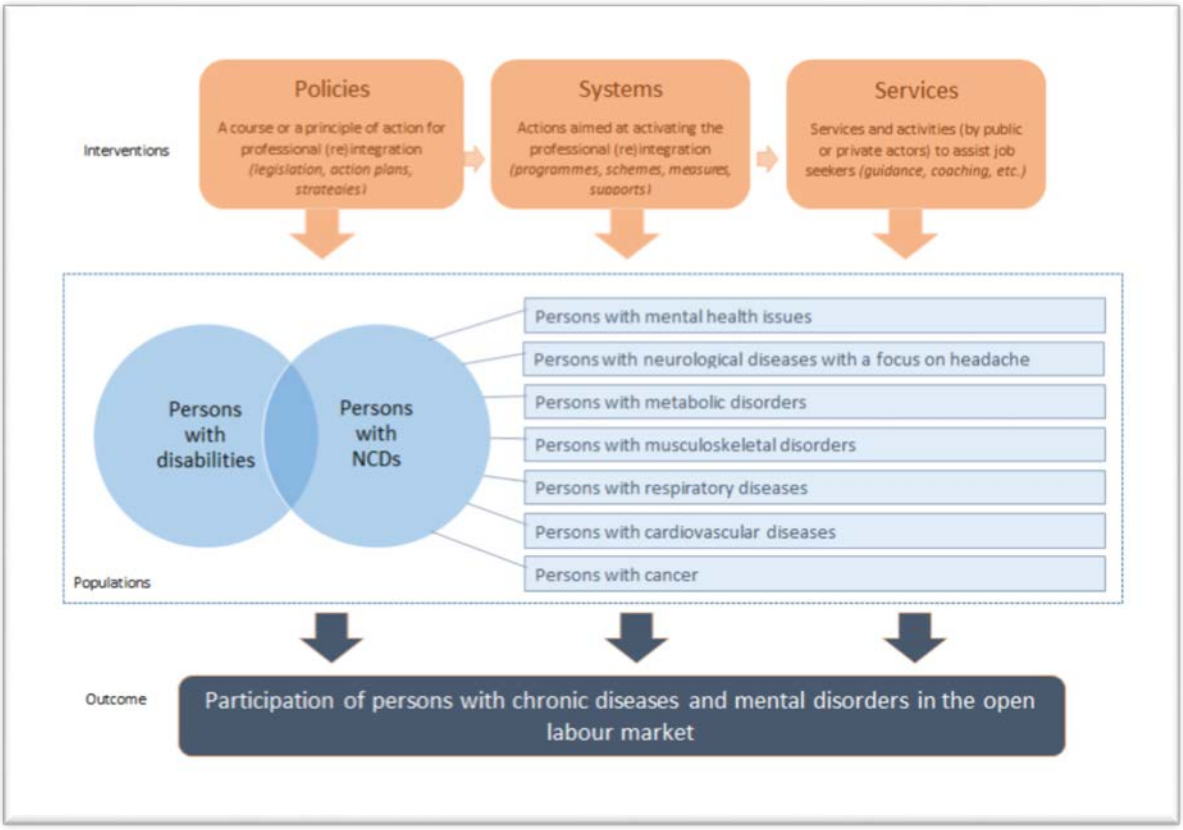

Figure 1. *Analytical Framework*

2.3. Description of categories of chronic diseases

For the purposes of this study, the categories of NCDs were selected based on their impact on labour market participation. WHO estimates of *years lost to disability (YLD)*[9] for years 2000 and 2012

[9] YLD is a WHO estimate of years lost due to disability for people living with a health condition or its consequences. To estimate YLD for a particular cause in a particular time period, the number of incident cases

provide a useful list of NCDs whose impact in the European region is rather significant. The WHO data on NCDs shows that 'mental and behavioural disorders' are responsible for most YLD in EU-28 plus Norway and Switzerland, followed by MSDs and neurological conditions (Figure 2).

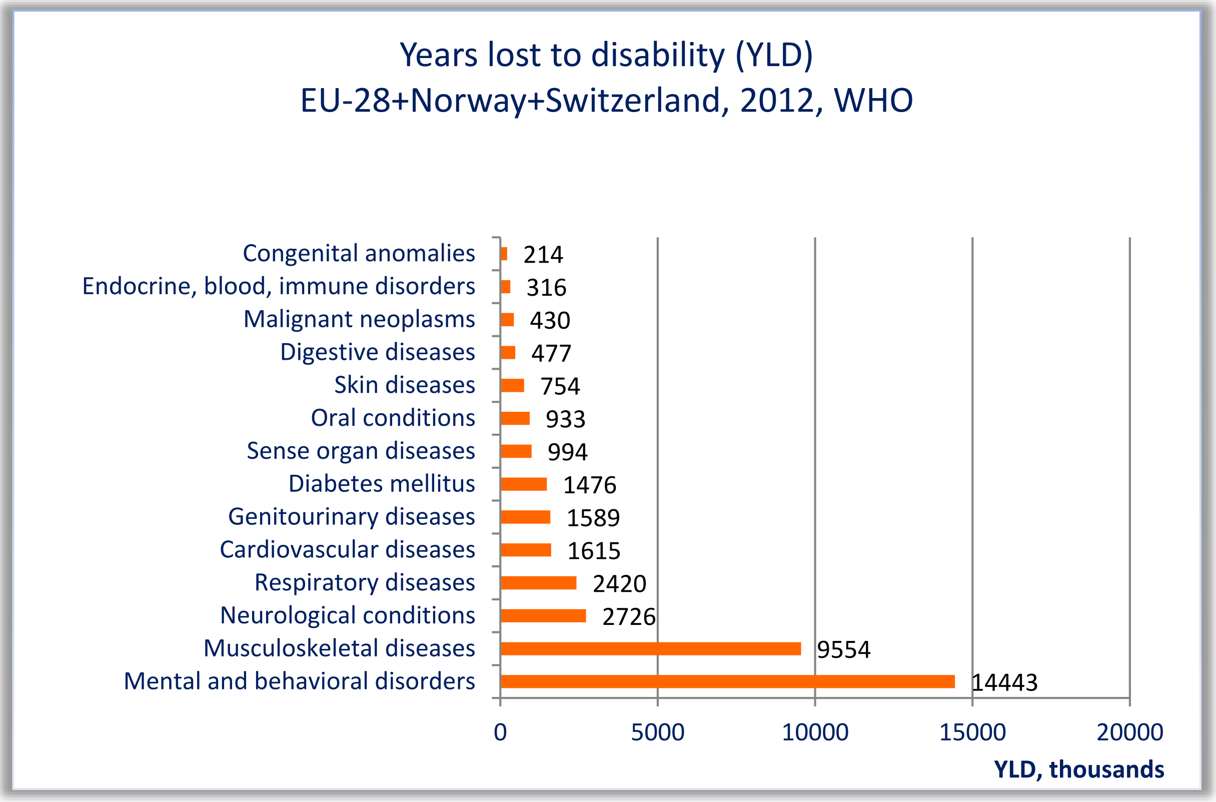

Figure 2. *YLD by cause (NCDs) for EU-28 plus Norway and Switzerland and percentage from all-cause YLD; total population aged 15-69. Source: WHO Global Health Estimates, 2012.*

Based on these estimates, the first five biggest contributors to years lost to disability are selected. In addition, it has been decided to include metabolic disorders (diabetes mellitus) and cancer (malignant neoplasms) to the list of NCDs for this study. Diabetes is widespread and cancer is a leading cause of death worldwide. Thus, these diseases certainly affect the ability to participate in the labour market.

Following is the description of all categories of NCDs considered in this report:

Mental health issues take various forms but are generally characterised by abnormal thoughts, perceptions, feelings, behaviour and relationships with others that produce either distress or impairment of function (WHO, 2013). Mental health conditions include depression, bipolar disorder,

in that period is multiplied by the average duration of the disease and a weight factor that reflects the severity of the disease on a scale from 0 (perfect health) to 1 (dead).

schizophrenia, anxiety disorder, etc. The burden of mental health problems is continuously rising, affecting social, human rights and economic aspects of the society (Kessler and Ustun, 2008).

Within the WHO category of 'mental and behavioural disorders', unipolar depressive disorders are the biggest contributors to YLD, being responsible for 10% of all years of life with a health condition or its consequences (5.2 million YLD for EU-28 plus Norway and Switzerland, age range 15-69) in 2012, according to WHO estimates. Depression is a common mental condition that affects millions of people of all ages globally. It alone accounts for 4.3% of the global burden of disease and is one of the largest single causes of disability in the world (WHO, 2013: 8). In the Labour Force Survey (LFS) 2007 ad hoc module, about 14% of the respondents with work-related health problems identified stress, depression or anxiety as the most serious health problem (Eurostat, 2010: 67). The costs of mental health problems to the economy are estimated at 3-4% of GDP (Corral et al., 2014: 3). Mental health problems are considered to be the biggest single cause for disability benefits (nearly half of all new claims) in a number of countries, including Denmark, the Netherlands, Sweden and Switzerland (OECD, 2010: 11). Disability policies are in general considered inadequate and inefficient in dealing with mental health problems as they do not work well for people with mental health conditions (OECD, 2010: 11).

Musculoskeletal disorders (MSDs) are related to injuries and disorders that affect the human body's movement or musculoskeletal system, i.e. muscles, tendons, the skeleton, ligaments and nerves (Luttmann et al, 2003). MSDs include back and neck pain, rheumatoid arthritis, osteoarthritis and others. The symptoms may range from discomfort and pain to decreased body function and disabling injuries. The impact of MSDs on working life is significant since these disorders can cause reduced productivity, increased sickness absence, a high proportion of days lost, and chronic occupational disability (Schneider and Irastorza, 2010). In Europe, chronic musculoskeletal pain affects 100 million people and is widespread among working age population, despite being undiagnosed in over 40% of cases (Bevan et al., 2009). According to a 2005 European survey, up to 25% of workers in the EU-27 reported back pain, and 23% muscular pain related to work (European Agency for Safety and Health at Work (EU-OSHA), 2007: 12). Differences between countries are huge. For example, in Greece, 47% of workers reported work-related back pain and 46% muscular pain, while in the UK the respective figures were 11% and 9% (ibid.). According to the LFS 2007 ad hoc module, about 60% of the respondents with work-related health problems identified musculoskeletal problems as their most serious work-related health problem (Eurostat, 2010: 9). In employed persons, limitations to carry out daily work tasks were reported relatively often in those with musculoskeletal problems of the hips, legs or feet (some limitations 54% and considerable

limitations 19%) and in those with back problems (some limitations 56% and considerable limitations 15%) (Eurostat, 2010: 51). Taking the high incidence of musculoskeletal problems into account, about 60% of all short term and long term sickness absences in the EU-27 can be attributed to musculoskeletal problems (Eurostat, 2010: 63). According to estimates, up to 2% of the European GDP is accounted for by the direct costs of MSDs each year (Zheltoukhova, 2013: 10).

Within the category of MSDs, back and neck pain accounts for the largest number of YLD, specifically, 5.3 million YLD according to WHO estimates for 2012 in EU-28 plus Norway and Switzerland, age range from 15 to 69.

Neurological disorders are diseases of the central and peripheral nervous system, i.e. the brain, spinal cord, cranial nerves, peripheral nerves, nerve roots, etc. (Dua et al., 2006). These disorders include epilepsy, Alzheimer disease, migraine and other headache disorders, multiple sclerosis, etc. They are an important cause of mortality and responsible for 12% of total deaths worldwide (Dua et al., 2006: 35). According to a 2010 report on tackling chronic disease, neuropsychiatric conditions are the second most common chronic disease in Europe after CVDs (20% of all cases) (Busse et al., 2010).

Within the category of neurological disorders among person aged 15-69, migraine has the largest impact - 1.5 million YLD. Headache disorders, including migraine, are among the most common disorders of the nervous system. Headache disorders are often life-long conditions that have been largely underestimated, under-recognized and under-treated worldwide (WHO, 2011: 11). About 50-75% of all adults aged 18–65 years have had headache in the last year and more than 10% have reported migraine (WHO, 2011: 25). Unlike other chronic diseases, headache disorders are not associated with significant mortality rates; however, they do impose a considerable burden on affected persons through pain, personal suffering, impaired quality of life as well as inability to participate in social life and employment. Headache disorders have a disabling nature: nearly every person with migraine and 60% of those with tension-type headache are subjected to reductions in social activities and work capacity (Leonardi et al, 2005). Recurrent headache attacks, and often the constant fear of the next attack, affect the social and family life, and undermine career and financial opportunities (WHO, 2011).

Chronic respiratory diseases are chronic diseases of the airways and other structures of the lung (WHO, 2016). The most common chronic respiratory diseases include asthma, chronic obstructive pulmonary disease (COPD), occupational lung diseases and pulmonary hypertension. Within the category of respiratory diseases, COPD is the main cause of disability, accounting for 1.3 million YLD

in 2012 in EU-28 plus Norway and Switzerland for the age group 15-69. COPD is a disease of lungs that obstructs normal breathing and can be life-threatening. The primary cause of COPD is tobacco smoke. COPD is not curable, but treatment can slow the progress of the disease. The burden of respiratory diseases including COPD and asthma is estimated at EUR 380 billion each year, including costs of healthcare and lost work days (Corral et al., 2014: 3).

Cardiovascular diseases (CVDs) are disorders of the heart and blood vessels that cover various circulatory system diseases ranging from ischemic heart disease to cerebro-vascular disease (WHO, 2015). Within the category of CVDs, ischaemic heart disease is the biggest contributor to YLD, accounting for 717.3 thousand YLD in 2012 in EU-28 plus Norway and Switzerland (age group 15-69), according to WHO estimates. Ischemic heart disease is a common CVD caused by the accumulation of fatty deposits lining the inner wall of a coronary artery, restricting blood flow to the heart. It alone was responsible for around 18% of all deaths in EU Member states in 2011 (OECD, 2014: 22).

Metabolic disorders are represented by diabetes mellitus, a common metabolic disorder associated with hyperglycemia resulting from defects in insulin secretion, insulin action, or both (American Diabetes Association, 2004). In the EU, about 32 million adults aged 20-79 years old had diabetes in 2013, which is equivalent to 6% of the population in this age group (OECD, 2014: 42). For the same year, health expenditure on prevention and treatment of diabetes and its complications was estimated at USD 147 billion in Europe (International Diabetes Federation, 2013: 58). Diabetes was responsible for 1.5 million YLD in 2012 in EU-28 plus Norway and Switzerland among people aged 15-69, according to WHO estimates.

Cancer is a generic term for a large group of diseases that can affect any part of the body. It is characterised by a rapid creation of abnormal cells that grow beyond their usual boundaries, and which can then invade adjoining parts of the body and spread to other organs (WHO, 2002). In the EU, cancer was the second leading cause of death, after CVDs, accounting for 26% of all deaths in EU countries in 2011, with lung, colon and prostate cancer being the main causes of cancer death among men and breast, colon and lung cancer among women (OECD, 2014: 20). Mortality rates differ across EU Member states, with the lowest rates being found in Cyprus, Finland, Bulgaria, Sweden and Switzerland, and the highest in some Central and Eastern European (CEE) countries such as Hungary, Croatia, the Slovak Republic, Slovenia and Denmark (OECD, 2014: 24). Cancer survivors have a 37% higher risk of unemployment and a threefold risk of disability compared to people without cancer (Taskila et al., 2013: 4). Within this category, breast cancer accounts for the highest

number of YLD among people aged 15-69 in EU-28 plus Norway and Switzerland in 2012 (118.8 thousand YLD), according to WHO.

2.4. Description of European welfare models

The report analyses the following five European welfare models:

1) Scandinavian (Nordic) model;
2) Continental (Bismarckian) model;
3) Anglo-Saxon model;
4) Mediterranean (Southern) model;
5) "Post-Communist" model.

The description of main features of these models is summarised in the table below[10]:

Table 1. *Features of European welfare models.*

Model name	Features	Country examples
**Scandinavian model**	• Emphasis on egalitarianism and universal welfare provision (Popova & Kozhevnikova, 2013);<br>• Universal and generous benefits and a strong redistributive social security system (Eikemo et al., 2008b and Fenger, 2007);<br>• Extensive fiscal intervention through the use of active labour market policies, strong employment orientation (Midttun, 2006).	NO
**Continental model**	• Benefits tied to employment, financed mainly by employer and employee (Eikemo et al., 2008b);<br>• Minimal redistribution (ibid)<br>• Social security is organized as insurance system (Caritas, 2012)	AT, DE, SI
**Anglo-Saxon model**	• Relatively large social assistance of the last resort (Midttun, 2006);<br>• Cash transfers are mainly oriented to people in working age (Midttun, 2006);<br>• Schemes conditioning access to benefits to regular employment and emphasis on activation measures (Midttun, 2006);<br>• A low level of government spending on social protection, modest benefits, usually means-tested (Eikemo et al., 2008a and Fenger, 2007);<br>• Little redistribution of incomes (Fenger, 2007)<br>• High incidence of low-pay employment (Midttun, 2006).	UK

[10] See more here http://www.easpd.eu/sites/default/files/sites/default/files/SensAge/d4-social_welfare_systems_across_europe.pdf

**Mediterranean model**	• A dualist system of welfare provision, which strongly protects part of the population while under-protecting another (Campos-Matos and Kawachi, 2015);<br>• High segmentation of entitlements and conditioned access to social provisions (European Association of Service providers to Persons with Disabilities (EASPD), 2014);<br>• Welfare and social policies in fighting poverty are ineffective and fragmented (Caritas, 2012);<br>• Less generous benefits in comparison to the Continental model and not all the branches of social insurance are equally developed (Caritas, 2012);<br>• High dependence on informal, charitable and family care (Eikemo et al., 2008a).	EL, ES, IT
**"Post-Communist" model**	• Generally low governmental spending on social programs, mostly financed through social contributions (Campos-Matos and Kawachi, 2015)<br>• Relatively limited health service provision and poor overall population health system (Eikemo et al., 2008a).<br>• On-going transition process from institutional to community-based care (EASPD, 2014);<br>• Insufficient implementation and monitoring of the developed legislation, plans and strategies concerning the wellbeing of persons with disabilities (EASPD, 2014);<br>• Lower levels of governmental programmes and the social situation (Fenger, 2007);<br>• Generally incoherent legal framework.	CZ, PL

An important difference between the welfare models lies in the orientation of their disability policies. Specifically, it is useful to know the extent to which states move away from merely paying disability benefits towards actually providing incentives to staying in work. The OECD (2010) published a report on the direction of disability policy reforms in its member states and ranked the countries based on their policy orientation. The report made a distinction between compensation-oriented policy and activation/integration policy. The report found that Nordic states apply the social-democratic model that is strong in both types of policy: it provides generous and universal disability compensation while at the same time offering extensive employment and vocational rehabilitation programmes. The UK, on the other hand, follows the liberal model: it is stricter on disability compensation but provides well organised and co-ordinated employment activation services. Most Continental EU states fall under the corporatist model: while having well-developed integration policies, they still provide generous sickness and disability benefits, which makes them more compensation policy-oriented. The Mediterranean states are also classified as having the corporatist model, but in comparison to the Continental states, they have comparatively underdeveloped employment and rehabilitation policies as well as lower sickness benefits. The

OECD report does not provide ranking for most countries representing the “post-communist” model, with the exception of Hungary, Poland, Czech Republic and Slovakia, which are characterised by a greater compensation policy orientation. It is, however, generally known that CEE countries have relatively tighter budgets allocated to social and healthcare policies and therefore provide lower levels of benefits and have underdeveloped activation programmes.

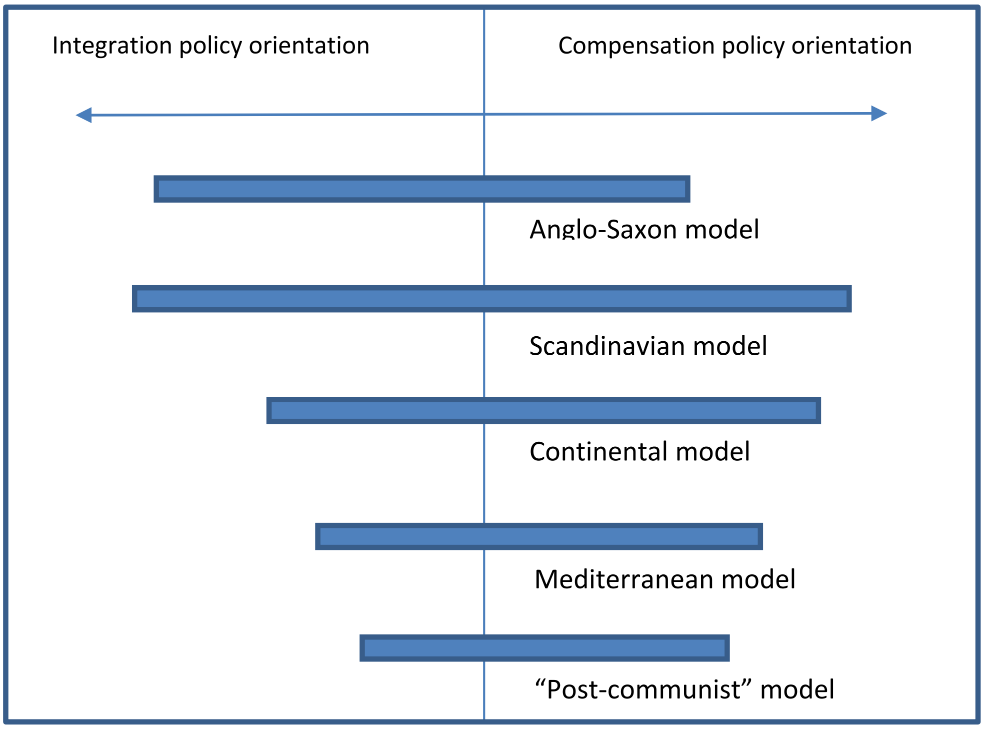

Figure 3. *Relative policy orientation of five European welfare models.*

Based on this ranking it is possible to build a scheme, illustrated in Figure 3, which allows visualising the differences between welfare models. The scheme does not provide precise absolute values of the degree of policy orientation, but it is still a useful tool that allows understanding differences among models in relative terms. The figure shows that the Scandinavian model has a more balanced mix of compensation and integration policies than other models. Furthermore, in this model, both policy orientations are stronger and more developed than in other models. The Anglo-Saxon model is more tilted towards integration policies, but these policies are still slightly less developed than in Nordic countries. The rest of the models are tilted towards compensation policies, but their compensation policies are not as strong as in Scandinavian countries. It is also interesting to see that compensations policies in the Anglo-Saxon model are weaker than in “Post-communist” countries.

III. European strategies

3.1. Chronic diseases and employment: European state of play in numbers

Statistical data on the prevalence of chronic diseases in Europe as well as the information on their mortality can be found in Annex 4.

Employment of persons with chronic diseases

According to a 2014 Eurostat report, which was based on the data of the 2011 ad hoc module of the EU LFS, the employment rate for people limited in work due to a longstanding health problem and/or a basic activity difficulty (LHPAD) is 38.7% (Figure 4). Higher employment rates can be observed in France, Sweden and Switzerland (above 60%), while lower rates are found Bulgaria, Hungary and Ireland (18-22%).

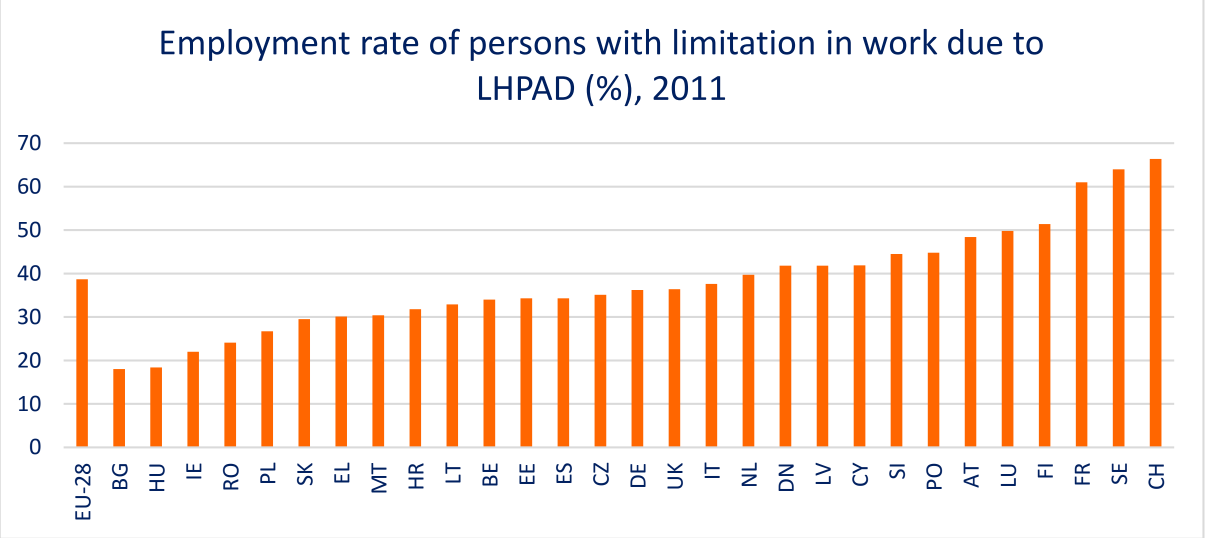

***Figure 4.** Employment rates of persons with limitations in work due to LHPAD, total population of 20-64 years of age. Source: Eurostat, 2011, (hlth_dlm010).*

Across all countries, there is a considerable gap between employment rates of persons with and without health related limitations in work. For EU-28, for example, the employment rate for persons with limitations is 34.2 percentage points less than for people who did not declare a limitation in work[11]. The situation is fairly similar in all EU Member states. Some countries such as Ireland, Romania, Hungary and Bulgaria have reported a large gap (over 45 percentage points) between

[11] Source: Eurostat, hlth_dlm010 2011, both sexes, age group of 20-26 years.

people with and without work limitations (Figure 5). Meanwhile, France reported the lowest gap, of 13.7 percentage points.

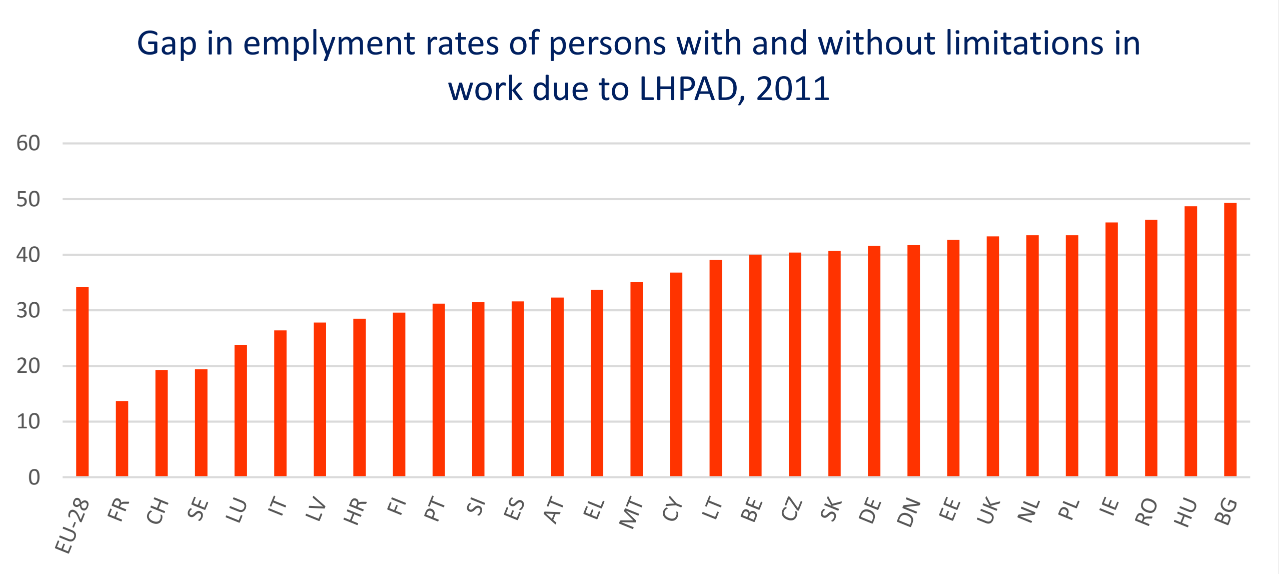

Figure 5. *Difference in employment rates (ER) between persons with no limitations in work due to LHPAD (PWNL) and persons with limitations in work due to LHPAD (PWL) per country (ER_{PNLW} - ER_{PWL}), total population of 20-64 years of age. Source: Eurostat, 2011, (hlth_dlm010).*

Employment rate varies across different categories of diseases. It appears that among people who reported LHPAD-related limitations in work, the employment rate is particularly low among persons with psychosocial problems[12], with only 23.6% of them being employed, which is lower than the rates for other health conditions (Figure 6). For CVDs[13], cancer and diabetes, the employment rate is also low - less than 30%. Relatively higher employment rates can be observed among people with MSDs[14] and neurological diseases. It should be noted, however, that the category of neurological diseases in this data set includes sub-categories such as epilepsy and severe headaches (e.g. migraine), excluding other severe diseases such as Alzheimer's disease and other dementias, which presumably lead to lower employment rates[15]. While the employment rate for persons with epilepsy is low (24%), it is much higher for persons with severe headaches (56.4%). In fact, the severe headaches is one of two chronic disease sub-categories for which the number of employed persons exceeds the number of those who are not employed. The other sub-category is skin conditions including allergic reactions and severe disfigurement, which has an employment rate of 51.6%. The

[12] Category includes chronic anxiety, depression and other mental, nervous or emotional problems.
[13] This category includes heart, blood pressure or circulation problems.
[14] This category includes problems with arms or hands; legs and feet; and back and neck (including arthritis or rheumatism).
[15]Diseases such as multiple sclerosis, Alzheimer's disease and Parkinson's disease are grouped in a sub-category called “Other progressive illnesses (which include multiple sclerosis, HIV, Alzheimer's disease, Parkinson's disease)”. The combined employment rate for this sub-category is 30.5%.

lowest employment rate, of 23.6%, is reported for persons with depression (See employment rates for chronic disease sub-categories in Annex 5).

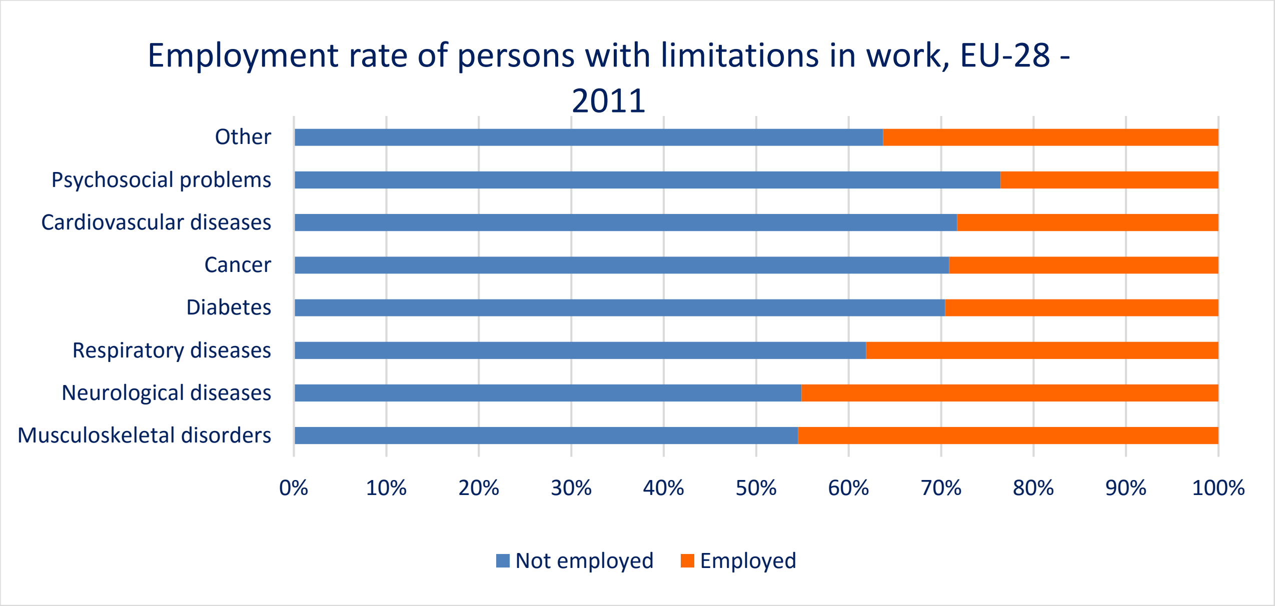

Figure 6. *Rate of employment per chronic disease category for persons with limitations in work due to LHPAD; both sexes, age group 20-64 years. Source: Eurostat, LFS ad hoc module 2011.*

According to the Eurostat (2010: 53) report on health and safety at work in Europe (1999-2007), prolonged sickness absence of one month or more is frequent among employed persons with circulatory system problems, including heart disease or attack (29%); stress, depression or anxiety (25%) and musculoskeletal problems (25%).

In general, the employment rate among persons with LHPAD is low, which suggests that they are less likely to engage in full-time employment and more likely to be unemployed or inactive. In 2011, the unemployment rate for people limited in work because of LHPAD was 17.4 % in 28 EU states, which is 8 percentage points higher than the unemployment rate for those without health problems or activity difficulties (9.4 %)[16]. Countries that reported the biggest gap, of over 14 percentage points, include Hungary, the Czech Republic, Germany and Estonia. Meanwhile, in Turkey and Greece, the unemployment rate for people without any activity difficulty was actually higher than for those with difficulties and limitations[17]. It is important to note, however, that in relation to Greece, the figures can be misleading since the inactivity rate for persons with limitations in work due to LHPAD reached 65%.

[16] Source: Eurostat, 2011 LFS ad hoc module (hlth_dlm030)
[17] ibid.

In 22 EU countries the inactivity rates for persons with LHPAD exceed 50%. This means that in these countries more than half of the working age population with limitations in work are not economically active and do not participate in the labour market[18].

Overall, in terms of employment, people with chronic diseases have a higher risk of unemployment and inactivity as they experience relatively quick transition paths from employment to unemployment/inactivity and rather complicated transition paths from unemployment/inactivity to employment (Corral et al., 2014).

Types of employment

Based on 2011 data, 13% of persons with disabilities in the EU work part-time and 34% work full-time, while for persons without disabilities the rates are 13% and 58% respectively (ANED, 2013: 34). Statistics also show that the more severe is the disability, the more likely it is for an employee to hold a part time job. It may be also concluded that a high share of part-time work is a voluntary choice, especially among persons with a severe disability (ANED, 2013: 36). The patterns of part time job vary across countries: in Poland and Sweden, for example, people with a disability are three times more likely to take up a part-time job, while in Austria, Germany, Italy and Spain the difference between people with and without a disability is minor (OECD, 2010: 51). Furthermore, the share of people working from home was higher among people with a disability (15%) than others (13 %) in most EU countries except Belgium, Denmark, Germany, France, Slovenia and Slovakia.

Evidence also shows that persons with chronic diseases are likely to be employed in lower level manual occupations that require lower skill levels (Paauw, 2015). Sectors that have higher presence of persons with chronic diseases are agricultural/fishing/forestry and industrial sectors (Corral et al., 2014).

Social protection measures and types of assistance

The World Social Protection Report 2014-2015 highlights the fact that many countries have an insufficient coverage of the needs of persons with disabilities in their social protection programmes and that, where provisions are made, these often do not support their participation in the labour market (ILO, 2014).

[18] Source: Eurostat, 2011 LFS ad hoc module (hlth_dlm040)

Furthermore, statistics show that in the EU, 8.5 % of employed people who had a LHPAD used some kind of assistance to get the job.[19] The Netherlands reported the highest figure, 33%, which implies that employers in this country are doing more to assist persons with disabilities who are in employment; while the lowest figure of 4% was reported in France[20]. At the same time, 28% of not employed persons with a LHPAD in the EU reported that they would need assistance in order to work[21]. In countries like Romania, Slovakia and Slovenia the figure exceeded 50%, while in France, the figure was the lowest – 8%.[22]

The research considered three kinds of assistance:

- Personal assistance, which includes assistance from colleagues, friends or relatives;
- Special equipment or workplace adaptations, including physical alterations to buildings;
- Special working arrangements, such as sedentary work, teleworking or flexible hours.

Within the EU, *special working arrangements* was the most used and the most needed type of assistance among persons with LHPAD: a total of 5.2% of employed persons with LHPAD reported using special arrangements and 24.2% of non-employed persons with LHPAD reported that they would need such help to be able to work[23]. Similarly, *personal assistance* and *special equipment* were used by 2.3% and 3.3% of working persons with LHPAD respectively, and reported as needed by 13% and 12.2% of non-employed persons with LHPAD respectively[24]. Interestingly, for all three types of assistance, the number of persons who said they needed assistance significantly exceeded those who had actually used it. On the national level, the figures for the use of all three types of services were among the highest in the Netherlands.

Another European study has identified that the supply of support in terms of adaptations of workplace and work content does not meet the needs of persons with chronic diseases (Corral et al., 2014). In other words, not everybody with support needs is actually provided with such assistance. For example, in Belgium, 53% of workers with chronic diseases requested an adaptation of tasks, but only 34% of them actually obtained the support; in the Czech Republic 27.6% reported work adjustment needs but only 11.4% received support (ibid). In general, larger enterprises appear to be

[19] Source: Eurostat, 2011 LFS ad hoc module (hlth_dlm180)
[20] Ibid.
[21] Source: Eurostat, 2011 LFS ad hoc module (hlth_dlm190)
[22] Ibid.
[23] Ibid.
[24] Ibid.

performing better in providing reasonable accommodation and having in place employee retention and integration policies (ibid.)

Barriers to employment

In 2011, about 19% of all people aged 15-64 years in EU-28 had some limitation at work, with 38% of them naming a LHPAD as the main reason[25]. The difference in terms of personal and environmental barriers in work is quite significant between persons with and without LHPAD: over 35% persons with LHPAD face such barriers, while the rate for persons without LHPAD is 9%[26].

The state of health evidently plays an important role in labour market participation. The main reason for leaving the last job was "own illness or disability" for over 35% of not employed persons with limitations in work caused by a LHPAD in EU-28.[27] Nevertheless, apart from health problems, people with a reduced work capacity also face environmental obstacles in employment. For example, a qualitative study into perpetuating factors for long-term sick leaves in the Netherlands has found that there are various nonmedical factors besides sickness that prevent persons with chronic conditions from returning to work (Dekkers-Sánchez et al., 2010). Such factors include older age, lack of vocational rehabilitation counselling, and lack of cooperation from employers in modifying working conditions (ibid.). In contrast, factors such as a better control over working conditions, personal guidance and support from health authorities and health professionals, and a positive attitude of the persons have been among factors that facilitate the return to work (ibid.).

According to research from a number of European countries such as the Czech Republic, the UK, Ireland and Slovakia, persons with chronic conditions are particularly affected by discrimination, prejudice and even bullying and harassment at work, in most cases due to their health problems (Corral et al., 2014).

There is also evidence that employees with chronic diseases are more limited in their capacity to insist on adjusted workload, length of the working day and possibility to work from home (ibid.).

Statistics for Europe clearly show that NCDs pose a serious problem to society by negatively affecting labour market participation. The developmental risks associated with chronic diseases require high level policy intervention. The next section of the report provides an overview of European policy

[25] Source: Eurostat 2011 LFS ad hoc module (hlth_dlm140)
[26] Ibid.
[27] Source: Eurostat 2011 LFS ad hoc module (hlth_dlm070), age group 15-64, both sexes, missing answers not included in calculation the percentage.

frameworks that were put in place to promote the participation of persons with NCD in the labour market.

For more detailed descriptive statistics on each category of chronic diseases please see the Annex 6.

3.2. European policy frameworks on the employment of persons with chronic diseases

In order to have a more comprehensive overview on European policies on the inclusion of persons with ill-health in the labour market, it is important to consider a wide range of policies areas, including policies on the rights of persons with disabilities, inclusion and anti-discrimination, and employment. Although these policies do not necessarily specifically address chronic illnesses, they do provide overarching frameworks that may promote work (re-)integration policies for persons with chronic diseases. The following two sections take a closer look at frameworks shaped by European institutions with an objective to improve the employment of persons with chronic diseases and mental health issues. In addition, policies with regard to specific disease categories are also considered.

It should be noted that this study considers only those policies, measures and services that deal with employment. Thus, the study does not consider policies focusing solely on the health aspects of NCD prevention and control.

A full overview of European policies is not possible without considering international policies, which by all means highly influence EU-level frameworks. The overview of international policy frameworks on the employment of persons with NCDs and disabilities can be found in Annex 7.

EU frameworks:

EU Directive on Employment Equality 2000/78/EC[28]. The directive was adopted in 2000 and prohibits discrimination on various grounds including disability in employment. It was transposed by all EU Member States. Article 5 of the Directive holds that reasonable accommodation should be provided for persons with disabilities to enable them "to have access to, participate in, or advance in

[28] Available at http://eur-lex.europa.eu/legal-content/EN/TXT/PDF/?uri=CELEX:32000L0078&from=EN

employment, or to undergo training, unless such measures would impose a disproportionate burden on the employer" (Council of the European Union, 2000: 21).

Equal opportunities for people with disabilities: a European action plan (2004-2010)[29]. Launched in 2003, the action plan aimed to mainstream in relevant Community policies and foster the accessibility of the labour market.

Disability Action Plan 2006-2015[30]. The action plan was adopted by the Committee of Ministers of the Council of Europe on April 5, 2006 and aimed to guide Member States in reinforcing anti-discriminatory and human rights measures to ensure equal opportunities and independence of people with disabilities in various aspects of life, including employment. The promotion, implementation and follow-up of the Plan are overseen by the European Coordination Forum for the Council of Europe Disability Action Plan 2006-2015 (CAHPAH).

Community strategy 2007-2012 on health and safety at work[31]. The European Commission's strategy specifically highlighted the importance for Member States to incorporate measures such as financial assistance, and training tailored to individual needs into national strategies in order to improve the rehabilitation and reintegration of workers excluded from the workplace for a long period of time. The European Parliament has welcomed the initiative and stated that it "applauds the special focus on rehabilitation and reintegration called for in the national strategies," and stressed the importance of ensuring "job retention (through training, reallocation of tasks, etc.) for people who have experienced physical or mental illness during their working lives" (European Parliament, 2008).

EU Strategic Framework on Health and Safety at Work 2014-2020[32]. The document seeks to ensure that the EU continues to play a leading role in the promotion of high standards for working conditions. This framework stresses the need of reintegration and rehabilitation measures allowing for early return to work after an accident or disease in order to avoid the permanent exclusion of employees from the labour market. The strategy also addresses the issue of ageing of the workforce and called for appropriate adaptation of workplaces and work organisation, including working time, workplace accessibility and workplace interventions targeted at older workers. Member States were encouraged to use European Social Fund (ESF) and other European Structural and Investment Funds to finance actions relating to occupational health and safety. The document specifically mentions

---

[29] Available at http://eur-lex.europa.eu/legal-content/EN/TXT/HTML/?uri=URISERV:c11414&from=EN
[30] http://www.coe.int/t/e/social_cohesion/soc-sp/Rec_2006_5%20Disability%20Action%20Plan.pdf
[31] Available at http://eur-lex.europa.eu/LexUriServ/LexUriServ.do?uri=COM:2007:0062:FIN:en:PDF
[32] Available at http://eur-lex.europa.eu/legal-content/EN/TXT/PDF/?uri=CELEX:52014DC0332&from=EN

supports in recruitment and return to work of people with a chronic or rare disease, disability or mental issues, and the use of integrated employment measures such as individualised support, counselling, guidance, access to general and vocational education and training, and other.

European Commission White Paper "Together for Health: A strategic approach for the EU 2008-2013."[33] The document was released in 2007 and became the first attempt to have a coherent EU-wide long-term health strategy. The document formulated main principles for EU action on health and specifically highlighted the importance of person-centred action that would allow patients to become active actors and decision makers in issues concerning their health. Furthermore, it called for reducing the number of people inactive due to ill-health and acknowledged that fact that ill-health has enormous direct and indirect costs to both the society and the economy. It also encouraged the mainstreaming of the Health in All Policies principle.

Commission Recommendation 2008/867/EC on the active inclusion of people excluded from the labour market[34]. The recommendation, adopted on October 3, 2008, calls Member States to adopt arrangements covering persons whose condition renders them fit for work to ensure they receive effective help to enter or re-enter and stay in employment that corresponds to their work capacity. The document highlights the importance of promoting following principles: addressing the needs of people excluded from the labour market to facilitate progressive reintegration into the labour market and to enhance their employability; promoting inclusive labour markets to ensure equal access to employment; promoting quality jobs, pay and benefits, working conditions, health and safety, access to lifelong learning and career prospects, in particular with a view to preventing in-work poverty; and tackling labour market segmentation by encouraging job retention and advancement.

European Disability Strategy 2010-2020[35]***.*** In 2010, the European Commission adopted the European Disability Strategy 2010-2020, which builds upon the UNCRPD and the Disability Action Plan (2004-2010). One of a total of eight priority areas highlighted by the strategy is the employment of persons with disabilities. Its objective is to increase the share of persons with disabilities working in the open labour market.

---

[33] Available at http://ec.europa.eu/health/ph_overview/Documents/strategy_wp_en.pdf
[34] Available at http://eur-lex.europa.eu/legal-content/EN/TXT/PDF/?uri=CELEX:32008H0867&from=EN
[35] Available at http://eur-lex.europa.eu/legal-content/EN/TXT/HTML/?uri=URISERV:em0047&from=EN

Europe 2020: the European Union strategy for growth and employment.[36] The strategic objectives of the Together for Health strategy have been taken into account in formulating the EU strategy for smart, sustainable and inclusive growth, Europe 2020. In particular, Europe 2020 highlights the importance of public health in fighting poverty and in improving productivity, workforce participation and competitiveness. Some of the important health-related initiatives of the strategy include: the *European Innovation Partnership on Active and Healthy Ageing*, which attempts to improve the health and quality of life of older people; the *Agenda for new skills and jobs*, which calls for adopting targeted approaches for the more vulnerable workers, including older workers, disabled people and people with mental health issues, in providing career guidance and adapted training and work experience programmes; and the *European Platform against Poverty*, which aims to reduce health inequality. These health-related initiatives are supposed by EU Health programmes, which serve as instruments in implementing the EU health strategy and are discussed later in this report.

Reflection Process on Chronic diseases: Final Report[37]***.*** In December 2010, the Council of the European Union invited the EU Member States and the Commission to initiate a reflection process on chronic diseases and to coordinate efforts to respond to the challenges of chronic diseases. By 2013, the Commission conducted consultations with major stakeholders in Member States and released the final report on October 8, 2013. The report highlighted, among other things, the importance of patients' empowerment, ability to make independent decisions and active participation in all aspects of society.

Council Recommendation on the integration of the long-term unemployed into the labour market[38]***.*** In September 2015, the European Commission proposed a new initiative on long-term unemployment by providing guidance to Member States to better help long-term unemployed return to work. The initiative envisages that jobseekers who have been unemployed for more than 12 months receive an individual assessment and a job integration agreement, which offers a concrete and personalised plan to bring the jobseekers back to work. Furthermore, the proposal calls for the involvement of employers through partnerships with the public authorities and foresees targeted financial incentives and other services for the employers. The implementations of these recommendations can be supported by the ESF. This policy is relevant to our study since long-term unemployment is often associated with chronic diseases. A study by Pohjola (2001) shows strong

---

[36] Available at http://ec.europa.eu/eu2020/pdf/COMPLET%20EN%20BARROSO%20%20%20007%20-%20Europe%202020%20-%20EN%20version.pdf
[37] Available at http://ec.europa.eu/health/major_chronic_diseases/docs/reflection_process_cd_final_report_en.pdf
[38] Available at http://ec.europa.eu/social/BlobServlet?docId=14480&langId=en

evidence indicating that health problems are one distinct risk factor for the long-term unemployed and in finding a job. A study by Schuring et al. (2007) investigated the impact of poor health on selection for paid jobs in European countries and found that in most countries, ill health or a chronic health problem predicted staying or becoming unemployed. The study suggests that ill health increases the likelihood of early retirement and highlights the Importance of preventive public health measures aimed at reducing labour force exit for persons with ill health. This means that persons with chronic diseases face an imminent risk of long-term unemployment.

European Accessibility Act[39]. In December 2015, European Commission launched the "Proposal for a Directive of the European Parliament and of the Council on the approximation of the laws, regulations and administrative provisions of the Member States as regards the accessibility requirements for products and services". The proposal aims to set common accessibility requirements for ICT products and mobile services to support the implementation of Article 9 of the UNCRPD.

Joint Action on Chronic Diseases (JA-CHRODIS)[40] is a three-year initiative (2014-2017) co-financed under the EU Public Health Program. It brings together over 60 associated and collaborating partners from health and research institutions in 26 Member States. The collaboration aims to identify, validate, exchange and disseminate good practice on chronic diseases across EU Member States and to facilitate its uptake across local, regional and national borders. The focus is health promotion and primary prevention as well as the management of diabetes and multi-morbid chronic conditions. One of the key deliverables is a 'Platform for Knowledge Exchange', which will include both an online help-desk for policy makers and a clearinghouse providing an up to date repository of best practices and the best knowledge on chronic care.

Green Paper on Improving the mental health of the population: Towards a strategy on mental health for the European Union[41]***.*** The document was released in November 2005 by the European Commission as a first response to the WHO mental health declaration for Europe. It recognises the importance of promoting the social inclusion of persons with disabilities and mental health conditions and protecting their fundamental rights and dignity.

[39] Available at http://ec.europa.eu/social/BlobServlet?docId=14813&langId=en
[40] http://www.chrodis.eu/
[41] Available at http://ec.europa.eu/health/archive/ph_determinants/life_style/mental/green_paper/mental_gp_en.pdf

European Pact for Mental Health and Well-being[42]. The pact was launched following the 2008 EU high-level conference "Together for Mental Health and Well-Being". Among other things, the pact called for measures supporting the recruitment, retention or rehabilitation and return to work of people with mental health problems.

Joint Action Mental health and Well-being[43]. Launched in 2013 and funded by the EU Health Programme, the action aims at building a framework for action in mental health policy at European level and builds on previous work developed under the European Pact for Mental Health and Well-being. One of the objectives of the action is to promote the social inclusion of people with mental health problems in Europe. It also aims at integrating mental health in various policy areas.

European Parliament resolution of 19 February 2009 on Mental Health[44] calls on Member States to give people with mental-health problems full access to employment, and stresses the need for more research.

Declaration of the European Parliament of 13 September 2012 on tackling multiple sclerosis in Europe[45]. This Declaration called on the Commission and the Council to promote, in their Reflection Process on Chronic Disease, equal access to treatment and flexible employment policies for people with chronic neurological disorders such as multiple sclerosis.

CANCON Joint Action 2014-2017[46] is a joint action initiative co-funded by the EU Health Programme. It aims to develop the European Guide on Quality Improvement in Comprehensive Cancer Control ("CanCon"). One of the work packages, Survivorship and Rehabilitation, has a special focus on employment issues and aims to develop tools, a European "distress barometer", and a personalised rehabilitation and survivorship care plan.

---

[42] Available at http://ec.europa.eu/health/mental_health/docs/mhpact_en.pdf
[43] Available at http://www.mentalhealthandwellbeing.eu/the-joint-action#the-joint-action-for-mental-health-and-well-being
[44] Available at http://www.europarl.europa.eu/sides/getDoc.do?pubRef=-//EP//NONSGML+TA+P6-TA-2009-0063+0+DOC+PDF+V0//EN
[45] Available at http://www.europarl.europa.eu/sides/getDoc.do?pubRef=-//EP//NONSGML+TA+P7-TA-2012-0357+0+DOC+PDF+V0//EN
[46] Available at http://www.cancercontrol.eu/

3.3. EU funding programmes

Three EU Health Programmes

The first EU Health Programme, for the period from 2003 to 2008, aimed to ensure high-level health protection in Europe by focusing on health information, capacity to react to health threats, and prevention of diseases and illness.

The second EU Health Programme, for the period from 2008 to 2013, was implemented by the Consumers, Health, Agriculture, and Food Executive Agency (Chafea) and aimed to promote the prevention and early detection of chronic diseases, as well as to address health inequalities. The programme supported projects that helped to build capacity, exchange good practice, create common standards and encourage evidence-based interventions. It was linked to the EU Strategy for 2008-2013.

The third EU Health Programme, for the period from 2014 to 2020, is targeted at economic and demographic challenges faced by Member States' health systems and at enabling citizens to stay healthy for longer. It has an objective of fostering supportive environments for healthy lifestyles taking into account the 'health in all policies' principle.

The 2011 Proposal for a Regulation of the European Parliament and of the Council on establishing a Health for Growth Programme[47] for the period 2014-2020 has highlighted the importance of health policy, especially in light of the challenges related to demographic change that Europe is facing, as well as the need for action to reduce inequalities in health as a condition for inclusive growth.

The EU funding programmes include the ESF, which is designed to support jobs, help people get better jobs and ensure fairer job opportunities for all EU citizens. The programme provides funding to employment-related projects, among which are projects supporting the employment of people with chronic illnesses[48].

Another funding instrument is the Employment and Social Innovation (EaSI) programme, which promotes sustainable employment, combats long-term unemployment and guarantees adequate social protection, social inclusion, and decent working conditions.

---

[47] Available at http://eur-lex.europa.eu/legal-content/EN/TXT/PDF/?uri=CELEX:52011PC0709&from=EN

[48] For example, the Solkraft initiative in Sweden and projects supporting the municipalities of Haarlem and Zandvoort in the Netherlands to help people return to work after a chronic illness.

The Rights, Equality and Citizenship Programme 2014-2020 provides funding for training and awareness raising activities, as well as to European Non-government organisations (NGO) promoting non-discrimination and rights of persons with disabilities and other groups at risk of discrimination.

Commission investments in Health:

- Horizon 2020: health research – EUR 6 billion
- Europeans Structural and Investment Funds for Health - EUR 5.3 billion
- EU Health Programme – EUR 449 million.

3.4. Policy tendencies in Europe

As mentioned earlier, in general, two main types of policy approaches can be distinguished, based on the orientations of such policies: the first policy approach is rather passive and is stronger in compensation measures or benefit transfer programmes, while the second one is more active and scores highly on employment integration policies (See more on the typology of disability policies in OECD (2003) report "Transforming Disability into Ability").

Difficult economic situation in Europe has led to the reduction in social protection expenditure and restricted the access to sickness and disability benefits in 2011 in most EU Member States (OECD, 2010). A study by Saltman and others (2011) found that financial pressure and slower economic growth in Europe have led to decreased funding for healthcare and necessitated reforms to improve the sustainability of public funding of healthcare. Financial pressure has led to reforms in pension schemes that aim to extend working life. Such reforms have made the withdrawal of older workers from the labour market in case of unemployment less likely than before (European Commission, 2015).

The growing financial pressure, coupled with the willingness to increase the labour market participation and the inclusion of persons with disabilities, has caused disability policies in many European countries to shift from a passive compensation approach towards a more employment-orientated integration approach (OECD, 2010). Social assistance schemes for unemployed are increasingly associated with activation schemes, such as job-search support, access to training or individualised support to encourage and assist in a return to employment (European Commission, 2015). However, despite the positive tendency of moving towards activation policies, the shift in

most European countries has been insignificant, which means that employment and rehabilitation supports had a minimal effect on the employment rate among persons with ill-health (OECD, 2010: 11). Most countries still heavily rely on passive benefits (over 95% of total spending), with the exception of Germany, Norway, the Netherlands and Denmark, which allocate over 10% on active labour market programmes (OECD, 2010: 12). Growing inequalities and a high number of people at risk of poverty and social exclusion: over 24% in the EU in 2013 (Social Protection Committee, 2015: 5) call for adequate income supports. As a result, there is a need to find the right balance between adequate compensation and employment activation measures. This tendency can be observed across the EU. According to the findings of the report of the Social Protection Committee (2015), the main feature of policy reforms carried out in EU countries in 2014-2015, was the attempt to link benefits with activation measures.

Most of international and European policy initiatives and documents listed in Part III of the report echo this need to develop employment integration policies to combine them with income support. The review of these documents allows identifying the following common policy approaches for all frameworks:

- **Anti-discrimination and inclusion approach**: persons with disabilities and health conditions should be able to participate in the open labour market on an equal basis as others.
- **Empowerment approach**: persons with disabilities or health conditions should be empowered to reach their full potential through accessing education, training, vocational rehabilitation services, etc.; the focus should be made on their work capabilities and not on disabilities.
- **Integrated services approach:** coordination is required among different sectors such as health, employment, education, businesses, etc.; employment services should be mainstreamed and health policies should be mainstreamed in various areas including labour, employment and industry.
- **Person-centred approach:** focusing on individual needs and preferences of persons with disabilities and health conditions.
- **Evidence-based approach:** Strengthening research, knowledge base and data collection.

These policy approaches are set forward at international and European levels, but they are certainly reflected at national-level polices, too. Part IV of this report explores in more detail various national level policies as well as systems and services available in ten European countries.

IV. National strategies

There is a shared understanding in Europe about the importance of greater labour market participation and employment inclusion for economic and social development. A shift can be observed in most European countries towards activation policies (OECD, 2010). As mentioned earlier, budgetary constraints and the impacts of the economic crisis has led to the contracting of the passive compensation-oriented policy and the expansion of the integration-oriented policy in European countries, although at different scale in different states. Despite having an overall tendency that is headed in the same direction – the direction of activation - the pathway of each country towards promoting employment integration is unique. Comparisons are difficult to make due to differences among countries in cultural, historical and economic backgrounds, in institutional and social settings, in approaches to chronic diseases and disabilities, etc.

This Part of the report presents the pathways of 10 countries, representing different European models, in (re)integrating persons with NCDs into the labour market. Each country report touches upon the following levels and aspects of strategies.

POLICIES	
• **Main legislative frameworks on chronic diseases, mental health and employment**	Are chronic diseases and mental health issues referred to in national laws on support in employment participation?
• **Main legislative frameworks on disability and employment**	In many cases persons with NCDs fall under the definition of persons with disabilities. In such cases, policies on supports in employment participation of persons with disabilities are relevant for the purposes of this study
• **Main policy provisions on mainstream and specialist employment programmes**	The degree of integration between mainstream and specialised employment services varies across countries and plays an important role in the accessibility and flexibility of services for persons with different needs.
• **Main policy provisions on access to employment**	Depending on national laws, specific policies on employment integration can be made available to restricted groups of people (e.g. persons at risk of discrimination, young people, people with officially recognised disabilities, etc.). The fact whether persons with NCDs can fall under these categories determines their eligibility to specific provisions under these policies.

• **Main policy provisions on stakeholder cooperation and integration of services**	Persons with NCDs may face different types of barriers of different nature (e.g. health, psychological, work-related etc.) and therefore require services that would tackle simultaneously different aspects of their integration. Thus, cooperation between healthcare professionals, employment services, social services, employers and users themselves may allow tackling individual needs in a comprehensive way.
• **Main policy provisions on persons-centred approach and individualised service provision**	People's employment needs vary depending on the level of their work experience and education, the state of their health and symptoms of their conditions, specificities of their work, etc. Thus, the provision of individualised support gives the possibility to target interventions at the specific needs of the individual.
• **Main policy provisions on localised and customised employment service provision**	The availability of decentralised services can impact the efficiency of services and the diversity in service provision by local actors.
**SYSTEMS**	
• **Employment support:**	Depending on national policies, persons with NCDs may be entitled to support in different types of employment.
- ***open labour market***	Persons with NCDs can participate in the open labour market on equal footing with others but health conditions may limit their work-related activities. In such cases, support measures are required to integrating or keeping workers in the open labour market. An example of such measures is *supported employment*, a scheme that supports people with disabilities or other groups with support needs in obtaining and maintaining paid employment in the open labour market.
- ***social enterprises or social cooperatives***	Social economy entities, such as social enterprises and cooperatives, play an increasingly important role in providing opportunities for employment integration to persons with limited work capacity (European Commission, 2013). The main characteristic of such entities is the dual focus on (viable) commercial activity and social objectives.
- ***sheltered work***	Sheltered work has faced criticism as a form of employment that is out-dated for a potentially segregating nature that some sheltered workshops may have. However, sheltered workshops still widely operated and provide jobs to people who were not able to enter the open labour market.

• **Incentives:**	One of the ways to encourage employment activation is to provide incentives.
- ***for persons with NCDs to participate in activation programmes***	Incentives may be created for persons with NCDs looking for a job or trying to stay in one. Participation in employment and employment activation schemes can be stimulated by benefits or simply by the possibility to combine work-related activities with the maintenance of disability benefits.
- ***Financial incentives for employers to recruit/retain persons with NCDs***	Financial incentives can be given to employers to encourage them to hire or keep persons with illnesses or disabilities. This can be done through wage subsidies, tax incentives, preferential treatment in awarding public contracts, etc.
- ***Non-financial incentives for employers to recruit/retain persons with NCDs***	Apart from financial incentives, employers can have other incentives to hire persons with disabilities (e.g. diverse workforce, corporate social responsibility).
• **Obligations:**	Apart from incentives, there can be also a system of obligation to require undertaking measures encouraging labour market participation by persons with disabilities or health conditions.
- ***Duties of persons with NCDs***	Persons with reduced work capacity can be required to actively participate in employment rehabilitation programmes before or while receiving disability benefits.
- ***Duties of employers***	The most common way to oblige employers to hire or retain persons with disabilities or health conditions is the system of quotas.
**SERVICES**	
• **Examples of general and specialised employment services for persons with NCDs**	Common characteristics of specific health conditions and methods of their treatment may require different combinations of employment rehabilitation services. The availability of services customised to chronic diseases (and not to disabilities) or to specific categories of chronic diseases can have an impact on effectiveness of integration.

The information concerning strategies available in each country, which is presented in the following sections, has been collected by means of questionnaires and interviews conducted in each country (See Annex 1).

National strategies for the (re-)integration of persons with NCDs largely overlap with strategies on the (re-)integration of persons with disabilities. Therefore, most of strategies available for NCDs are the same as for persons with disabilities.

4.1. Scandinavian model

4.1.1. Norway

Main legislative frameworks on chronic diseases, mental health and employment

The main policy framework that is applicable for the employment of persons with chronic diseases in Norway is the tripartite Inclusive Work Environment Agreement (IA Agreement), which was first signed in 2011 between social partners (represented by employer and employee organisations) and the Norwegian Labour and Welfare Service (NAV) (represented by NAV Inclusive Workplace Support Centre). The agreement aims to improve the working environment, enhance presence at work, reduce sick leaves and prevent exclusion and withdrawal from working life. The important feature of this policy document is that it assigns more active roles to employers and employees in preventing early retirement, in recruiting and retaining workers with reduced work capacity, and in reducing sick leaves (the effectiveness of the IA is evaluated in the OECD report on Mental Health and Work: Norway[49]).

In terms of policies specific to one of the categories of NCDs considered in the study, there is a National Strategic Plan for Work and Mental Health 2007-2012[50]. The strategy concentrates on five main areas: 1) collaboration and cooperation of work-related help, 2) user involvement and self-help, 3) availability of measures and services, 4) competence, networks, information and attitudes, and 5) knowledge, research and development.

Main legislative frameworks on disability and employment

In 2011, the government of Norway launched the Job Strategy for People with Disabilities[51]. The strategy aims to include more people in employment and reduce the number of benefit recipients. It aims to reinforce one of the main goals of the IA Agreement - to increase the employability of people with disabilities. The main target group of the strategy is people with disabilities under the age of 30. The strategy identifies four barriers for labour market participation (discrimination barrier,

---

49 https://www.regjeringen.no/globalassets/upload/AD/publikasjoner/rapporter/2013/Mental_Health_and_Work_Norway_2013.pdf
50 https://www.regjeringen.no/globalassets/upload/HOD/Vedlegg/Planer/I-1127EWEB.pdf
51 https://www.regjeringen.no/en/dokumenter/jobs-strategy-for-people-with--disabilit/id733535/

cost barrier, productivity barrier and information and attitudinal barrier), and proposes concrete initiatives to tackle these barriers. For example, the strategy proposes having working-life coaches at Inclusive Workplace Support Centres in each county to assist employers.

- The Work Environment Act (Arbeidsmiljøloven): § 4-6 on Accommodation for employees with reduced working capacity.
- Discrimination and Accessibility Act.
- Labour Market Act (Arbeidsmarkedsloven)
- The Social Insurance Act (Folketrygdloven), Section 11 on rehabilitation benefit and Section 12 on disability benefits.
- UNCRPD was ratified in 2013.

Policy provisions on mainstream and specialist employment programmes

NAV, through its local branches, is responsible for mainstream employment services. It has a general national database of job vacancies through which job matching can be done. A similar service is also provided by private commercial entities. NAV provides a variety of services, including Job Club (Jobbklubb), a short-term course for those who are registered as job seeker with NAV. There are no formal entry requirements. In the Job Club, job seekers receive guidance on how to market their skills to employers, how to design CVs and applications to specific jobs based on job analyses, how to behave in interviews and use network to obtain the job.

Apart from general employment services, NAV has special obligations towards people with “reduced ability to work.” The authority can facilitate employment in the open labour market and also in sheltered workplaces. NAV addresses the needs of persons with reduced work capacity, but it has a more targeted approach towards young persons with disadvantages in employment (under 30 years). For instance, secondary schools are expected to provide personnel who can advise students, including those with disabilities, about future employment.

Policy provisions on access to employment support

“Reduced ability to work” can give persons in Norway access to additional support in employment. Work capacity is defined as the person's ability to achieve and retain paid work. A supervisor/advisor in the NAV assesses individual cases based on information from the person as well as from

documentation from the general practitioner, therapist, education system, social workers, etc. The information is balanced against the opportunities to achieve or retain paid work in the labour market. NAV identifies any gap between individual capabilities and the demands and requirements in working life. Work capacity is not measured by medical expertise alone, but diseases, illnesses or impairments are often the main reason for reduced working capacity. In cases where an employment relation exists already, the person will be reviewed in light of her/his working capacity in that specific position. In such cases the employer and employee review the options together. NAV assesses the case on the basis of information provided by employer, employee, vocational education or training, doctor, etc. This includes assessment of education, work experience and other competences. The assessment is intended to be holistic and based on professional discretion in each individual case.

Policy provisions promoting stakeholder cooperation and integration of services

Norwegian policies promote collaboration between NAV and local authorities and health services to ensure that users receive coordinated assistance. NAV has been also developing collaboration with education authorities to prevent school drop-out, service providers and local employers. NAV itself is a result of a merger of a public employment service (PES), a national insurance office and local social services. IA Agreement is example of an attempt to involve employers and social partners in the process.

Policy provisions promoting persons-centred approach and individualised service provision

NAV provided individualised assessment (Avklaring) services to employed or unemployed persons to identify or test their ability to work. These services are important for identifying and assessing individuals' expertise, possibilities and needs in employment.

A system of individual activity plans is in place at NAV for users with emphasis on work and work-related issues. Each individual registered with NAV with an aim to enter or stay in the labour market gets an individual assessment based on their needs, preferences and competences and participates in creating an individual activity plan. These activity plans are tailored to each individual and may contain different employment schemes and supports.

Policy provisions on localised and accessible employment service provision

In Norway, local authorities and central government cooperate through 456 NAV offices in municipalities and city boroughs. Each local authority and NAV agree on what local authority services their office should provide. The services provided by a NAV office thus vary across local authorities. Despite working at local levels through its branches, the decentralisation of NAV's employment activation activities can be questionable. Services delivered by service providers are highly dependent of NAV requirements, thus they are relatively inflexible in adapting to local needs.

NAV also regulates a national database of job vacancies and employers are obliged to post job announcements on the NAV website. However, there are still commercial entities providing services related to vacancy databases.

Activation services for persons with reduced work capacity, including persons with NCDs, are mainly the responsibility of NAV. The impact of NAV is considerable on the sector through its definition and funding of employment schemes. NAV provides supports to persons with reduced work capacity and may require beneficiaries of its support to undergo job search/retaining activities specified in individual activity plans. Such activities can be provided by a wide range of entities that offer vocational rehabilitation services. Many of such service providers are united under the umbrella of the Association of Vocational Rehabilitation Enterprises (AVRE). Vocational rehabilitation services provide various services related to job placement in the open labour market or sheltered work. The services are based on measures and specifications issued by NAV.

Employment support in the open labour market

The main supported employment scheme is called a follow-up (oppfølging) programme, which is intended for persons who require support to be able to find or keep a job in the open labour market. The programme is tailored to individual needs based on opportunities in the labour market and includes guidance, advice and assistance in developing work-related and social skills, job search or workplace adaptation. The programme is overseen by NAV, which has agreements with a number of coaches who provide support in a transitional period. The follow-up measure may last for up to six months and extended by another six months (for special needs it can last up to three years).

A Follow-up programme intended to provide long-term support for workers with mental health issues is based on the Individual Placement and Support (IPS) approach that involves continuous individual follow-up in rehabilitation process as well as parallelism and integration between treatment and employment services. In addition, follow-up measures for persons with mental health issues are specifically aimed at preventing drop-outs from rehabilitation programmes as it seems to be often a case within this group of people.

In addition to follow-up measures, NAV can provide funding for a mentor, a fellow colleague who provides extra assistance to master a job or training.

In order to support employment in the open labour market, NAV also provides funding and guidance in workplace adaption, including assessment of needed adaptations and contribution to cover the costs of special equipment and assistive technologies at work. Inclusion grants (Inkluderingstilskudd) are available for employers to compensate for costs of workplace adaptation and/or costs associated with the recruitment and work-oriented monitoring of persons with reduced work capacity (up to NOK 115,200 over a period of 12 months from the date the first expenditure).

The support by NAV not only covers the needs of adapting existing circumstances to facilitate the integration of persons with reduced working capacity, but also to prevent the deterioration of health conditions and sick leaves. For example, NAV also oversees the programme on health and rehabilitation services for people on sick leave (Raskere tilbake). This programme includes follow-up measures by employees and employers aimed at a quick return to work.

Sick-leave compensation can be granted if an employee has a prolonged or chronic disease that leads to a serious illness or a frequent absence for a limited time. A request to cover the sick leave can be filed with NAV by employer or employee.

Employment support through Social enterprises or social cooperatives

No information available.

Employment support through sheltered work

Traineeship in a sheltered business (arbeidspraksis i skjermet virksomhet) is an employment scheme for those who need to improve their opportunities for entering the work force or starting an education. It allows testing the ability to work in a sheltered environment. A traineeship in a sheltered business is only available for persons with an impaired work capability who have particularly uncertain professional capabilities and who require close and broad supervision and assistance. Sheltered businesses produce goods and/or services and provide assessment, job training or qualification services for people with reduced working capacity. The aim of the initiative is to strengthen the ability to find work. Greater collaboration between sheltered and open labour market work is promoted in order to ensure smoother and flexible transition between the two types of work. This is needed to fulfil the need of some persons with mental health issues in stability and security.

Permanent adapted work (varig tilrettelagt arbeid) is a work scheme for persons who receive a disability pension from NAV and need to work in a sheltered environment to participate in working life. In this type of work tasks are permanently adapted to individual capabilities. Permanent adapted work can also be offered in the form of individual placement at an ordinary business or organisation. The aim of the scheme is to develop skills needed to qualify for an ordinary job. Participants of this programme can have a temporary leave from the sheltered business to try themselves in the open labour market (so-called hospitering). The goal of this measure is to assist participants during the transition from a sheltered business to an ordinary job.

Incentives for persons with NCDs to participate in activation programmes

Work assessment allowance (Arbeidsavklaringspenger, AAP) is an income support paid by NAV in periods when persons are ill or injured and need assistance to return to work. The allowance (66% of income in previous job) paid when work capability is impaired by at least 50% and when there is a possibility that the work capacity can be improved through treatment, an employment scheme or follow-up by NAV. A condition for receiving the AAP is to contribute actively in the process of returning to work (duty to participate in creating an activity plan, attend meetings, participate and complete the activities in the activity plan)

Qualification benefit (Kvalifiseringsstønad), worth the double of the National Insurance basic amount (the National Insurance basic amount equals to NOK 90,068 as of 1 May 2015), can be paid to persons participating in the Qualification Program (described in later sections).

Support when participating in measures (Tiltakspenger) – financial support covering day to day expenses while participating in the employment measure (low rate – NOK 257 per day; high rate – NOK 355 per day). This can be paid, for example, when a person participates in the Follow-up programme and does not receive sickness benefits, unemployment benefits or AAP.

A person may receive partial disability benefit if her/his work capacity is reduced by a minimum of 50% due to sickness and/or impairment. If the person receives a rehabilitation benefit (a temporary and time-limited benefit), a 40% reduction in work capacity is sufficient. If the reduced working capacity is due to a work related sickness or impairment, it is sufficient that the ability to earn a living is reduced by 30%. The benefits are paid to insured persons of working age. Relevant treatment and vocational training must be completed before disability benefit is granted. Disability benefits and rehabilitation benefit can be combined with the receipt of salary.

Financial incentives for employers to recruit/retain persons with NCDs

Wage subsidies, provided by NAV, cover part of the employee's salary if she/he is in danger of losing her/his job for health reasons (including high sickness absence related to chronic disease or pregnancy). There are temporary and permanent wage subsidies, but they are not a major part of the Norwegian labour market inclusion policy. The temporary wage subsidy is paid by NAV to the employer (60-70% of the salary) for a limited period of time to compensate for lower productivity. The measure is applicable for persons having a difficulty entering the labour market on ordinary wage and working conditions. If the employee has a reduced work capacity, the subsidy can be provided for a maximum of 3 years. Permanent wage subsidy targets persons who have a permanent and significant reduction in work capacity. Employers may have 75% of their costs covered the first year and 67% in the following years. Since 2007, Norway has had a pilot program with permanent wage subsidies, limited to 2,500 cases. In 2014, the program became permanent and without a formal ceiling on the number of beneficiaries. The program is intended to prevent permanent receipt of disability benefit.

Non-financial incentives for employers to recruit/retain persons with NCDs

The Ripples in the Water programme is based on a methodology for the cooperation between businesses and vocational rehabilitation service providers. The programme emphasizes socioeconomic benefits of integrating employees with reduced work capacity. The idea is that companies can fulfil their labour needs with a successful fulfilment of their corporate social responsibility strategies. Service providers can, on their part, provide follow-up and coaching services for the employees with reduced work capacity. The programme is collaboration between the Confederation of Norwegian Enterprise (NHO) and AVRE.

Duties of persons with NCDs

Persons with reduced work capacity are required to go through relevant treatment and vocational training before a benefit is granted.

Duties of employers

In Norway, there is no quota system for employers. In fact, Norwegian employers are not subjected to any strict obligations and receive considerable support from government authorities in terms of integration measures for persons with reduced work capacity (informational measures and services in recruiting and retaining workers).

Public employers also do not have to fulfil quotas, but there is a "The State Shows the Way" approach, in which the public sector is supposed to set an example in employing persons with reduced work capacities.

General and specialised employment services for persons with NCDs

NAV provides funding for different types of education and training aimed at equipping persons with reduced work capacity with necessary skills and qualification:

For persons with impaired work capabilities, NAV may provide financial support for ordinary education or vocational training.

Qualification Programme (Kvalifiseringsprogrammet, KVP) is available for persons of working age with reduced work capacity who have undergone work assessment and receive no or limited benefits from NAV. The programme is based on the individual needs and circumstances and includes work-oriented activities, training activities, and close individual supervision and guidance. The programme is planned by a NAV supervisor and the user. It lasts up to one year and includes 37.5 hours per week (NAV can extend the program for another year if it finds it appropriate and necessary).

IA-space (IA-plass) is a special arrangement among companies that have signed the agreement on an inclusive workplace (IA) to provide internships for persons with reduced work capacity. IA-space allows participants to undergo internship in the open labour market to obtain necessary work experience.

Courses, training and internships aimed at preparing persons with impaired work capacity can be provided by vocational rehabilitation entities. Their services may include various forms of "Work Preparatory Training," assistance in obtaining ordinary education, traineeship in sheltered work, job coaching, supported employment, etc.

In addition, service providers can develop their services that fulfil NAV requirements in terms of facilitating the activation process. Jobpics, for example, is a tool developed by the AVRE to work with vocational interests of disadvantaged persons in a systematic way to avoid wrong career choices and to prepare for optimal career choices in the rehabilitation process. The main target group of Jobpics is persons in vocational rehabilitation aimed at joining the labour market.

Some services specifically aimed at the employment of persons with mental health issues include:

- "Personnel with User Experience" training for obtaining a qualification to work in businesses providing services to persons with mental problems.
- Courses on mental health at the workplace to develop the right competences for employer organisations.
- Awareness raising about the normality of having mental health issues and about measures and services available for persons with mental health issues in employment.

4.2. Anglo-Saxon model

4.2.1. United Kingdom

Main legislative frameworks on chronic diseases, mental health and employment

The legislation on employment activation and support cover persons with (chronic) illnesses and disabilities if these conditions affect the ability to work. Different rules apply in the Northern Ireland.

Main legislative frameworks on disability and employment

- Equality Act 2010
- UNCRPD ratified on 8 June 2009

Policy provisions on mainstream and specialist employment programmes

Jobcentre Plus is a PES run by the Department for Work and Pensions (DWP) that provides mainstream employment support to jobseekers. DWP has the Universal Jobmatch portal for vacancies www.jobsearch.direct.gov.uk and Jobcentre job coaches can provide information about available services and integration programmes. There is also a possibility to join Work Clubs (run by local organisations like employers and community groups), which allow unemployed persons to share knowledge, experience in job search etc.

For jobseekers with limited work capacity, assistance can be given by Disability Employment Advisers who can refer them to different specialised employment schemes and services run by private firms, social enterprises or NGOs. Such services include assistance in job search, career planning, vocational guidance, assessment of qualifications,

In order to facilitate the job search process for persons with impaired work capacities, a system has been put in place to label disability-friendly employers. Job announcements posted by companies who are committed to employing persons with disabilities are marked with a "positive about disabled people" symbol (with 2 ticks).

Policy provisions on access to employment support

Under the Equality Act 2010, a person has a disability if she/he has a physical impairment or a mental health problem, and if the condition has a substantial and long-term (12 months and more) adverse impact on her/his ability to carry out normal day-to-day activities. Persons diagnosed with cancer or multiple sclerosis automatically meet the disability definition. Special rules also apply for impairments with recurring and fluctuating effects (e.g. rheumatoid arthritis, epilepsy, mental health conditions such as schizophrenia, bipolar affective disorder, and certain types of depression).

The focus of the assessment is not disability but the work capability. Work capability assessment is needed for determining eligibility for employment and support allowance (ESA). The assessment procedure is initiated by claimants following a self-declaring of a limitation in the capability to work. The DWP officers look at the information provided by the claimant and if it finds evidence of limitations in work capability, they send a questionnaire to the claimant to fill in. The questionnaire includes questions about the ability to complete different tasks. Following this, a face-to-face assessment can be required by a healthcare professional working for the Health Assessment Advisory Service, which is operated by an organisation delivering the assessment on behalf of the DWP. The assessment consists of two parts:

1) Limited capability for work assessment – evaluates the capability to carry out a range of activities (physical, mental, intellectual activities). A confirmed limited capability means that a person can receive the ESA.
2) Limited capability for work-related activity assessment – evaluates whether a claimant should undertake work-related activities to continue receiving the ESA. Based on this assessment, you can be placed in one of 2 groups:
    - Work-related activity group, where persons have regular work-focused interviews with an adviser and are required to comply with strict work-related conditions in order to continue receiving the benefit in full.
    - Support group, where there are no interviews with an adviser and no requirements to undertake work-related activities; the rate of ESA is higher in this case.

Policy provisions promoting stakeholder cooperation and integration of services

In the UK, Jobcentre Plus combines the tasks concerning employment services and benefits payments, thus reinforcing the coordination between benefits and work. In addition, the assessment

of the working co-capacity involves different stakeholders: users (through self-declaration of limitations in work), healthcare professionals, and the Department of Work and Pensions. Services to employers are seen as important, but provisions on services to employers are less consistent and strong at national level. Service providers do work businesses and try to have a certain number of key accounts with whom they can work on strategic basis and on special programmes.

Policy provisions promoting persons-centred approach and individualised service provision

In the UK, individual responsibility is emphasized with regard to unemployment. Thus, the focus is made on the making effort towards an active participation in employment programmes and a quick return to work. Employment services are also individually tailored to the needs of (potential) employees and employers. Support is personalised at targeted at various barrier individuals face (barriers related to health conditions, perceptions of users and employers, secondary mental conditions, environmental barriers.)

Policy provisions on localised and accessible employment service provision

Jobcentre Plus, which is overseen by the DWP, delivers the functions of the PES in the UK and manages a network of local Job Centres that help unemployed people, including disabled people, to find jobs. Jobcentre Plus represents a centralised system of employment activation, but there has been a shift towards greater decentralisation though the granting of more freedom to local offices. Through local branches, Jobcentre Plus can provide services that are more suitable for local needs.

Example: Residential training for unemployed adults with disabilities can be delivered who do not have locally available training courses that suit their needs and can help them get a job, gain experience or become self-employed. There are 9 providers of residential training in England.

The UK has been actively engaged in the marketization of employment activation services. As mentioned earlier, high priority has given to the development of social enterprises that operated on competitive and financially sustainable basis. In addition, in 2015, a large employment service, Remploy, exited from government ownership and entered the private sector. As a private entity, Remploy provides services to state employment service Jobcentre Plus or to local authorities. This

has lessened the control of the government over employment services to persons with support needs and gave a competitive feature to the sector.

Employment support in the open labour market

Different programmes are available for supporting employment in the open labour market:

- **Work Programme** provides mainstream employment scheme that provides support, work experience and training for up to 2 years to help people find and stay in work.
- **Work Choice Programme** is a specialised employment support aimed at helping:
  - Persons with a recognised disability under the Equality Act 2010 to get or keep a job though individually tailored supports that can include training and skills development, interview coaching, confidence building.
  - Employers to hire or keep a disabled person who requires special support.

  Work Choice is delivered by various organisations from the public, private and non-profit sectors. These providers are tasked to develop a package of support specially tailored for businesses and the individuals with the aim of developing their skills (e.g. Remploy, which delivers Work Choice nationally, and national charity Shaw Trust).

- **Access to Work grant** - can be used for paying for practical support for persons with a disability, health or mental health condition in starting to work, staying in work or moving into self-employment or entrepreneurship. It can be used for adaptations to equipment, special equipment, travel arrangement, a support worker or job coach, a support service per persons with a mental health issues, disability awareness training for staff, a communicator at a job interview, and the cost of moving equipment. As of October 2015, the Access to Work grants are capped at GBP 40,800 per year.
- **Sector-based work academies** provide training and internships for up to 6 weeks in a particular industry or area of work. Such academies may offer a guaranteed interview for a job or an apprenticeship.

Protection and privileges:

The dismissal is considered fair if the employee cannot do their job due to a long-term illness because there are no reasonable adjustments that can be made, even if the employee is disabled.

Flexible labour marker in recruitment and dismissal is a characteristic of the UK system.

Employment support through Social enterprises or social cooperatives

The social enterprise sector is very diverse in the UK and its development has been high on the national agenda. Social enterprises take various forms but the main focus of such businesses should be put on social purposes and a reinvestment of profits into the social objective of the business and the benefit of the community.

UK social enterprises focus on different sectors and social purposes, including the purpose of integrating vulnerable groups and persons with disabilities into the labour market. One example is social firms. These are businesses that provide jobs or training opportunities for people at risk of labour market discrimination. Social firms fan be focused on employment (creating real jobs), on employability (providing work experience, training, or coaching) or both. For example, PLUSS (www.pluss.org.uk), one of the largest social firms in the UK, directly employs persons with disabilities and long-term health conditions, and runs employment operations that provide in job seeking to persons with special needs.

The UK Government has introduced the Social investment tax relief to encourage financing and support to social enterprises. Individuals who make eligible investments in social enterprises can have 30% of the cost of the investment deducted from the income tax liability (the investment must be held for a minimum period of 3 years for the relief to be retained).

In addition, an independent financial institution, Big Society Capital, has been created to make social investment available for social enterprises.

Employment support through sheltered work

In 2012, the UK Government withdrew funding from segregated sheltered workshops in favour of developing social enterprises instead. Many sheltered workshops that were run by then-government

agency Remploy were closed down or taken over by other entities focused on social entrepreneurship.

Incentives for persons with NCDs to participate in activation programmes

Jobseeker's Allowance can be paid to persons to help them when they look for a job. In order to receive the benefit, a person has to go to an interview at a local Jobcentre Plus office and fulfil the agreement to look for a job.

Persons (employed, self-employed, unemployed or student on Disability Living Allowance) with an illness or disability that affects their ability to work can apply for the ESA. The allowance is not paid if a Work Capability Assessment finds that a person is capable of doing some work.

The ESA can be compatible with so-called "permitted work," i.e. a work that generates income not exceeding GBP 20 a week or a work for less than 16 hours a week and income of up to GBP 115.50 a week, for 52 weeks or less (or for any length of time if a person is in the support group). In addition the ESA can be compatible with "supported permitted work," which is a work, with an income of up to GBP 115.50 a week, that is part of a treatment programme, or supervised by someone from a local council or voluntary organisation whose job it is to arrange work for disabled people.

Financial incentives for employers to recruit/retain persons with NCDs

UK employers do not receive direct financial incentives in a form of wage subsidies or tax reductions for hiring persons with disabilities.

According an official of employment service provider Remploy, wage subsidies can be harmful as they send a wider message that persons with disabilities are not capable of working and therefore an additional compensation needs to be paid to incentivise employers. For this reason, money would be better spent on supports for employment.

Non-financial incentives for employers to recruit/retain persons with NCDs

Disability Confident Campaign is a government-backed initiative, through which employers can commit to become Disability Confident. Becoming Disability Confident entails hiring and retaining people with disabilities or with health conditions for their skills and talent. The aim of the campaign is to challenge stigma against disability in employment, raise awareness and remove barriers in employment.

With the absence of financial incentives for employers to hire persons with impaired working capacity, agencies handling the work placement of these groups rather rely on making a "business case" when dealing with employers, in other words, they provide business arguments in order to encourage employers to recruitment their clients. For example, some of arguments used by Remploy are:

- Reduced staff turnover as persons with impaired work capacity tend to stay longer and prefer greater stability;
- Access to a big pool of disability talent;
- Having a diverse workforce that reflects the community they serve (persons with user experience can help businesses better meet disabled customer's needs and thus expand the customer base).

Duties of persons with NCDs

- Jobseeker's Allowance is conditional on participation in job activation measures.
- ESA is conditional on participation in job activation measures when persons are required to participate in work-related activities following a Work capability assessment.

Duties of employers

There is no quota system.

General and specialised employment services for persons with NCDs

- Under the Work Choice Programme, persons with long-term health conditions who are looking for a job or are trying to keep one can get support for training and development of skills. The services are contracted from private, public or non-profit sectors.
- Sector-based work academies provide training and internships for up to 6 weeks in a particular industry or area of work. Such academies may offer a guaranteed interview for a job or an apprenticeship.
- Fit for Work provides the services of occupational health professionals to employers in England and Wales who have employees that have been, or are likely to be, off work due to sickness for four weeks or more. Following the referral to Fit for Work by an employer and a consent from an employee, the employee is contacted by an occupational health professional for an in-depth consultation that looks into health-related, work-related and personal issues that might be preventing a return to work. After that, a personalised Return to Work Plan can be developed. This service allows small and medium companies that do not have occupational health professionals to fill in this gap.

Mental health:

- Rethink Mental Illness provides employment and training services to people with severe mental conditions with an aim of accessing/retain jobs, regaining self-confidence and independence. The organisation is part of a partnership implementing IPS services to persons with disabilities.

Cancer:

- Shaw Trust provides work support for persons with cancer through a service model that is based on three levels: 1) providing basic information, 2) assessing the patient and 3) implementing a personalised plan. The service is based on cooperation between stakeholders involved in the care process (health centres, employers, employment services, local authorities and families). Specialised services include advice on careers and dealing with colleagues, specialised guidance to manage a condition, training for employers and families, advice on employment rights and adjustments to work, assistance with return-to-work plans and mediation of disagreement.

4.3. Continental model

4.3.1. Austria

Main legislative frameworks on chronic diseases, mental health and employment

“Chronic diseases” or “Chronically ill” are categories which as such are not part of common terminology in social insurance (including employment insurance) or health regulations.

Disability and unemployability (arbeits- bzw. erwerbs(un)fähig) are the major terms used in the General Social Insurance Act. A (chronic) disease may be at the origin of an officially recognised disability status (“Behindertenpass”) attesting a reduction in work or earning capacity (usually expessed as ‘degree of disability’ in percentages); on the other hand, a person with a chronic disease may not receive the status of an officially recognised person with a disability and may also have full work or earning capacity.

“Reduced working capacity” exists within the health insurance only in terms of a physician telling one that one cannot go to work and must stay at home on sick leave (may last up to 26 weeks, in specific situations up to 52 or even 78 weeks). If this reduced working capacity persists, e.g. in case of a chronic disease, persons with chronic diseases may apply for rehabilitation allowances (instead of sickness-leave allowances) from their medical insurance for a limited time period (up to 2 years)[52]. Within this period, they are coached by rehabilitation advisers / case managers. After the assigned time period, vocational re-assessment is foreseen.

A person with a chronic disease may be considered “unemployable” or “incapacitated for work” (“arbeitsunfähig” as stated in a vocational assessment report or “Berufsdiagnostisches Gutachten”). Then she/he might get a permanent invalidity pension.

For certain services and advantages (e.g. employment within the quota system as part of the Austrian Disability Employment Act), persons with chronic diseases need to get the status of “disabled persons”; for other services (e.g. in the fit2work program as part of the Austrian Work and Health Act), the fact that they are (chronically) ill or have been ill is sufficient.

[52] General Insurance Act has changed - since 2014 access to permanent pension (Invaliditäts- und Berufsunfähigkeitspension) is limited to those born before 1964; all other persons with chronic diseases receive rehabilitation allowances from their medical insurance for a limited time period (up to 2 years) and are coached by rehabilitation counsellors/case managers. After this period, vocational re-assessment is foreseen.

Within the General Social Insurance Act, no "partial disability" status is foreseen. One is either completely fit or completely unfit for work (which is an obstacle for gradual return to work). In the field of unemployment support and in the employment policies a (partially) reduced working capacity may be a reason to receive (re-) training or adaptations or allowances.

Main legislative frameworks on disability and employment

- The General Social Insurance Act (Allgemeinen Sozialversicherungsgesetz, ASVG)
- The Austrian Social Law Amendment Act (Sozialrechtsänderungsgesetz 2012)
- The Austrian Work and Health Act (Arbeit-und-Gesundheit-Gesetz, AGG, 2011)[53]
- The Austrian Disability Employment Act (Behinderten-Einstellungsgesetz, BEinstG 1970)[54]
- The Austrian Law on Continued Payment of Remuneration (Entgeltfortzahlungsgesetz - EFZG)[55]
- Ratified the UNCRPD on 26 September 2008.

Policy provisions on mainstream and specialist employment programmes

Persons with chronic diseases / disabilities can use all mainstream services of the Public Employment Service of Austria (AMS). There are Rehab-Counsellors at the AMS and a variety of services offering counselling for persons with difficulties finding work, targeting younger persons , women, persons over 50 years, and persons with disability. Furthermore, a specific service organisation of the Federal Ministry of Labour, Social Affairs and Consumer Protection, called Sozialministeriumservice (SMS) is the main governing body dealing with employment schemes for people with disabilities.

Policy provisions on access to employment support

In Austria, competence centres of the Austrian Federal Pension Fund (Pensionsversicherungsanstalt, PVA) are primarily responsible for the assessment of reduced work capacity due to chronic disease ("Gesundheitsstraße"). Furthermore, the SMS is responsible for acknowledging an officially

[53] https://www.ris.bka.gv.at/GeltendeFassung.wxe?Abfrage=Bundesnormen&Gesetzesnummer=20007058
[54] https://www.ris.bka.gv.at/GeltendeFassung.wxe?Abfrage=Bundesnormen&Gesetzesnummer=10008253
[55] https://www.ris.bka.gv.at/GeltendeFassung.wxe?Abfrage=Bundesnormen&Gesetzesnummer=10008308

recognised disability status (Behindertenpass) attesting a reduction in work or earning capacity (usually expressed as ‘degree of disability’ (‘Grad der Behinderung’, GdB) in percentages). For some special employment-related interventions, a degree of 50% is required. The disability assessment, based on a specific taxonomy (Einschätzungsverordnung, 2010), covering all organ systems and medical conditions, is primarily medically and disability-focussed. The disability status might not always be permanent; the assessor decides if and when a re-assessment is necessary.

The 50% degree of disability separates the responsibilities between the Austrian State government and the federal provinces. The federal provinces are responsible for interventions for persons with a degree of disability below 50%; these are persons who might face difficulties in acquiring an employment contract with all the social security rights and obligations attached to it. The Austrian State government is responsible for the interventions for all persons with a degree of disability above 50%.

The affected person her- or himself, other authorities such as the AMS, the Austrian Federal Pension Fund (Pensionsversicherungsanstalt) or other rehab institutions can induce such an assessment by SMS. Here a person is found to be disabled or unemployable and therefore might be entitled to benefits of medical or professional rehabilitation.

According to the social insurance laws one is either capable of working or not (no partial disability pensions). This might raise a barrier for reintegration of persons with chronic diseases. However, assessments to find out how to (re-)integrate persons with chronic diseases into the labour market takes an approach focussed on what a person “can do” instead of what the person “cannot do” (as is done by case-managers in the fit2work approach, see next point).

Policy provisions promoting stakeholder cooperation and integration of services

Changes in legislation towards “Step by step integration” have been made in 2011 (The Austrian Work and Health Act). As a result, a “fit2work” programme, an advisory and prevention programme for older employees and persons with chronic diseases / disabilities has been established.

The fit2work activities are coordinated by the SMS. Inter-agency cooperation and integration of services are main features of the fit2work service program, provided by regional implementing partners cooperating with many other partner organisations, such as the Association of Austrian

Vocational Training and Rehabilitation Centres (Berufliches Bildungs- und Rehabilitationszentrum, BBRZ-Gruppe)[56].

First results of monitoring the interventions raised by the Austrian Work and Health Act are expected by the summer of 2016.

Policy provisions promoting persons-centred approach and individualised service provision

Fit2work provides free advisory service if one's job is at risk because of health problems or if a person is facing difficulties in finding work. Furthermore, fit2work case managers provide personal support if needed. At the beginning, a clearing process takes place to capture individual needs. Next, the consultants set up an action plan in collaboration with the individual. After this, fit2work case managers will support and assist the individual in implementing the action plan.

Policy provisions on localised and accessible employment service provision

The PES has regional offices and there is a nationwide network of fit2work centres since 2012.

Employment support in the open labour market

Supported employment is a national mainstream programme in Austria. It began in 1992 with two pilot projects "Arbeitsassistenz", which provided individual support for persons with mental or physical disabilities. In 1994, this kind of service was incorporated in an amendment to the Austrian Disability Employment Act and the service was extended to a broader target group and made available nationwide. In 2011, a total of 45 different NGO service organisations ran 135 projects. Persons with chronic diseases may benefit from these services, although the effects have not been evaluated systematically.

Adaptation of the working time is dependent on agreements between the employer and the person concerned and may also depend on collective agreements within the different industries or

[56] http://www.bbrz-gruppe.at/de/home.html

employment contract or ‘Dienstrecht”. The employer has to be disposed to organize such adjustments but may get advice.

‘Disability Management’ has been developed in some large companies (e.g. Bank Austria). Specific projects are stimulated, such as ‘Cancer&work’ offering counselling for individuals and businesses.

The SMS offers financial support for eligible persons with disability or for persons at risk to get disabled if no support is provided. Such adjustments may fall under the notion of reasonable accommodation and may therefore be imposed after a court case.

Privileges and protections:

- Some persons with chronic diseases and persons with disabilities (with a care allowance of at least rate 5 of 7) may receive personal assistants who operate (also) at the workplace.
- Persons with an officially recognised disability status may have more leave days than non-disabled persons (depending on collective agreements within the different industries or employment contract or ‘Dienstrecht”).
- Employees with an officially recognised disability status above 50% enjoy an enhanced dismissal protection (“Erhöhter Kündigungsschutz”), which results in a more complex dismissal process. This means a person who has been employed for at least 4 years person can only be dismissed after approval by a committee of the SMS. This raises employers prejudice and concerns to hire persons with chronic diseases or disabilities. In practice the dismission is in many cases approved by the SMS. Still the prejudice and concern with “Erhöhter Kündigungsschutz” persist. So far only 25% of employers meet the employment quota. From the employers’ point of view, dismissal protection limits the autonomy of companies. Therefore, employment specialists sometimes advise their clients not to register for disability status. So it has to be considered that there is a ‘dark figure’ of unknown cases of persons with disabilities.
- The wages of employees with an officially recognised disability status as such may not be lowered because of the disability.
- Persons who lost their full-time job due to a disease and would earn less than before in a new job can get compensation payments for one year, leading to a net income above the unemployment benefit. This means that compensation for loss of income is only compensated for a short period of time for the individual with disability. Employers, however, can receive longer financial support (see section on ‘Financial incentives for employers’).

Employment support through Social enterprises or social cooperatives

Different types of social economy organisations exist. They offer mostly temporary training places and counselling to persons with chronic diseases or disabilities in order to prepare them for the open labour market.

Employment support through sheltered work

The term 'Sheltered work' is no longer in place and has been substituted by “Behindertenhilfe”. Sheltered work is restricted to persons with disabilities with a work capacity less than 50%. Participants have no labour contract and receive no wages, only EUR 90 pocket money. Therefore they do not receive the benefits of an employment (social insurance and coverage by the Austrian pension fund). Most of the about 20,000 persons in Austria receiving support from “Behindertenhilfe” are persons with intellectual disabilities. Transfer to the open labour market is very low.

Incentives for persons with NCDs to participate in activation programmes

There has been a move away from temporary invalidity pensions (which could become unlimited) to rehabilitation allowances. In 2012, the Austrian Social Law Amendment Act was released. As a consequence, persons born after January 1, 1964 and who are not permanently unable to earn a living can no longer receive a temporary pension (14 times per year) but get rehabilitation allowance (12 times per year) instead, accompanied by case management offering medical and vocational services to get back to work. In practice however the vocational part of this system is not well developed yet.

Financial incentives for employers to recruit/retain persons with NCDs

Employers recruiting officially recognized persons with chronic diseases or disabilities are entitled to receive integration subsidies (“Eingliederungshilfe”). The first three months, the subsidy covers

100% of the gross wage + 50% of additional expenses (health and pension insurance etc.). The next nine months, subsidies cover 50% of the gross wages and 25% of additional expenses. After this first year, the employer may be entitled to permanent (year to year) financial support (IBH, Integrationsbeihilfe) from the SMS for employees with disabilities. This covers extra expenses for circumstances related to the disability.

Non-financial incentives for employers to recruit/retain persons with NCDs

There are some (voluntary) programmes in (mostly large) companies promoting the recruitment and retention of persons with ill health. These programmes are as well used for employer branding. Some work-related information aimed at reducing prejudice and fears from employers' side is done by 'Anwaltschaft für Menschen mit Behinderung", the advocacy group for persons with disabilities and the SMS (e.g. workshops, informative talks and trainings are offered, other projects focus on training multiplicators also).

Duties of persons with NCDs

Persons with chronic diseases are required to go through relevant medical treatment and rehabilitation. This is monitored by rehabilitation advisers (mostly restricted to reporting of use of medical system and on-going medical changes).

In Austria the basic concept since the nineties is "rehabilitation before pension", but it was never implemented unequivocally. The reform of the invalidity pension system in 2012 was an important step into this direction. Every one under 50 should undergo rehabilitation, (first medical, then vocational rehabilitation), but this has proven to be impossible for many health conditions. In fact, only a few hundreds undergo rehabilitation, whereas thousands receive rehabilitation allowance. Legislative and service changes are planned with a focus on persons benefiting from vocational rehabilitation.

Duties of employers

Every employer with at least 25 employees in Austria is obliged to employ a registered, protected disabled person for every 25 employees (4%). Some persons (i.e. severely disabled persons, disabled persons of a certain age) are counted double for fulfilment. Non-complying enterprises must pay charges which are not very high: a monthly fine of EUR 248 (small companies) up to EUR 370 (large companies). The fact that employers can 'buy out' of the obligation instead of hiring people with disabilities has for result that 75 % of all the companies stay under the quota obligation and opt for paying the fine.

General and specialised employment services for persons with NCDs

- Association of Austrian Vocational Training and Rehabilitation Centres (Berufliches Bildungs- und Rehabilitationszentrum, BBRZ-Gruppe[57]) offer retraining for different jobs in different fields. Access is restricted and there is no absolute entitlement to receive education and (re)training to obtain additional skills and qualifications.
- Retraining of persons who are on sick leave or receive a rehabilitation allowance is impeded by the notion of "employment protection" (Berufsschutz). This means that someone can only be placed in an appropriate job related to the individual's previous job experience or training. Therefore, a person who performed highly-qualified work has to be treated differently from a person who performed low-skilled work. The former could not be referred to a low-skilled job and might therefore get an invalidity pension (if no suitable job is available) whereas the latter would have to take any low-skilled job on offer and might get no pension. For white collar workers, the same reasoning applies but is based on the level of income. The Supreme Court has ruled that a huge loss of income is unreasonable and could therefore be a reason to award a pension instead of obliging a person to take a job which pays much less than what she/he earned before.
- Within the Fit2work programme, an early assessment and registration of the consequences of a disease should take place in order to early detect a risk of developing a chronic disease, which might impact on work capacity. At the moment, resources for this can be enhanced..
- A few companies implement disability management concepts including staff training as part of the fit2work consulting programme and of specific cancer aid projects.

[5757] http://www.bbrz-gruppe.at/de/home.html

- Fit2work offers counselling and advice to keep people with a health condition at work. Financial support for employer is possible but has to be negotiated between the AMS and the employer.

4.3.2. Germany

Main legislative frameworks on chronic diseases, mental health and employment

Chronic diseases may lead to “disability” or “incapacity for work” and if such assessment is made then all the provisions for people with such a status apply. However, there are some policies at the level of the firm (e.g. Betriebliches Eingliederungsystem (BEM), see further) which aim at (chronic) disease as such.

One of the main challenges in establishing policies for the chronically ill as such or in applying policies for the disabled to chronically ill people is that their situation often fluctuates. With some health conditions, people may be 100% productive today but much less or not at all tomorrow. This calls for tailor-made solutions and not for categories or systems where you’re completely in or out. Personal budgets (see below), “case management” and other individualised measures may in some cases be a solution because they allow for a maximum of flexibility.

Main legislative frameworks on disability and employment

- Code of Social Law II (Sozialgesetzbuch II): Basic provisions for job-seekers
- Code of Social Law III (Sozialgesetzbuch III): Employment Promotion
- Code of Social Law IV (Sozialgesetzbuch VI): Statutory Pension Insurance
- Code of Social Law VII (Sozialgesetzbuch VII): Statutory Accident Insurance
- Code of Social Law IX (Sozialgesetzbuch IX): Integration and Rehabilitation of Disabled People
- Regulation of the severely disabled countervailing charge (Schwerbehinderten-Ausgleichsabgabeverordnung, SchwbAV)
- General Equal Treatment Act (Allgemeines Gleichbehandlungsgesetz)
- Equal Opportunities for People with Disabilities Act (Behindertengleichstellungsgesetz)

Policy provisions on mainstream and specialist employment programmes

The Federal Employment Agency runs local Job centres which provide case management for people who are difficult to place (including disabled people and chronically ill).

The Integration Services (Integrationsämter), of which there were 208 in 2011, aim to improve the labour market participation of persons with severe disabilities in Germany. The Integration Services are public authorities ("Behörde"). Their services are funded by the compensation tax (applied when companies do not comply with the mandatory employment quota for people with disabilities). The Services aim to sustain a link between the Federal Employment Agency (which is responsible for the placement of persons into the labour market) and the Integration Offices (amongst other things responsible for supporting severely disabled people to get the employment help they are entitled to) and Rehabilitation service providers (which are responsible for the re-integration of people with disabilities into the open labour market). The main tasks of the Integration Services include:

- support (financial, information, other) to people with severe disabilities and their employers;
- supervision of the application of the special protection against dismissal for people with severe disabilities;
- training/information courses and public relations work;
- collection and assignment of the compensation tax.

A great deal of the information on employment for people with a chronic disease is part of the information aimed at people with disabilities. There are many agencies, social services and also patient organizations providing information.

A Federal Government Commissioner for Matters relating to Disabled Persons is appointed by the Federal Cabinet for one legislative term. Since 1 May 2002 the Act on Equal Opportunities of Disabled People (Behindertengleichstellungsgesetz, BGG) has defined the tasks of the Federal Government Commissioner for Matters relating to Disabled Persons. The Commissioner provides information about the legal situation, initiates law change, offers practical tips and explains how disabled persons can be integrated into society and the world of work. There are also commissioners in each State ("Landesbeauftragten für Menschen mit Behinderungen)

Policy provisions on access to employment support

There are two important concepts which define the target groups of the policies. Both focus on the disability.

- Determination of a disability (Behinderung) affecting participation in one or more areas of life, including work life. Disability is defined as a restriction of the physical, intellectual or mental functions, compared to the normal condition of people of the same age, which probably lasts more than 6 months and hinders (or threatens to hinder) the participation in social life (Code of Social Law IX). Individuals with a disability can obtain a "disability pass" indicating the severity of the restrictions ("Grade of disability"), but they do not have to. This pass is issued by the social security offices. The percentages are calculated according to guidelines and a list of diseases and impairments developed by medical and legal experts. This pass is required for claiming of tax benefits, rights and compensation of disadvantages. The benefits and supports that a person can claim depend on the characteristics and severity of the disability. Persons with a Grade of Disability of 50% or more are categorized as "severely disabled". However, they can have a full capacity for work. A Grade ≥50% is a requisite to get some benefits (e.g. employment protection) Some individuals with a Grade of Disability of less than 50% but at least 30% are considered to be on a par with severely disabled persons ("gleichgestellt") when without the benefits of the severely disabled status they would not be able to get a job or keep their job. They get some of the benefits available for severely disabled persons. They do not get the following: one extra week holidays (but severely disabled do), free public transport, and anticipated pension for severely disabled.
- Incapacity for work (Arbeitsunfähigkeit) - A person is considered to have a full incapacity for work if she/he cannot work at least 3 hours/day. If this person can work between 3 and 6 hours a day, she/he is considered to have a partial incapacity for work. This decision is made by the pension insurance, based on medical documentation submitted and/or their own medical reports (according to the Code of Social Law VI). The decision is binding for the welfare authorities, professional associations and employment agencies (Code of Social Law II).

  It can also be decided, that a person cannot (or cannot yet) work at the general labour market (due to type or severity of the disability). These persons have the right to work at a sheltered workshop. They are considered to have a full incapacity for work and get a full disability pension and social protection.

This does not mean that there are no policies and practices focusing on the abilities of the person. The whole area of (vocational) rehabilitation e.g. assesses what a person can (still) do to build an individualized (re)-integration programme. The focus on individual abilities is also present in several programmes that are carried out across the federal states of Germany. These programmes cover different aspects, including training and guidance for people with disabilities, job placement schemes, awareness raising, aiding the transition of disabled people from sheltered employment to the open labour market. Initiative Inklusion and Job4000 are examples of such programmes. They combine activities such as awareness-raising, guidance, job placement and training in order to encourage the participation of persons with disabilities in the open labour market.

Policy provisions promoting stakeholder cooperation and integration of services

The Integration Offices (Integrationsämter) and Integration Services (Integrationsfachdienste) work together to integrate people with disabilities in the open labour market. They collaborate with rehabilitation agencies, employers' organizations and trade unions to ensure people with disabilities get the opportunities in the open labour market that they deserve.

However, there remains a need for institutional cooperation. There is an overlap of responsibilities and a massive volume of regulations about which pillar of social insurance is to pay for which rehabilitation measures under which conditions.

Policy provisions promoting persons-centred approach and individualised service provision

There is a strong focus on the individual needs of each disabled person. The integration offices and the integration services are required to take into account the individual needs of every disabled person they work with. This commitment is met primarily through the employment of professionals who have experience working with disabled people – the integration services currently employ around 1,400 psychosocially trained professionals at federal level. Furthermore, the legal stipulations regarding supported employment state that the individual needs of the disabled person must be met, and that only professionals with an appropriate qualification can work alongside the disabled people (i.e. psychosocially trained with an additional pedagogical qualification.

Policy provisions on localised and accessible employment service provision

Many services are organized at a local level (employment offices, integration services, etc.)

Employment support in the open labour market

A legal definition of supported employment was incorporated into section 38a of the Code of Social Law IX in 2009. This was due to several successful pilot projects which demonstrated the instrument's efficacy. One of the key principles of supported employment is the 'first place, then qualify' concept. In this regard, supported employment works to place people with disabilities into employment in the open labour market, subject to social insurance contributions. The disabled person is placed and trained in the company and, if necessary, is provided with workplace support after the training. This ongoing support is characterized by intensive job coaching and was received by some 3000 disabled people in 2014. In most cases, supported employment is coordinated by the local integration service.

Self-employment:

- Most of the help for adaptations at the workplace also apply when a disabled person is self-employed.
- There is also financial support (credit or interest rate subsidies, starting money) and coaching for disabled people starting their own business.

Protections and privileges:

- Severely disabled employees (or "gleichgestellte") who have been working for at least 6 months with the employer benefit from some employment protection. Dismissal of such an employee must be approved by the Integration Office ("Integrationsamt") that has to find out if the disability is not at the basis of the intended dismissal and could be prevented by some adaptation measures.
- The status of severely disabled employee comes with 5 additional days of annual leave, arrangements of the working time are a matter of negotiation with the employer (possibly with the help of the disabled staff representative at the workplace)

- Workplace adjustments are very individualized provisions (reimbursement of the costs may be up to 100%, depending on the case). The Integration Services assess which adaptations are best suited.
- A gradual return to work after a (long-term) absence is possible. This period can last from a few weeks to a few months during which the person can work for a few hours and will keep receiving sick pay, but at no cost to the employer.

Employment support through Social enterprises or social cooperatives

There are integration workshops, integration companies and integration departments which employ severely disabled people. They are part of the open labour market and offer work, vocational support, advanced vocational training and ongoing employment support across the open labour market. The numbers of these projects are continuously increasing.

Employment support through sheltered work

There is an extensive network of sheltered workshops (some 300 000 disabled people in nearly 700 workshops in 2013). People who are not (yet) able to work at the general labour market (due to the type or severity of the disability) may get the right to work in a sheltered workshop.

Incentives for persons with NCDs to participate in activation programmes

To receive a disability pension it is necessary to fulfil the requirements of the Code of Social Law VII (mainly having paid the pension contribution for at least 3 years within the last five years, being insured at least the last five years). Otherwise the person gets only the social security provision due to reduced work capacity as it is stipulated in the Code of Social Law XII (social benefit but no pension). If after the assessment it is considered, that a person is able to work less than 3 hours/day (in any kind of job, not only the learned or regular occupation), then she/he becomes a full disability pension. This person can still have a side job (less than 3 hours/day), but if she/he earns more than a fixed amount, the pension is reduced. If the person can work between 3 or more hours/day but less than 6, then she/he becomes a partial pension (and can also have a side job)

A recent initiative is the "Personal budget for work" which allows disabled people to pay for the (employment) services of their choosing. It was first introduced in 2009 and until 2014 some 900 people had been using it.

Financial incentives for employers to recruit/retain persons with NCDs

There are no permanent wage subsidies for the employment of disabled persons, but the Federal Employment Agency and other institutions (health insurance, pension insurance, etc.) provide some temporary measures, such as integration subsidies and financing of a trial employment (3 months), both as compensation for reduced productivity or low performance of severely disabled people. The amount is not standard but is decided after an individualized evaluation.

There is also a subsidy (awarded by various institutions) for in-firm education and training if this is the only possibility and a special subsidy for particularly affected young disabled people. Employers can also receive support when adapting the workplace to the person with disability.

Non-financial incentives for employers to recruit/retain persons with NCDs

There is an annual (federal) award for good practice in Corporate Social Responsibility. In addition, there is a "National Strategy for Corporate Social Responsibility – Action Plan for CSR – of the German Federal Government."[58]

Duties of persons with NCDs

This is a guiding "rehabilitation-before-benefits" principle but is not clear how and to what extent it is implemented.

[58] http://www.bmas.de/EN/Services/Publications/a398-csr-action-plan.html

Duties of employers

There is an obligation for both public and private sector organizations to employ people with disabilities. In companies with more than 20 employees at least 5% must have the status of “severely disabled” (or “gleichgestellt”). Otherwise, the employer has to pay a "countervailing charge". The amount of this charge depends on the total number of employees and the number of employees with a disability that are employed.

General and specialised employment services for persons with NCDs

- There are very many opportunities for training and re-training e.g. in Vocational training centres (Berufsförderungswerke and Berufsbildungswerke), financed by various rehabilitation agencies (Rehabiltationsträger). The Federal employment agency plays a role as well as the Integration Services.
- Re-training for another job of people who are employed but can no longer perform in their current job often is a problem (especially in smaller firms) because re-training may take long (e.g. up to 2 years) and there is no certainty that there will be such a job-opening in the firm at the time the training is finished.
- Peer-to-peer support is offered by organizations of (self-help) and for people with specific (chronic) health conditions.
- The Betriebliches Eingliederungsystem (BEM) is a Corporate Integration Management system which obliges employers to contact employees who were on sick leave for at least 42 days within a year and offer them the possibility to search together how to reduce the amount of sick days. Employees can deny this offer but it may also lead to placement in another job-position within the firm, re-training etc.

4.3.3. Slovenia

Main legislative frameworks on chronic diseases, mental health and employment

People with chronic diseases are not singled out as a specific group, they are covered by the policies and measures aimed at all the people with disabilities.

Main legislative frameworks on disability and employment

- Implementation of the Principle of Equal Treatment Act;
- Equalization of Opportunities for Persons with Disabilities Act;
- Employment Relationship Act;
- Vocational Rehabilitation and Employment of Disabled Persons Act;
- Pension and Disability Insurance Act;

Policy provisions on mainstream and specialist employment programmes

The Employment Service of Slovenia is the main body providing employment services to the general population, including job-seekers with disabilities, who can receive rehabilitation counselling, information, needs assessment and other services. A rehabilitation counsellor helps create a rehabilitation plan.

The main specialist programmes for people with disabilities are:

- Vocational rehabilitation under the Vocational Rehabilitation and Employment of Disabled Persons Act;
- Vocational Rehabilitation under the Pension and Disability Insurance Act.

Policy provisions on access to employment support

Inclusion in vocational rehabilitation is possible:

- under the Vocational Rehabilitation and Employment of Disabled Persons Act: after a person is assessed by a vocational rehabilitation provider, the person can obtain the status of person with disabilities, what is based on decision taken by rehabilitation commission (on the basis of the assessment) or
- under the Pension and Disability Insurance Act after person is assessed by a *disability commission*.

The assessment of rights under the Pension and Disability Insurance Act, performed by a special commission from the Pension and Disability Insurance Institute of Slovenia (Zavod za pokojninsko in invalidsko zavarovanje Slovenije, ZPIZ), is based on a medical model focusing on disability. According to their remaining work capacity insured persons are classified in one of the three disability categories by the Board of Examiners as follows: **Category I**: when an insured person is not able to perform organized gainful activity or when an insured person is not able to carry out their job, and has no remaining work capacity; **Category II**: when an insured person's work capacity to perform their job has been reduced by 50% or more; **Category III**: when insured persons are not able to perform their job on a full-time basis, yet can work part-time for at least four hours a day, or when their capacity to work in their occupation has been reduced by less than 50%, or when they can still work in their occupation full-time yet are not able to perform their current job.

Persons categorized with the following disabilities are entitled to a disability pension:

- category I;
- category II, if without vocational rehabilitation another suitable job with full working hours would not be possible, however, this is not guaranteed where the person is above 55 years of age;
- category III, if without vocational rehabilitation another suitable job with part-time working hours would not be possible (minimum 4 hours a day), however, this is not guaranteed when the person is above 50 years of age;
- category II or category III if suitable work has not been assured when the person has reached 65 years of age.

The Pension and Disability Insurance Act also provides vocational rehabilitation services, such as: integration in the process of formal or non-formal education, on-the-job-training, adaptations to the workplace, the work environment and the equipment.

The assessment of rights under the Vocational Rehabilitation and Employment of Disabled Persons Act is based on the bio-psycho-social model focusing on ability (see : Rules on Criteria and Method for Acquiring the Status of Person with Disability; Right of Employment Rehabilitation, Assessment of Employment Possibilities of Persons with Disabilities and Rehabilitation Committees, 2005). This results in the following classification:

Level 0: No disability (0-4%), nor the right of employment rehabilitation services;
Level 1: Mild disability (5-25%), no right of employment rehabilitation services;

Level 2: Moderate disability (25-49%) → status of person with disability → right to employment rehabilitation services;

Level 3: Severe disability (50-95%) → status of person with disability→ right to employment rehabilitation services;

Level 4: Complete disability (96-100%) → status of person with disability, but no right of employment rehabilitation services.

During the (interdisciplinary) assessment for the employment rehabilitation services the most appropriate services are offered given the disability, the personal characteristics and the environment.

Policy provisions promoting stakeholder cooperation and integration of services

The Action Programme for Persons with Disabilities (2014-2021) states in the introduction that to achieve greater social integration of persons with disabilities and better regulation of the issues relating to disability, closer cooperation between individual ministries, governmental institutions, disabled people's organizations and other organizations addressing issues relating to disability must be enhanced.

Under point 3 Action Programme Objectives / Chapter 5. Objectives Work and Employment – Measures 5.5 it calls for : "Ensuring a network of professional support for the provision of vocational rehabilitation services: (…), ensuring a network of vocational rehabilitation service providers; (…) encouraging the development of employers' network for providing training (…); the inclusion of non-governmental and/or disabled people's organizations in the system of support services within the framework of supported employment."

Policy provisions promoting persons-centred approach and individualised service provision

Vocational rehabilitation is a public service and it covers all of the Slovenian territory at the level of the regional units of the Employment Service. The services are delivered by teams of several members, one of them is a medical doctor, others are occupational therapists, psychologists, social workers etc. Vocational rehabilitation is provided through different services, which include: motivating activities, employment assessment, psychosocial rehabilitation, vocational guidance,

social skills development, job finding, work place analysis and accommodation, training, counselling in training and education, follow up professional support and supported employment. Vocational rehabilitation is based on a rights oriented and active approach, with person centred planning (individual rehabilitation plan) and working towards constant improvement.

Policy provisions on localised and accessible employment service provision

The vocational rehabilitation services are organized at the same regional level as the units of the Employment Service of Slovenia.

Vocational rehabilitation in Slovenia is carried out as a public service within the network of public and private providers of vocational rehabilitation. All providers must conduct services according to a national recognized and verified program.

Job seekers with disabilities can receive support from the Employment Service of Slovenia, other national agencies, private and public organizations which provide vocational training and various NGO's depending on the type of disability.

Employment support in the open labour market

From 2004, it is possible to get services for persons with disabilities in the form of supported employment. Approval by the Employment Service of Slovenia is required. On the basis of the Employment and Rehabilitation of persons with disabilities Act, the Public Guarantee, Maintenance and Disability Fund of the Republic of Slovenia has an obligation to pay supported employment services to persons with disabilities who have obtained decision on employability in supported employment and are employed in the open labour market. Supported employment services can be offered for a maximum of 30 hours per month. Another possibility is to get supported employment services from the network of the vocational rehabilitation providers. Support could include expert and technical support, awareness raising activities/information activities, counselling, training, personal assistance, follow-up support at the workplace and assessment. A wage subsidy is also possible. The person has to be trained, motivated and must have an individual support plan.

Candidates are referred by the Employment Service of Slovenia, the key Slovenian labour market institution, to vocational rehabilitation programmes and can stay in the programmes up to 2 years. They can also referred by employers, by the Pension and Disability Insurance Institute of Slovenia or in some case by third party. The legal ground for vocational rehabilitation in Slovenia is the Vocational Rehabilitation and Employment of Persons with Disabilities Act (2004) with its implementing regulations. Vocational rehabilitation is defined as the right of the persons with disabilities to certain vocational rehabilitation services specified in a rehabilitation plan. All persons with disabilities assessed as level 2 or 3 (see above) can receive vocational rehabilitation and training. In order to empower and prepare candidates for work they receive various types of training and work experience and in different organizations, depending on what is most suitable for them.

In Slovenia there are some people with disabilities in self-employment but the estimated percentage in the population is rather low. Like any other job seekers, job seekers with disabilities can benefit from a national grand scheme for encouraging self-employment. If the criteria are met they are entitled to different types of support for people with disabilities (for themselves or for the people with disabilities they employ): wage subsidies up to 70%, compensation for adapting jobs including adaptation of premises, purchasing equipment and providing a specific training; exemption from payment of contributions for pension and disability insurance of employees with disabilities; bonuses for exceeding the quota and annual awards to employers for good practices.

Protections and privileges:

- Employees with disabilities are protected against dismissal (on the basis of the disability) as part of the anti-discrimination legislation.
- Adaptation of the working hours can be part of the individual rehabilitation plan. There is possibility of financial compensation for adapting jobs including adaptation of the premises and purchasing equipment, wage subsidy, supported employment services, award for exceeding the quota, and exemptions from tax and social security contributions (for employers).

Employment support through Social enterprises or social cooperatives

There are social cooperatives in Slovenia but (apart from the employment centres, see next point) it is not clear how many people with disabilities are among the workforce.

Employment support through sheltered work

The Vocational Rehabilitation and Employment of Persons with Disabilities Act defines sheltered work as the employment of persons with disabilities in a flexible working environment adapted to the needs and working capacities of employees who are not able to be employed in a regular job. The sheltered work sector is a network of specific centres across the country (just over 463 employees with disabilities in 43 Employment Centres in 2015). Workers have a labour contract which includes among general provisions also support, training and counselling according to the needs of the disabled employee. Employment centres are very much dependent on the quota system, because businesses, which do not employ enough persons with a disability, can buy out of that obligation by providing work to employment centres.

Incentives for persons with NCDs to participate in activation programmes

Partial disability pensions are possible for working people.

Financial incentives for employers to recruit/retain persons with NCDs

There are:

- wage subsidies for employers who hire people with disabilities (5% to 30% of the minimum wage in the open labour market, 30% to 70% in the sheltered employment; depending on the level of disability);
- payment of the workplace adaptation;
- payment of supported employment services;
- exceptions of tax and social contributions;
- award for exceeding the quota;
- annual award employers for good practices.

Non-financial incentives for employers to recruit/retain persons with NCDs

All non-financial incentives are aimed at all people with disabilities, for example supported employment services provided by the vocational rehabilitation providers, different training programmes, education etc.

Duties of persons with NCDs

Duties of persons with NCDs are the same as the duties of all persons with disabilities. There are no obligations to undergo vocational rehabilitation or any others employment activation programmes before being eligible for disability benefits.

For obtaining the status of person with disabilities under the Vocational Rehabilitation and Employment of Persons with Disability Act, persons with disability can be required to actively participate in vocational rehabilitation programme.

Duties of employers

According to the Vocational Rehabilitation and Employment of Persons with Disabilities Act, private and public companies and organizations with a work force of more than 20 employees must hire between 2% and 6 % of employees with disabilities, depending on the business branch. Employers who fail to meet the quota criteria are obliged to a monthly payment of 70% of the minimum wage for each person with disabilities that should be employed by the company. That money goes into a special Fund which in turn provides financial support and awards to employers, who exceed the quota.

General and specialised employment services for persons with NCDs

The government has not adopted any specific policy and strategy or programme, regulation or act to promote employment only/specifically for persons with chronic diseases/disorders.

Employment rights of people with disabilities in Slovenia are not granted in one act that would serve as umbrella legislation for employment of persons with disabilities. The government has adopted

several documents to promote employment persons with disability, regardless of the type of disability, thus also covering employment of people with chronic diseases/disorders.

Besides programmes for promoting employment of people with disabilities, there are other psychosocial programmes aimed at promoting inclusion and development on other life areas, but these programmes are not directly aimed at promoting employment of people with disables. The significance of those programmes lies in raising self-esteem and quality of life in general, which can in turn influence employment possibilities.

The target group is all people with disability (people with mental health, physical, intellectual and/or sensory impairments), regardless of the disability type.

The strategies, legislation and programmes listed below are long-term oriented.

There are some (voluntary) programmes in companies, such as a special programme for return to work of blue-collar workers people with chronic illness in a company for freeway maintenance and toll collection (DARS).

4.4. Mediterranean model

4.4.1. Greece

Main legislative frameworks on chronic diseases, mental health and employment

In general, specific chronic disease categories are covered by the broader legislation on persons with disabilities and/or persons belonging to vulnerable groups. Particularly, the Law 2643/1998 outlines protective measures of employment for the various categories including persons with at least 50% disability, who have limited capability for professional employment due to any chronic physical or mental or psychological condition or deficiency. The national law on social economy and social cooperatives provides a framework for Limited Liability Social Cooperatives, which involves provisions concerning employment integration for persons with mental health issues (Law 2716/1999 on development and modernisation of mental health services; Law 4019/2011 on Social Economy and Social Entrepreneurship). Persons with chronic disease with disability rate over 67% are also singled out as a distinct group under the legislation on post-secondary education, as they could pursue their studies in higher educational institutions without entrance exams (5% per faculty or department).

Main legislative frameworks on disability and employment

- Law 2646/1998 - Organization and Operation of the National System of Social Care;
- Law 2643/1998 – Ensuring the employment of specific categories (establishment of the quota system for compulsory employment of persons with a minimum disability degree of 50% in private and public sector);
- Law 2716/1999 – Development and Modernization of mental health services (provisions for establishing Limited Liability Social Cooperatives);
- Law 3304/2005 on the Implementation of the principle of equal treatment regardless of race or national origin, religion or other beliefs, disability, age or sexual orientation. Article 9 of the law, however, does not consider as discrimination when the nature of specific tasks do not allow for the employment of disabled people, given the implementation of reasonable accommodation;
- Law 4019/2011 on Social Economy and Social Entrepreneurship;
- Law 4074/2012 – Ratification of the UN Convention on the Rights of Persons with Disabilities and its Optional Protocol;
- National Strategic Framework for Social Inclusion (2015-2020).

Policy provisions on mainstream and specialist employment programmes

The OAED is a main body in Greece for mainstream employment services. It has six specialised offices across the country, for "Special Social Groups," which are in charge of services for groups considered vulnerable in terms of inclusion in the labour market. Unemployed persons with chronic diseases with a certified disability degree of over 50% can be included in this group. Specifically, the services of these specialised offices include career orientation guidance, psychosocial support and referral to mainstream employment and vocational training programmes.

Policy provisions on access to employment support

Disability certification plays an important role in the access to employment integration support since only persons with a certified disability rate of over 50% can access such support. In addition, when a

person is recognised as “incapable of any gainful work,” she/he cannot access any work and work integration activities.

An authorized health committee, Disability Certification Centre, assesses health conditions of people with disabilities or chronic diseases and determines the degree and the severity of their impairment. The assessment is based on the Specific Disability Criteria (Disability Percentage Determination Integrated Table), according to a Joint Ministerial Decision (Government Gazette B’ 1506/4-5-2012). It is important to note that the assessment concerns the health or the severity of the disability of the person but not necessarily the person’s capacity to work. Even though the terms “capable to work” or “incapable to work” exist in the assessment procedure, they are not widely used, while the term “reduced capacity” does not exist. There are, however, terms such as “incapable of any gainful work” or (in rare cases) “incapable for particular types of work” that can be in use.

According to the Ministry of Health, there is a plan for changing the existing system through the implementation of the Disability Card, based on the International Classification of Functioning, Disability and Health (ICF), which means that the disability assessment will not be limited to a health condition or an impairment, but will also include activity limitations and participation restrictions, taking into account environmental and contextual factors. Despite a comprehensive study on the implementation of this plan since 2006, and the supporting Information System since 2008, the implementation of the plan is pending. The state is attempting to put the plan into practice, making the appropriate interventions in all required fields.

Policy provisions promoting stakeholder cooperation and integration of services

The coordination among different sectors and the continuity of support have not been adequately achieved in national employment integration services, which constitutes a significant obstacle to labour market participation. Knowledge and information sharing among civil servants, social workers and health professionals is poor. However, some previously implemented programmes such as TOPEKO (Local Actions for Social Integration of Vulnerable Groups), co-funded by the ESF and the Ministry of Social Security and Labour Welfare, attempted to adopt an integrated service approach, as they combined vocational counselling, training and internship in specific and relevant subjects. TOPEKO projects were based on a partnership between private and public sector and an integrated effort of municipalities, consultancy businesses, and vocational training centres. The aim of TOPEKO

was to provide integrated support services to unemployed people from vulnerable groups who were registered at the Manpower Employment Organization (OAED).

Policy provisions promoting persons-centred approach and individualised service provision

A person-centred approach is not given a priority in the Greek system of employment activation. In fact, individual qualifications and skills may play a secondary role in job placement because the system is built in a way that persons with a recognised disability are given points based on the severity of their conditions or other factors, and those with the highest score can benefit from protection in job placement. Thus, selection for a protected job may be based not only on professional and personal competences, but on the procedural aspects of the system. Employers, in turn, cannot select an employee based mainly on the requirements for specific job vacancies, which creates further complications as they can appeal the placement and delay the recruitment.

Policy provisions on localised and accessible employment service provision

OAED has seven Regional Directorates and local PES. The latter have gone through a reform to become more integrated with the central PES. This has been done to create a unified employment service that would save time on administrative issues and provide one-stop-shops for unified employment and social insurance services.

Employment support in the open labour market

Support programmes:

- Local Actions for Social Inclusion of vulnerable groups (TOPEKO) aimed at promoting the employment of persons from vulnerable social groups (registered as unemployed with the OAED) in community businesses, self-employment, startups, or social cooperative enterprises. TOPEKO provided funding for development partnerships involving counselling, training and job placement for different categories of beneficiaries. From the total of 131 development partnerships established in the context of TOPEKO, 36 included persons with disabilities as target group, 8 also targeted persons with mental health issues and 1 included

persons with various types of chronic health conditions. The OAED was in charge of facilitating different types of training for jobseekers. For instance, through the TOPEKO programme it was possible to obtain basic professional training or to acquire new professional skills, including education in the field of entrepreneurship and social economy, and to receive psychosocial counselling and business advice. Different training programmes were possible:

- Pre-training programmes – intervention activities for vulnerable groups (e.g. professional terminology, computer skills, information on legal issues);
- Subject-specific practical training (i.e. internship);
- Certified training and lifelong learning programmes;
- Vocational training in specific educational subjects in specialized social and professional integration centres;

However, the implementation and funding of training programmes is difficult due to financial constraints. Thus, training opportunities are scarce.

- Local Plans for the Development of Employment (TOPSA) were implemented by the regions with funding from the Operational Programme on Human Resources Development (EPANAD). Each programme was tailored to the needs of local labour markets and targeted the general population, not specifically vulnerable social groups.

Protections and privileges:

- Persons with disabilities or chronic illnesses are exempted from the legislation concerning the suspension of employees in the public sector due to the disestablishment of working positions.
- In the private sector, employees with a disease that requires blood transfusions and HIV patients are entitled to an additional leave. In the public sector, employees with chronic diseases are entitled to paid sick leaves and a reduction of the working hours.
- Law 3846/2010 grants the right to “telework” and allows the conversion of a regular employment into telework.
- Employees dismissed due to a physical and/or mental disability can be re-appointed within five years from their dismissal after the assessment of the Health Committee confirming that the person has the physical and/or mental capacity to perform their duties.

- Employers are required by Article 10 of the Law 3304/2005 to take all necessary measures concerning reasonable adjustments and accessible workplaces for disabled people, as long as this does not constitute a disproportionate burden to employers. Funding for workplace adaptations is made available through ESF funds, managed by the OAED, which announces call for such funding schemes.

Lack of protection**:**

- Persons with a disability degree over 50% cannot benefit from compulsory recruitment under the quota scheme for seasonal jobs (e.g. tourism);
- The lack of specific provisions for professional reintegration hinders the return to employment, especially when the person is absent from the workplace for a long time.

Employment support through Social enterprises or social cooperatives

The main body responsible coordinating, monitoring and developing the social economy in Greece is the Special Service for Social inclusion and Social Economy (EYKEKO).

The Law 4019/2011 provides a general legal framework for social economy and social cooperative enterprises (Kinoniki Sineteristiki Epihirisi, KoinSEp) in Greece. Two categories of KoinSEp can be distinguished in relation to work integration:

- Inclusion KoinSEp – aimed at the socio-economic inclusion of vulnerable groups mainly through work integration. In such entities, at least 40% of the employees should come from social groups at risk.

- Social Care KoinSEp – aimed at providing social care services to people with disabilities or chronic illness, to children, to the elderly, etc. Although this category of KoinSEp is not directly liked to employment integration, it can be involved in the provision of counselling services to persons with disabilities and chronic conditions.

Furthermore, the Law 2716/99 provides a framework for Limited Liability Social Cooperatives (Koinonikos Sineterismos Periorismenis Efthinis, KoiSPE). These are work integration social enterprises for people with psychosocial problems, which simultaneously provide mental health services. Members of KoiSPE can include: at least 35% of persons with psychosocial disabilities, up to

45% mental health professionals, and up to 20% other organizations or natural persons. KoiSPEs are supervised by the Ministry of Health, can engage in any commercial activities and have an independent legal and tax status as limited liability businesses. The law gave them exemption from corporate taxes except the Value-added tax; however, financial support for establishing and maintaining business activities is limited and inadequate due to budgetary issues. Users in KoiSPE may earn a wage without losing their benefits. There are currently 23 KoiSPE in Greece.

Employment support through sheltered work

Although there is a legislative provision for sheltered employment (in the form of sheltered workshops), it is not implemented for persons with chronic diseases. However, sheltered workshops and occupational rehabilitation centres targeting persons with mental health issues or other types of disability are operated by non-profit organizations, day-centres supervised by public hospitals, etc. The focus on transition to the open labour market in sheltered workshops may vary depending on agencies in charge.

Incentives for persons with NCDs to participate in activation programmes

Participation in some employment programmes implemented by the OAED (e.g. programme for temporary placements in public sector within local communities or Voucher programme for unemployed to combine training and work) can provide extra points to unemployed persons with disabilities.

Under the Law 4331/2015, Article 13, persons with disabilities entitled to welfare benefits can participate in paid training programmes or in programmes promoting employment, including self-employment programmes or social cooperatives offering them regular incomes, without losing their welfare benefits. In practice, however, the payment of benefits has been problematic due to budgetary constraints.

Greek laws clearly state that persons with disabilities eligible for welfare benefits can participate in social cooperative enterprises as workers, without losing their benefits. However, there are no clear specifications about what happens to disability pensions. Therefore, this can be considered as a disincentive for many persons who have a lower rate of disability and are eligible for disability

pensions. This can create confusion and result in the fact that people decide not to work and continue receiving disability benefits.

Financial incentives for employers to recruit/retain persons with NCDs

There are no wage subsidies currently paid to employers for hiring persons with disabilities, due to the impacts of the crisis. There are still, however, subsidies in terms of employers' insurance costs.

- A 3-year special programme is implemented by the OAED with an aim of promoting the recruitment of the unemployed from special vulnerable groups through a subsidy paid to an employer, corresponding to the total social insurance contributions' amount. The programme aims at recruiting 2,300 unemployed people from vulnerable social groups (2.200 full-time jobs, corresponding to 2.080 positions for registered unemployed persons with official disability levels of over 50%).
- Another subsidy programme is also in place for 800 new entrepreneurs with disabilities, ex-drug addicts and ex-convicts aged 18-64 years (600 beneficiaries were persons with disabilities).
- Supplementary to the above mentioned programmes, subsidies are also available for 50 jobs involving the ergonomic arrangement of workplaces for people with disabilities.

These subsidy programmes are limited in time and it is unclear whether the completion of the particular programme will entail the absence of financial support to employers or whether the implementation of another similar programme will follow.

Non-financial incentives for employers to recruit/retain persons with NCDs

In 2014, public consultation for the national action plan for corporate social responsibility was completed, but only a draft of the National strategy of Greece for Corporate Social Responsibility is currently available online. However, the national Corporate Social Responsibility network (CSR-Hellas) representing Greece at European level has been established. More than 60 companies are members of the network. The network publishes and disseminates both good practices/actions and Social Responsibility evaluation reports of specific members (not an overall evaluation report).

Duties of persons with NCDs

There are no obligations to undergo employment activation programmes before being eligible for disability benefits.

Duties of employers

There is a legal obligation for private companies to ensure that 8% of their staff consists of persons from vulnerable social groups protected by law. Persons with a disability degree of over 50% may fall under the quota (2%). The law concerns companies that have more than 50 persons in staff and have positive financial results in the previous two years.

In the public sector, quota for recruiting persons from vulnerable social groups equals to 5%. Persons with disabilities account for 3/8 of this quota amount. In private and public sectors, persons have to ascertain that they fall under the category of protected employees from vulnerable groups. Public sector entities (public enterprises and public institutions) private entities (private law organizations) belonging to the State or to the local authorities and organisations providing services in the public interest are obliged to cover 10% of their staff with "protected persons" corresponding to 3% for persons with disabilities. Additionally, public services, local authorities and public bodies shall employ "protected persons" to cover 20% of specific jobs (e.g. guards, porters, gardeners, cleaners), but there is no provision for the precise percentage of positions obtained by persons with disabilities.

Eligible persons for participating in compulsory recruitment through the quota system have to be registered as unemployed with the OAED and to have a certified disability of over 50%. Special requirements apply to certain categories of professions (e.g. lawyers, educators). Furthermore, according to Law 3528/2007 and Law 4210/2013, recruitment in the public sector requires that the health condition of the prospective employee allows her/him to perform the required duties. A lack of physical abilities does not prevent the placement, provided that the employee has appropriate and justified technical support in order to perform the duties related to the position. The health and physical fitness of candidate employees for related positions are certified by a physician or a general practitioner and a psychiatrist, based on a referral document, which describes the tasks related to job posts. Issues arising from the above mentioned laws concern: (a) the reduced coverage of

expenses from the Insurance Institute in order to secure the necessary devices/adaptations, (b) existing infrastructure in the workplaces, (c) adequacy of the physician/general practitioner and the psychiatrist to assess accurately the requirements and duties of the positions and therefore the suitability of disabled people for specific jobs.

Due to the fact that compulsory employment applies to companies that are profitable, not many companies actually were subjected to quota fulfilment due to the financial crisis. In addition, an inefficient implementation of the law leads to non-compliance of companies with the quotas, especially in the private sector.

Under the Law 2643/1998, the government is supposed to issue a call for job vacancies and job placements for socially vulnerable groups on yearly basis; however, only a few have been issued since 1998.

General and specialised employment services for persons with NCDs

Also, information about available working positions or employment programmes can be provided by the National Confederation of Persons with Disabilities or other patients' associations. Different organisations can provide such services to their target groups. For example:

- Panhellenic Union for the Psychosocial Rehabilitation and Work Integration provides support in finding and maintaining employment to people with mental health issues.
- Association "Hellenic Pulmonary Hypertension" provides services for professional (re-)integration of persons with respiratory diseases.
- Actions for raising awareness and fighting stigma usually take place as parts of broader strategies/programmes or as initiatives undertaken by NGOs and patient associations. They usually refer to specific health conditions (e.g. breast cancer, depression) and primary health care (not focusing on employment issues).
- Patients' organisations also provide information support, guidance and consulting about the rights of persons with health conditions and about available supports. The National Confederation of People with disabilities, for instance provides information to its members about compulsory recruitment, information about labour market integration, etc. and helps them in filing complaints with the Ombudsman (e.g. complaints regarding discrimination in recruitment, reasonable accommodation or other).

In general, the situation in Greece remains problematic with regards to the employment of persons with disabilities and chronic diseases due to economic difficulties. The lack of state support goes hand in hand with a high reliance on family support and informal arrangement allowing individuals to keep their jobs (e.g. appointments and consultations with doctors over the phone so that patients do not have to leave their work). This is due to that fact that workers are afraid of losing their jobs and place a higher priority for work rather than health.

4.4.2. Italy

Main legislative frameworks on chronic diseases, mental health and employment

In Italy, there is no legal definition of chronic diseases. In some cases, upon meeting requirements provided by different disability laws, persons with chronic diseases are covered by policies with regard to professional integration for persons with disabilities. For some diseases, in particular cancer, specific dispositions exist: Legislative Decree 276/2003, Article 46 gives the right to switch from full time to part time, until the improvement of health conditions, for full-time employees with cancer. Ministerial circular 1, April 30, 2009 provides a possibility of teleworking to persons with cancer in the public sector. Law 247/2007 recognizes the priority to switch from full time to part time to caregivers.

Also for diabetes a law guarantees the right of not discrimination in the workplace: the Law 115/87 on Rules for the prevention and treatment of diabetes mellitus, Articles 1 and 2 refer to the responsibility of the authorities of autonomous provinces of Trento and Bolzano to facilitate the integration of people with diabetes in school activities, sports and labour. Article 8 -1 of the same law states that diabetes without disabling complications should not impede the issuance of the physical aptitude certificate that allows access to public and private places of work.

A recent law decree extends the dispositions for cancer to all the chronic degenerative diseases: the Legislative Decree 81/2015[59] takes into consideration the needs of persons with chronic diseases and in particular serious chronic degenerative diseases. Article 8 of the Decree gives the right to persons with above-mentioned diseases to transform their full-time job to part-time if their conditions cause a reduced work capacity, and recognizes also to caregivers the priority to switch

---

59 http://www.unive.it/pag/fileadmin/user_upload/ateneo/job/documenti/Stage_e_placement/D.Lgs._n.81_2015.pdf

from full time to part time. At employees' request, the part-time can be transformed back to full-time.

The Decree of 11 January 2016 contains specific provisions with regard to "availability times" in case of serious illnesses that require life-saving therapies, or in favour of workers suffering from diseases related to an invalidity recognised.

Main legislative frameworks on disability and employment

- Legislative Decree 509/1988 regarding the recognition of civil invalidity.
- Law 104/92 on the assistance, social integration and the rights of persons with disabilities includes general rules on socio-economic inclusion of persons with disabilities, including provisions for reasonable accommodation.
- Law 68/1999 provides a framework for employment and job integration of people with disabilities.
- Legislative Decree 216/2003, Implementation of Directive 2000/78/EC on equal treatment in employment and occupation. (The Legislative Decree 76/2013 (Art. 4. b) added a paragraph to art. 3 of Legislative Decree 216/2003 regulating the reasonable accommodation).
- Partnership Agreement 2014-2020 with the European Commission sets the strategy for the optimal use of European Structural and Investment Funds. The agreement includes a set of actions for increasing the participation of the most vulnerable groups in the labour market.
- Legislative Decree 151/2015 is part of Italy's reform of the labour laws. Adopted on September 14, 2015, Article 1 of the Decree states that guidelines should be developed on targeted employment of persons with disabilities on the basis of principles such as reasonable accommodation and identification of good practices.
- Ratified the UNCRPD on 15 May 2009.

Policy provisions on mainstream and specialist employment programmes

In Italy, employment services to persons with reduced working capacity are provided at regional, provincial and local levels. There are differences between localities and there is no universal approach throughout the country.

Regional employment agencies, which provide help and advice about work-related problems and self-employment, are operated by the Ministry of Labour and Social Welfare and there are also local employment centres in municipalities. For persons with certified disabilities of over 45%, provincial authorities are responsible for their work placement. For the province of Milan, it is the Service for the employment of people with disabilities (Servizio Occupazione Disabili) that handles the list of people who may benefit from mandatory work placement.

For each registered person, a technical committee from the employment services creates a file where relevant information, such as qualifications, skills, personal preferences, and the nature and degree of disability, are noted down. The committee, which consists of experts from social, healthcare and employment services, also analyzes the tasks that can be performed and workplace adaptations that may be required. The targeted employment services are responsible for matching job requests with offers available in the market.

In the Lombardy Region, regional authorities have established a register of organizations that can provide job placement and integration support and also interact between candidates and companies. These service providers create personalized services such as counselling, guidance, help in creating CVs, interview preparation, etc. The provinces support the integration of persons with reduced work capacity through various projects and cooperation with third parties.

Policy provisions on access to employment support

The Law 68/1999 includes a provision on targeted placement, which proposes an assessment of the ability of persons with disabilities in work placement.

The Partnership Agreement 2014-2020 signed between Italy and the European Commission includes an action to promote the dissemination and the customization of the ICF model at local level.

The Decree 151/2015, which aims to modify the Law 68/1999, states that a bio-psycho-social approach is needed in evaluating disability and that targeted employment services should also take into account barriers and enabling factors found in the working environmental while defining criteria for employment projects.

In practice, however, the evaluation of the degree of disability is often based on a medical approach with a focus on impairments. In fact, in order to access the benefits, the person must be recognized a certain percentage of disability.

Policy provisions promoting stakeholder cooperation and integration of services

Article 1 of Legislative Decree 151/2015 states that guidelines should be developed on targeted employment of persons with disabilities on the basis of various principles, including the following two:

- Integration of social, health, education and vocational rehabilitation services as well as the services of the National Institute for insurance against accidents at work (INAIL);
- Territorial agreements among trade unions, employers, the social cooperatives, associations of persons with disabilities and their families, as well as with organizations from other sectors.

The Partnership Agreement 2014-2020 signed between Italy and the European Commission includes an action that calls for multi-disciplinary and personalised interventions encouraging the inclusion of persons with disabilities in the labour market (Action 9.2.1).

Policy provisions promoting persons-centred approach and individualised service provision

Individualisation of the employment support for persons with disabilities in Italy is emphasized through the targeted employment approach. One of the principles of targeted employment mentioned in Article 1 of the Decree 151/2015 is individualisation of employment projects for persons with disabilities.

According to modifications made by the Decree 151/2015 to Article 8 of the Law 68/1999, unemployed persons with disabilities can register with targeted employment services, which are coordinated by local authorities. For each registered person, a technical committee from the employment services creates a file where relevant information, such as qualifications, skills, personal preferences, and the nature and degree of disability, are noted down. The committee, which consists of experts from social, healthcare and employment services, also analyzes the tasks that can

be performed and workplace adaptations that may be required. The targeted employment services are responsible for matching job requests with offers available in the market.

Policy provisions on localised and accessible employment service provision

In Italy, there is a national law that provides a general legislative framework for the employment reintegration of persons with disabilities (Law 68/1999). However, Italian regions then issue their own regulations to implement the law. They decide on how these provisions are implemented and coordinate all related activities. The law encourages cooperation among stakeholders at local level. Local job centres work in close cooperation with municipalities, for example, CELAV (Centre for Job Coaching) and the Milan Town Council.

Provision of professional (re-)integration policies in Italy is handled and funded by provincial authorities. The authorities, however, do not provide the services directly. They regularly launch calls for tenders among service providers who can contribute to the implementation of active labour policies. These service providers can be non-profit agencies (e.g. social cooperatives), for-profit labour agencies (e.g. private human resource consultancy firms such as Adecco or Man Power), or local public employment agencies. In the Lombardy Region, for example, regional authorities have established a register of organizations that can provide job placement and integration, support of people in their workplace and also interact between people concerned and companies.

Employment support in the open labour market

At national level, there are no supported employment programmes; however there are regional and provincial level initiatives that can relate to the definition of supported employment (e.g. support in accessing and maintaining to employment in open labour market, support to employee before, during and after obtaining a job, job coaching, support to employers, focus on abilities). The targeted employment services that are present in Italy at local levels do pursue active labour market policies.

All local authorities are responsible for developing their own programmes facilitating the employment of persons with disabilities. There are differences between various provinces and municipalities in the way these programmes work. Therefore, it is hard to give a generalized national overview of employment support programmes. In Milan, for example, municipal authorities have

introduced the EMERGO Plan, a program for persons with disabilities aimed at facilitating their labour market participation and maintenance of work through support services and activities. The Plan provides funding for different tools enhancing the employment of disabled persons. The following two forms of tools exist:

- DOTE - a funding scheme, which is in fact a package of services formally awarded to individuals with disabilities to support their training or other labour market (re-)integration activities. DOTE is used to support job placement (Dote inserimento), but it can be also used to help employed persons maintain their jobs (Dote sostegno). DOTE is technically an allowance in a form of services for individuals in need of employment support. The funding goes directly to service providers that apply for DOTE on behalf of individuals.
- Funding of projects that envisage the involvement of companies rather than individuals and that target (re-)integration activities of general nature. The projects may be aimed at awareness raising, workplace adaptation, guidance, advice, training for staff, counselling for managers working with persons with disabilities, etc. For example, a blind persons' institute may offer to develop specialised practical guidelines or tools for a company that employs persons with visual impairments.

The EMERGO Plan publishes different calls on yearly basis, targeting different categories of persons, different types of disabilities, different measures, etc. Various service providers apply for these calls, either with an aim to provide personalised job placement or maintenance support through DOTE schemes to persons in disadvantaged employment situation or to provide support to employers through various projects. Both employers and employees can choose service providers to support them in workplace integration.

Another regional initiative from the province of Verona is the 'SIL 22' job integration service, which aims to promote the employment of people with disabilities through encouraging cooperation between health and social (employment) services. The services offered are information provision, vocational training, career planning, case management and advocacy, preparation of users for jobs, job matching and placement, and post-placement support.

The employment plan of Milan covers employment supports at different stages of integration (before, during, and after recruitment). The focus is, however, the highest on job placement, rather than on job maintenance. Indeed, Italian companies are subject to quotas, an obligatory recruitment of persons with disabilities. Thus there is a strong system in place for targeted employment, according to which persons with disabilities are included in a list from which employers are obliged

to recruit workers. There are also measures intended to keeping persons with disabilities or ill-health at work, but they are not too extensive. They are mainly aimed at making work arrangements more flexible. For example, under the Law 68/99, persons with disabilities are entitled to a change of duties in cases when they can no longer to perform their tasks. There are also provisions that persons with disabilities can choose the nearest office or avoid night shifts.

In general, rules for recruitment and dismissal of persons with chronic diseases are the same as for the rest of the labour force. In this sense, there are no additional protections granted by the legislation. Instead for workers with disability specific rules exist: unjustified dismissal of a disabled worker is considered discriminatory and the employee is entitled to reinstatement in the workplace. (Chronic diseases are not included among the prohibited causes of discrimination set out in Article. 15 of the Workers Statute). Article 41 (paragraph 2) of Legislative Decree 81/2008 (Law on safety at work) states in case of an absence due to ill health lasting more than sixty consecutive days, it is necessary to check the suitability of the worker to perform her/his task by a medical examination. Following the medical check, a physician may deliver a written opinion about the fitness of a worker to a specific job. In case of a temporary unfitness, a timeframe needs to be provided to the employer and the employee.

According to paragraph 42 of the Decree 81/08, when the physician determines the unfitness to a specific task, the employer must assign the worker, where possible, to an equivalent task or, if it is not possible, to lower tasks, ensuring the previous level of income.

A dismissal of an employee due to a reduced ability to work as a result of a chronic illness requires a proof that the employer was not able to assign the employee to other tasks compatible with her/his health condition and with the good performance of company (This practice has been defined by law as an "obligation to repechage" (See. Cass. Civ, n. 7755, August 7, 1998).

There are no specific programmes targeted at the reintegration after long term absence. However, there are some local level projects that may provide such services.

In terms of measures supporting employers, the Partnership Agreement 2014-2020 signed between Italy and the European Commission includes Action 9.2.4, which calls for supporting enterprises in recruitment of persons with disabilities, adaptation of the workplace, promotion of diversity in the workplace [awareness raising about diversity management and career path management of workers with disabilities, etc.], and development and dissemination of assistive technologies facilitating the employment for persons with disabilities.

Employment support through Social enterprises or social cooperatives

Social cooperatives have been legally recognized in Italy in 1991 through the Law 381/91[60] and defined as entities intended to pursue the general interest of the community to promote the social integration of citizens by engaging in employment integration activities for disadvantaged groups and by providing social, healthcare or educational services. Social cooperatives are governed by the members of the cooperatives consisting of providers and users of social services.

In Italy, there are two types of social cooperative:

- Type A cooperative that engage in the provision of social, health and educational services. In particular, the services provided are:
  - Integrated social health home care,
  - Management and service provision for the elderly, psychiatric patients and persons with disabilities,
  - Management of summer camps and after-school care for children.
- Type B cooperatives that carry out various activities, including agricultural, industrial, commercial or other activities, aimed at the employment of disadvantaged groups of population and at development of their professional skills. In particular, the activities are:
  - Cleaning,
  - Environmental cleaning,
  - Maintenance of green construction and maintenance paths,
  - Forestry work,
  - Nursery,
  - Building maintenance, electrical and hydraulic,
  - Bell boy,
  - Housing buildings.

Generally (within type B social cooperatives), there is no specialist staff to support employees with disabilities in the workplace. This task is normally carried out by non-disabled colleagues or volunteers.

[60] http://www.handylex.org/stato/l081191.shtml

Employment support through sheltered work

Sheltered type of work in Italy is particularly spread among persons with psychiatric disorders. Following types of sheltered work exists in the country: sheltered internships, sheltered workshops, occupational therapy centres.

Incentives for persons with NCDs to participate in activation programmes

In case of a partial or a total reduction in work capacity, Italian employed or independent workers can claim the following two benefits:

- Ordinary disability allowance (assegno ordinario di invalidità) - covers insured workers whose working capacity is reduced to less than a third due to a physical or mental impairment. Beneficiaries of this allowance can combine this benefit with their income from work.
- Disability pension (pensione di inabilità) - covers insured workers with a permanent and total disability and is not compatible with any kind of income. Beneficiaries of this benefit are acknowledged to be totally and permanently incapable of carrying out any kind of work activity.

The possibility to keep the ordinary disability allowance while working can be seen as an activation incentive for persons with chronic conditions.

Financial incentives for employers to recruit/retain persons with NCDs

Article 13 of the Law 68/1999 on economic incentives to hiring persons with disabilities has been modified in 2015 by Article 10 of the Decree 151/2015 to define the following incentives for private and public employers in hiring:

- 70% off the monthly gross taxable income for social security purposes for a thirty-six month period for each permanent contract offered to a person with reduced work capacity exceeding 79% or with physical impairments as per lists attached to the legislation on war pensions.

- 35% off the monthly gross taxable income for social security purposes for a thirty-six month period for each permanent contract offered to a person with a reduced work capacity exceeding 79% or with physical impairments as per lists attached to the legislation on war pensions.
- A 70% off the monthly gross taxable income for social security purposes for a period of 60 months for each permanent or temporary contract (of at least 12 months) offered to a person with a learning or mental disability resulting in a reduced work capacity superior to 45%.

Non-financial incentives for employers to recruit/retain persons with NCDs

In one example of such incentivising, the Italian Multiple Sclerosis Society has been working with national trade unions, Confindustria (representative organisation of manufacturing companies) and AIDP (Italian Association for Human Resources Management), on raising awareness among enterprises about the benefits of having a diverse labour force. The aim is to explain that management of chronic disease is not a financial burden, but, on the contrary, it has an added value for the companies: if well handled, job reintegration programmes can help organisations retain exiting workers, in particular, senior employees who have valuable experiences and qualifications that could be of great value.

Under the Law 68/1999, companies in Italy are obliged to hire persons with disabilities; however, they can be also incentivised to engage in work integration programmes as part of their corporate policy on social responsibility. For example, in Milan, companies are offered an opportunity to be included in the list of ethical companies if they participate in the Regional Innovative Programme for Mental Health (Programma Innovativo Regionale per la salute mentale - PIR), which supports the integration of persons with mental health conditions in employment.

Duties of persons with NCDs

In Italy, the issuance of disability benefits is not conditional on participation in employment rehabilitation programs. Thus, there is no obligation from the side of individuals to undergo vocational rehabilitation before qualifying for benefits.

Duties of employers

According to the Law 68/1999, every company with more than 15 employees is obliged to hire at least one person with a certified disability; companies with 30-50 employees are obliged to hire two persons with a certified; companies with over 50 employees are obliged to ensure that at least 7% of their workforce consists of persons with certified disability. This law is implemented in each region of Italy with different regulations according to local experiences.

Companies sign an agreement with the province where they are located. This is especially useful for large companies that must hire a great number of people with disability. Every year the province concerned verifies the percentage of staff of a company; in January companies have to produce a report with the number of their staff and that of persons with disabilities. They draw a plan to achieve the mandatory number of people with disability (e.g. a company with 1000 employees has to hire 70 people, and they can do so in a number of years, in agreement with the province authorities).

Provinces monitor the application of the law and can impose financial penalties to companies that don't comply with it. Several interventions of labour policy supporting integration in Lombardy Region are funded through the penalties collected. Companies operating in some fields can be exempted from complying with the law, but they still have to contribute to these funds.

Law 68/1999 covers all forms of disabilities and is not specific for chronic conditions. It establishes that people with a certified disability of over 45% can be included in the social security lists of hiring quota at province level.

According to the Legislative Decree 76/2013 (Article 4 B), employers should provide reasonable accommodations at workplace, as defined in the UNCRPD, in order to guarantee equal treatment for persons with disabilities.

Under the Decree 81/08, employers are obliged to assign the employer, where possible, to an equivalent task or, if it is not possible, to lower tasks, ensuring the previous level of income.

General and specialised employment services for persons with NCDs

In Italy, services are implemented at local level; therefore a wide range of employment services exists throughout the Italian territory. Some examples are listed below:

- CELAV (Centre for Job Coaching) (specific for the Milan municipality) provides job placement services for disadvantaged categories of people, such as people with mental or other disabilities, persons at risk of poverty, refugees, persons with drug addiction problems, etc. The services of CELAV include orientation, training, career counselling and guidance. The centre is focused at active labour policies and does not provide financial benefits; instead, it develops individual projects with the persons concerned and with the participation of local social services that point out critical situations in need of intervention. CELAV is co-managed by the Milan Town Council and the cooperative A&I Onlus (Accoglienza e Integrazione).
- Another example is the Regional Innovative Programme for Mental Health (Programma Innovativo Regionale per la salute mentale - PIR). The programme is part of a project involving the region of Lombardy that works on developing a network system that addresses the needs of mental health patients in their integration path to work, as well as the needs of their families and the needs of employers. The objective of the programme is to improve the accessibility and usability of services provided in Milan through the implementation of an organizational model that facilitates interactions between entities. These connections between entities are supported by a professional, called multi-membership coach (Coach Multi appartenenza), through inter-institutional agreements. The programme run the website Inter@lavoro (http://www.integralavoro.net/joomla/) which includes various services to employers and candidates through the analysis of business needs and the work environment, job matching, interviewing of candidates, and constant monitoring of the candidates professional career.

  The project involves the following services to employers:

  - Selection, training and placement of a candidate
  - Advice and technical support to facilitate the fulfilment of the quota obligation to employ persons with disabilities.
  - Permanent support to the company in the mediation with the worker,

  Services offered to candidates include:

  - enhancement of personal resources in relation to the work context;
  - career coaching aimed at work integration;
  - Ongoing dialogue with organizations, associations, foundations and companies dealing with health and work.

The programme includes specific Labour Training Groups, which are designed to help mental health patients acquire specific skills to better integrate at work. Some of such groups include:

- "Vediamoci Chiaro" – a training to develop and strengthen the capacities of candidates by meeting with industry professionals.
- "Pensa lavoro" – training to develop and strengthen social skills and to promote the reflection on work experience and motivation.
- "Laboratorio teatrale" – is a theatre workshop to develop and strengthen adaptability to environmental changes and in changes in the way candidates see themselves.

- The Italian Multiple Sclerosis Society (AISM) carries out work on empowering persons with multiple sclerosis by providing information about supports and measures that can help them in their career paths. Informed patients can in turn demand directly from their doctors the provision of guidelines on employment and insist on an adequate evaluation of their skills and ability to work. Thus, educating persons with ill health about possible employment support strategies enables them to request such support.
- The Italian Multiple Sclerosis Society also collaborates with professionals working in employment services in order to help them develop new competences. In particular, it worked with the employment service of Genova and provided training on how to manage and follow up on the inclusion of persons with ill-health at the workplace as well as on how to be a mediator between employers and jobseekers.

4.4.3. Spain

Main legislative frameworks on chronic diseases, mental health and employment

There are no legislative frameworks specifically targeting the employment activation of persons with chronic diseases. Such measures are covered by the broader legislation for persons with disabilities.

Main legislative frameworks on disability and employment

- Law 13/1982 on Social integration of persons with disabilities;
- Royal Decree No. 1451/1983 on Selective employment and measures to promote employment for disabled workers;

- Law 51/2003 on Equal opportunities, non-discrimination and universal accessibility for people with disabilities;
- Law 43/2006 on Improving growth and employment (measures to encourage hiring of disabled employees on permanent contracts);
- Royal Decree No. 870/2007 on Rules for the Supported Employment Programme, as means to achieve employment of persons with disabilities in the regular labour market;
- Law 35/2010 on Urgent measures to reform the labour market;
- Royal Legislative Decree No. 1/2013 approving the revised text of the General Law on rights of persons with disabilities and their social inclusion;
- Disability Action Plan Strategy 2014-2016. Ministry of Health, Social Services and Equality.

Policy provisions on mainstream and specialist employment programmes

Employment advice and support services to persons with disabilities in mainstream employment are regulated by the Public Service of State Employment (Servicio Público de Empleo Estatal, SEPE). Local offices of PES are found in all provinces. Persons with disabilities can have access to special employment centres and supported employment through these services.

Policy provisions on access to employment support

Work adaption is available for persons with NCDs, depending on decisions of occupational physicians in companies. For job seekers, however, disability is a prerequisite for additional support.

The degree of disability (expressed as a percentage) both permanent and temporary, is evaluated through the medical board, in accordance with the Royal Decree 1971/1999 of 23 December on the procedure for recognition, declaration and qualification of the degree of disability. Under this law, the assessment is based both on the impairment and, where appropriate, on social and individual factors, education level, work experience and other. The implementation of the law on the evaluation of disability is a responsibility of relevant bodies of Spain's autonomous communities. For example, in Catalonia, the assessment can be conducted by the Catalan Institute of Medical Evaluation (Institut Català d'Avaluacions Mèdiques, ICAM), which issues an opinion that is further

examined by the Disability Evaluation Commission (National Institute of Social Security, INSS). The Institute assesses the claimant's work ability.

Policy provisions promoting stakeholder cooperation and integration of services

In Spain, there is a relatively well-developed cooperation between healthcare and social service systems. Cooperation between these systems allows for a continuity of care. Health centres and social care workers can refer their patients who express motivation to work to vocational rehabilitation and job placement services.

Municipal job placement services, for example, can have access to clinic histories of their users. In addition, social welfare services put job placement services in contact with social security, welfare and family services, so that employment services have information about benefits. Regular meetings are held between health centres and job placement services. Such cooperation is especially strong between mental health centres and job placement services specialised in working with persons with mental health problems.

Policy provisions promoting persons-centred approach and individualised service provision

The Royal Decree No. 870/2007 establishes rules on supported employment for persons with disabilities, which hold that supported employment entails individualized guidance and support in the workplace by specialized job coaches to facilitate the social and work integration of disabled workers in the open labour.

In mental health, the IPS programme is used by job placement intermediaries specialised on working with persons with mental health problems. IPS is based on the idea of individualised planning and attention. Individual projects are created in cooperation between employment supervisors and users. Monitoring is done before, during and after job placement.

Policy provisions on localised and accessible employment service provision

In Spain, national legislation has given authorisation for non-profit employment intermediation agencies to facilitate the integration of persons from disadvantaged groups in employment. There is a wide range of private organisation, to a large extent non-profit entities, that render specialised activation and training services. The services of entities are instrumental for political objectives related to the promotion of the employment of persons with disabilities. Therefore, they receive funding from regional and national budget. There has been a reform in Spain that introduced an outcome-based allocation of funding for employment integration projects. This reform has an objective to enforce impact-oriented competition between service providers.

The emphasis is placed on giving regional autonomy in delivering active labour market policies. There is a common national strategy for employment activation, but regions have a freedom to choose different measures and systems to achieve the common objectives. Thus, placement and activation services are decentralised to regional level. However, local level activities remain dependant on the regional level. Decentralisation gives more flexibility to regional authorities, but this means that differences exist between regions. In order to create a connection between regions, a Strategy for Employment Activation has a provision setting up a best-practice programme, which is a means for transferring good practices between regions.

Employment support in the open labour market

Royal Decree No. 870/2007 provides a framework for supported employment as well as a general financial budget from the state to autonomous communities to support this employment scheme. The definition of supported employment in legislation emphasizes the employment in the open labour market.

Regional governments are tasked to put in place measures facilitating the professional integration of persons with difficulties in entering the labour force, in line with the principles of supported employment. Such measures are different across regions.

For example, the Bureau of Employment in Diversity (Subdirecció General d'Ocupació en la Diversitat) of Catalonia is a responsible agency for the allocation of “subsidies for the realization of comprehensive services for guidance, assistance and support for the inclusion of people with disabilities and/or mental illness” (Subvencions per a la realització dels serveis integrals d'orientació,

acompanyament i suport a la inserció de les persones amb discapacitat i/o malaltia mental, SIOAS). This subsidy can last for 1 year for each participant. In addition, the Barcelona Provincial Council supports the Network of Specialised Employment Offices (Oficines Tècniques Laborals, OTL), which is a network of 16 local employment offices specialised in the employment (re-)integration of persons with mental health conditions. The offices handle the job placement task. In 2014, 393 people were put in employment through the network, with approximately half of them being placed in the open labour market jobs.

When working with persons with mental health problems, some specialised employment intermediation entities follow the IPS methodology to supported employment. Catalonia, for instance, participated in the IPS programme led by the Dartmouth University in the US.

Protections and privileges:

- Greater flexibility and possibility to hire employers on temporary contracts and training contracts. In the time of economic crisis this gives more flexibility to employers. The maximum age for applying a training contract has been extended from 21 to 25 up to 31 years. Employers get a 100% deduction in their employer contributions for these types of contracts. The cost of training workers with these contracts is assumed by the state for companies with less than 50 workers.
- Under the Law 13/1982, employer is obliged to adopt appropriate measures, according to the needs of each specific situation in order to enable disabled people to have access to employment, to do a job, to advance in a profession and to undergo training, unless such measures would entail an excessive burden for the employer;
- The Law 51/2003 imposes a duty to provide reasonable accommodation.

Employment support through Social enterprises or social cooperatives

Under the Law 44/2007, cooperatives can take up the tasks of job placement intermediaries. Companies created by patients' initiatives are, however, limited in scope and difficult to operate in Spain. This is something to be developed.

Employment support through sheltered work

Sheltered work in Spain is provided through Special Employment Centres, where the majority of workforce is made up by persons with disabilities. These centres are regulated by the Royal Decree 2273/1985 and the Royal Decree 469/2006. Some sheltered centres are subcontracted by non-sheltered businesses, who can fulfil their quota requirements by outsourcing from sheltered workshops. Such centres are known as the “work enclaves” and are intended to encourage the transition to the open labour market. These enclaves are regulated by the Royal Decree 290/2004.

Incentives for persons with NCDs to participate in activation programmes

There are no additional benefits paid for participation in vocational rehabilitation and training programmes. However, there is flexibility in combining benefits with work. In Spain, a distinction is made between temporary and permanent disability.

A temporary disability benefit is a daily allowance to cover the loss of income caused by a worker’s illness, an accident or an occupational disease. The benefit can be paid for a period of 365 days (extendable by another 180 days) in a case of a non-work related illness or accident, and for a period of 6 months (renewable for another 6 months) in a case of an occupational disease.

Permanent disability benefits and rules on their compatibility with employment vary depending on the degree of disability:

- Partial permanent disability benefit is paid when the worker's normal performance is impaired by 33% or more, but she/he can carry out the basic tasks of their normal job. This benefit is a lump-sum compensation made in a single payment in an amount that equals to 24 months of the base pension used to calculate the subsidy for the temporary disability from which the permanent disability is derived. It is compatible with any type of employment activity and income level.
- Total permanent disability benefit is paid when the worker cannot carry out basic tasks of the normal profession, but can do another job. The benefit consists of a lifetime monthly pension that, under exceptional circumstances, may be substituted for a lump-sum payment, when the beneficiary is under the age of 60. The benefit is compatible with the salary the worker may receive in the same or another company under a condition that the duties are not the same as those that led to the total permanent disability.

- Absolute permanent disability benefit is paid when a worker is completely disqualified from any profession. The benefit amounts to 100% of the base rate and is compatible with profit or non-profit activities that are in line with the individual's degree of disability and that do not represent a change in the ability to work for the purpose of reviews.
- Severe disability benefit is paid when a worker is completely disqualified from any profession and also requires assistance from another person to carry out daily basic activities. The benefit amount to 100% of the base rate and is compatible with profit or non-profit activities that are in line with the individual's degree of disability and that do not represent a change in the ability to work for the purpose of reviews.

Financial incentives for employers to recruit/retain persons with NCDs

The types of financial incentives and subsidies paid to employers with an aim of promoting the employment of persons in disadvantaged situation vary across autonomous regions.

In general, employers can receive different financial benefits depending on the type of a contract offered to a person with a disability.

Permanent contract:

- A benefit for covering the cost of social security costs per worker ranging from EUR 4,500 to EUR 6,300 per year for full-time contracts, depending on the severity of disability, age and sex. For part-time contracts the amount is based on agreed days of work;
- A subsidy of EUR 3,907 for hiring a person with a disability on full-time basis; For part-time contracts the amount is proportional to agreed days of work;
- A deduction of EUR 6,000 euros from the full amount of the corporate tax per person, under a condition that the recruitment of a person with a disability leads to an increase of the average number of persons with disabilities in the labour force.
- A grant for job adaptation up to EUR 901.52.

Temporary contracts (from 12 months to 3 years):

- A benefit for covering the cost of social security costs per worker ranging from EUR 3,500 to EUR 4,700 per year for full-time contracts, depending on the severity of disability, age and sex. For part-time contracts the amount proportional to the agreed working hours;

- A grant for job adaptation up to EUR 901.52.

Training contract (from 6 months to 2 years):

- A 50% benefit for covering the cost of social security costs for graduates under a condition that 6 years have passed since the completion of their studies.

Apart from these financial benefits, under the Law 30/2007, preference in granting public contracts is given to companies that comply with the legal quota of 2% of people with disabilities in their workforce.

Non-financial incentives for employers to recruit/retain persons with NCDs

An example of the non- financial measures encouraging employers to recruit persons at risks of social exclusion can be seen in the activities of the Incorpora programme (www.incorpora.org), supported by La Caixa Foundation. The programme offers companies the opportunity to acquire a socially active attitude through the promotion of corporate social responsibility. Incorpora works in a close partnership with businesses and provides them with business advice and full support the recruitment and integration process. The emphasis is made on the advantages of hiring persons at risk of exclusion in terms of an improved company image, reduced absenteeism, retaining of talents, and a positive societal impact.

Duties of persons with NCDs

Disability benefits are not conditional on participation in employment activation activities.

Duties of employers

The Law on Social Integration of Persons with Disabilities obliges companies with more than 50 workers to meet the 2% quota on employing people with disabilities.

General and specialised employment services for persons with NCDs

Job placement services to persons at the risk of exclusion from the labour market may be provided by non-profit organisations supporting these groups of people. For example, Parc Sanitari Sant Joan de Deu, headquartered in Sant Boi de Llobregat, provides job placement services for people of working age with a mental health condition who are motivated to join the labour market but face difficulties in the integration process. Job placement services assist job-seekers in preparing job applications, looking for vacancies, providing guidance and creating a job search strategy. Job-seekers receive individualised support but can also participate in support groups, where they can share experiences with other service users.

Another example is the Adecco Foundation, a non-profit arm of the employment agency Adecco that is focused on people who for various reasons face difficulties in finding employment. For all users, the foundation provides access to a job vacancy database, allows registering CVs, and provides assistance in vocational training. In addition to this, it participates in research and development projects aimed at creating assistive technologies facilitating the employment of persons with disabilities. The foundation has been created as result of social responsibility assumed by Adecco.

Job placement services targeted to specific needs of persons with specific health conditions may be rendered through organisations of such patients. The Spanish Association Against Cancer (AECC) has a job placement programme for persons with cancer who require employment support and are registered as jobseekers at the Employment Service of their Autonomous Community. The services are focused towards guidance, training and mediation through the development of personal aspects for the occupation, through the promotion and enhancement of individual skills, training, information and advice. In addition, awareness of entrepreneurship and society as a whole is intended to help facilitate the employment of those affected.

Various services offered to persons at the risk of exclusion from the labour market include:

- Pre-labour services (servicio prelaboral) are social care services to help people with severe mental conditions who are unable to directly access and integrate into work. For this reason they require a vocational rehabilitation process that prepares them for obtaining a job. It involves basic skills necessary for work as well as training for families. Pre-labour services are mainly offered by NGOs.
- Targeted legal, psychological, training advice and guidance – e.g. the Spanish Group of Cancer Patients (Grupo Español de Pacientes con Cáncer, GEPAC) offers its users services

such as legal advice on matters relating to access to employment, education and training; psychological counselling; help in finding jobs and getting interviews; training and empowerment for socio-professional reintegration.

- Occupational rehabilitation services – e.g. Fundación Manantial's Centres for Occupational Rehabilitation develop occupational rehabilitation programmes aimed at helping persons with mental issues to acquire or regain skills needed for work integration.

4.5. "Post-Communist" model

4.5.1. Czech Republic

Main legislative frameworks on chronic diseases, mental health and employment

There is no legislation that regulates specifically the employment of persons with chronic diseases. Eligible persons from this group are covered by the broader legislation on the employment of persons with disabilities. Persons with health problems but without officially recognised disabilities can receive employment support that is available for persons with disabilities in cases when such measures are recommended by bodies assessing their functionality or if NGOs specialised in relevant health conditions (e.g. cancer or diabetes associations) can obtain funding for such support.

Main legislative frameworks on disability and employment

- Act no.198/2009 Coll. on equal treatment and legal means of protection against discrimination.
- Part III of the Law on Employment No. 435/2004 Coll.[61] (on employment of persons with disabilities)
- National Plan supporting equal opportunities for people with disabilities 2015 -2020 (non-binding).
- Act no.518/2004 Sb. Directive on vocational rehabilitation for people with disabilities.
- Act No. 329/2011 Coll. on Providing Benefits for People with Disabilities
- Employment Strategy 2013-2020;
- Ratified the UNCRPD on 28 September 2009.

[61] http://business.center.cz/business/pravo/zakony/zamestnanost/cast3.aspx

Policy provisions on mainstream and specialist employment programmes

Regional Labour offices are responsible for employment services such as counselling, job seeking, job matching, etc. Persons with health issues and disabilities can be referred to vocational rehabilitation, training or retraining programmes by Labour Offices, based on the needs of persons. Such programmes are normally contracted from NGOs and other service providers.

Policy provisions on access to employment support

Vocational rehabilitation is accessible for persons with functional limitations. The functional assessment of persons with disabilities or health problems is carried out by prevocational centres where a common assessment methodology is used (ICF). The assessment is focused on the working potential and individual limitations. There are 13 prevocational centres across the country. Persons can be referred to prevocational centres by general practitioners, other physicians or Labour offices. Pre-vocational centres provide the results of assessment to labour offices, who in turn can provide relevant employment services.

Policy provisions promoting stakeholder cooperation and integration of services

At policy level there is recognition of the importance of inter-agency cooperation, but in practice the cooperation is not well-established. Labour offices are “overwhelmed” and therefore are “passive” at seeking cooperation with local social departments, patients’ organisations, NGOs and companies. A recent policy reform resulted in the partial transfer of duties, including a responsibility for the payments of disability allowances, to Labour offices. However, the responsibility to work with people remained with social care departments of municipalities and there are no mechanisms for sharing data and information.

While labour offices have a range of services supporting the work integration of persons with reduced work capacity, information about these services is often not available for users. Under the National Plan on equal opportunities for persons with disabilities, Measure 13.12 specifically indicated that persons with disabilities should be better informed about the possibility of vocational rehabilitation, counselling and other instruments offered by the Labour office. The Employment Policy strategy of the Czech Republic also highlights the importance of developing Information and

Guidance Centres at Labour Offices of the Czech Republic. Individual service providers also hold awareness raising campaigns.

Policy provisions promoting persons-centred approach and individualised service provision

Labour offices can provide individual action plans for all job seekers to improve their employment chances. Individual action plans are based on personal skills, qualifications and capabilities, and involve a schedule and description of measures to be taken in the process of job search. In addition to this, persons with disabilities are entitled to have individual plans for vocational rehabilitation compiled for them by Labour offices (in cooperation with persons with disabilities). However, personal support provided by Labour Offices is deemed insufficient in practice. According to an opinion of one user organisation in the Czech Republic, vocational rehabilitation programmes sometimes "target different needs" and staff at Labour Offices do not have a "personalised approach" and "passively register the unemployed and pay disability benefits."

Policy provisions on localised and accessible employment service provision

The central body overseeing the (re-)integration of disadvantaged groups in the labour market is the Ministry of Labour and Social Affairs. The Ministry manages and provides methodological guidance for Labour offices, which are spread across regions. Individuals can access Labour offices based on the address of their permanent residence. Labour offices work together with prevocational centres in respective regions. In total there are 13 prevocational centres in the Republic.

In addition, for persons with disabilities, there are programmes of supported employment, sheltered workshops, social enterprises that are scattered unevenly across the country: while in some regions these programmes are poorly developed, in others they work very well.

Prominent actors in the field of professional (re-)integration services are government funded Labour offices that work in cooperation with prevocational centres. Labour offices work provide vocational rehabilitation and retraining programmes for persons with disabilities, which can be outsourced.

European and state funding plays an important role in supporting NGOs in their projects on supported employment and employment integration. Individual NGOs and patients associations can develop employment programmes tailored to the needs of their target groups.

Employment support in the open labour market

The concept of supported employment is not explicitly mentioned in Czech legislation but its main principles are followed in designing other tools used to support persons with disabilities and ill-health in employment. The main tool used in the Czech Republic for this purpose is vocational rehabilitation (Pracovní rehabilitace). The tool was introduced in Part III of the Law on Employment No. 435/2004 Coll.[62] It is defined as a continuous activity aimed at obtaining and maintaining suitable employment for people with disabilities. Vocational rehabilitation envisages employment in the labour market and involves measures tailored to individual needs and counselling. The Labour offices provide funding for vocational rehabilitation and works in cooperation with employers, educational institutions, NGOs, doctors and health care services. Vocational rehabilitation is accessible for persons with disabilities but also for persons with health conditions causing a reduced working capacity in cases when this intervention is recommended by an attending doctor or by social security authorities. Following are tools that could be used as part of vocational rehabilitation:

- Retraining (rekvalifikace) - Labour offices can recommend and facilitate retraining support jobseekers. Funding is provided for costs associated with retaining as well as for retraining support (equivalent to 60% of the average income received at the most recent work but not exceeding 65% of the average wage in the Czech Republic) to persons undergoing retraining courses for the same period as unemployment support. Retraining must be approved by the Labour Office and agreed with the jobseeker.
- Counselling programmes (poradenské programy) - are organized as group activities aimed at promoting job seekers' self-confidence and motivation. These programmes try to develop and deepen communication, computer and presentation skills as well as to provide information about job search and job management techniques.

Despite the fact that national legislation does not have specific provisions on supported employment programmes, such programmes do exist. In 2000, the Czech Union for Supported Employment was established with an aim to promote, facilitate the development of supported employment. The

[62] http://business.center.cz/business/pravo/zakony/zamestnanost/cast3.aspx

union has numerous members that provide supported employment services to persons with different types of disabilities, mental health problems and chronic diseases.

Employment support through Social enterprises or social cooperatives

Social cooperatives are legally recognised by the Czech law on commercial corporations No. 90/2012 Coll. as entities that pursue beneficial activities to promote social cohesion through work and social integration of disadvantaged people in society, prioritizing the satisfaction of local needs and utilization of local resources particularly in the area of job creation, social services and health care, education, housing and sustainable development. The Ministry of Labour and Social Affairs provides grants for social enterprises specialised in work integration of disadvantaged groups.

Employment support through sheltered work

A sheltered work position (chráněné pracovní místo) is a position created by an employer for an individual with a disability based upon a written agreement with the Labour office. A sheltered workshop is a work unit in which a minimum of 60% of the employees are persons with disabilities. Financial support for operating a sheltered working place or sheltered workshops can be provided through a partial reimbursement of operating costs and labour costs. In general, public employment offices try to place jobseekers in the open labour market through vocational rehabilitation, with sheltered employment being an option of last resort for persons with severe disabilities. Negligent implementation of vocation rehabilitation programmes, however, leads to an increasing number of persons with disabilities in sheltered workshops, according to a user interviewed as part of the study.

Incentives for persons with NCDs to participate in activation programmes

Persons undergoing retaining (approved by a Labour office) can receive retraining support (equivalent to 60% of the average income received at the most recent work but not exceeding 65% of the average wage in the Czech Republic) for the same period as unemployment support.

Persons with disabilities can be entitled to:

- Full disability pensions – paid to insured persons who become “fully disabled” as a result of an injury at work or an occupational disease;
- Partial disability pensions - paid to insured persons with a disability caused by an injury at work or an occupational disease.

Partial disability benefits may be compatible with work in certain cases:

- If monthly income earned does not exceed 66% of the personal assessment base on which the pension was calculated, the partial disability pension is paid in its full amount.
- If monthly income is between 66% and 80% of the assessment base, the partial disability pension amounts to 50% of the calculation base.
- If monthly income exceeds 80%, the partial disability pension is not paid.

Partial disability pension is paid in its full amount, if the total earned income in a calendar year does not exceed the sum of the minimum subsistence level multiplied by twelve (effective on 1st January of the relevant calendar year in which the average monthly income of the person concerned is assessed). Partial disability pension is also paid regardless of earned income, if the person was awarded pension due to disability considerably aggravating her/his general living conditions. This type of pension is awarded even if the beneficiary works abroad. In other cases when the person concerned works abroad, the partial disability pension is not paid.

Act No 329/2011 Coll. on Providing Benefits for People with Disabilities and Amendments of Related Acts, as amended, regulates financial benefits (allowance for mobility, grant for special aid) and the certificate of a person with a disability. Through them the State helps to reduce social consequences of disability and support social inclusion.

The country’s Employment strategy 2020 promotes measures aimed to reduce the dependence of certain groups on social incomes.

Financial incentives for employers to recruit/retain persons with NCDs

By law, employers with a workforce where more than 50% are persons with disabilities are entitled to a financial contribution. Business entities that create jobs for persons with disabilities can receive a financial contribution of up to eight times the average wage by their respective labour office. According to Act No. 435/2004 Coll. on Employment, Part Three, Section 79, Labour Offices are also

responsible for providing information and advice on issues related to employing people with disabilities, as well as funding for adaptations to workplaces in the open labour. In addition, employers receive a discount on income tax and can be fully reimbursed for costs of training for employees with disabilities.

Non-financial incentives for employers to recruit/retain persons with NCDs

This type of incentivising is not emphasized in policy initiatives. However, there are initiative coming from non-profit organisations, such as Rytmus - Od klienta k občanovi o.p.s., which works on explaining employers the benefits of working with persons with disabilities. Among other things the NGO emphasizes the more effective use of qualified employees, strengthening of companies' social responsibility, promotion of good practices and improved corporate culture[63]. Initiatives such as this one are not too common and are rather promoted by individual organisations and not by the government.

Duties of persons with NCDs

Persons with a disability are not obliged but rather have a right to seek employment. Their disability benefits are not dependent on their participation in vocational rehabilitation activities.

Duties of employers

Under the Act No. 435/2004 Coll., employers with workforce of more than 25 people are required to employ individuals with disabilities to the proportion of 4% of the total number of employees. This obligation can be met by:

- Actually employing persons with disabilities;
- Buying products or services from employers with a workforce consisting for more than 50% of persons with disabilities;
- Paying a penalty to the state;

[63] http://www.czechmarketplace.cz/en/11515.discussion-on-best-practices-for-employers-of-people-with-disabilities

- Combing all the above-mentioned methods.

Under the National Plan 2015-2020 on equal opportunities for persons with disabilities, public administration authorities and their organizational units should strive for direct employment of persons with disabilities, increase the number of employees with disabilities and submit data on the development of the employment of persons with disabilities in the individual ministries.

General and specialised employment services for persons with NCDs

CEREBRUM, an Association of persons with brain injury and their families, provides services such as cognitive training, requalification programmes, occupational therapy, functional and vocational assessment, self-help groups, cooperation with employers, social advice and support, practical display of various professions

4.5.2. Poland

Main legislative frameworks on chronic diseases, mental health and employment

There is no legislation that regulates specifically the employment of persons with chronic diseases. Eligible persons from this group are covered by the broader legislation on the employment of persons with disabilities. The existing legislation is only applicable for people with an officially recognised disability.

Main legislative frameworks on disability and employment

- Charter of Persons with Disabilities of 1 August 1997;
- Act of 27 August 1997 on Vocational and Social Rehabilitation and Employment of Persons with Disabilities.
- Ratified the UNCRPD on 25 September 2012.

Policy provisions on mainstream and specialist employment programmes

Public employment services are the main bodies in Poland that provide a labour market programmes aimed at job-seekers and unemployed persons. According to the Act of 27 August 1997 on Vocational and Social Rehabilitation and Employment of Disabled Persons, persons with disabilities, registered in labour offices as unemployed or job-seekers, have the right to use public employment services. However, some labour market programmes are only accessible by people registered as unemployed and people with disabilities who are entitled to incapacity pensions cannot register as unemployed. Persons with disabilities are also entitled to support specific to their situation. NGOs (e.g. Foundation Activation, Polish Association of Disabled People) assist the employment services in targeted job search and job matching.

Employment services for jobseekers (such as career counselling, support in preparing CVs, job search) can be provided by projects, but these projects operate in short term and lack continuity.

Article 11 of the 1997 Act on Rehabilitation gives a right to persons with disabilities registered at labour offices as job-seekers to access some services and instruments (such as training, internship, a programme of intervention which provides subsidised, postgraduate courses) on same basis as other unemployed persons. These expenditures are, however, financed by the State Fund for Rehabilitation of Disabled Persons (PFRON) and not by the Labour Fund, which is the case in standard circumstances.

Policy provisions on access to employment support

The country uses a rather medical approach towards disability that is based on the judgement of a medical expert. Applicability of employment policies for persons with disabilities are determined by assessments made by Disability Evaluation Boards. A legal document that confirms disability, a “certificate of disability,” is issued by the poviat[64] disability assessment team on the basis of a medical expert's opinion. The expert's opinion is crucial because the same type of disability may be qualified at different levels in different parts of the country. According to the 1997 Act on Vocational and Social Rehabilitation and Employment of Persons with Disabilities, there are three levels of disability: severe, moderate and minor. Severe disability means a very restricted functional capacity, a full incapacity to work or a capability to work only in sheltered work. Moderate disability means

[64] Poviat is a second-level unit of local government and administration in Poland. It is part of a voivodeship, the highest-level administrative subdivision of Poland.

that a person can work if given support and in a work environment adjusted to her/his needs. A minor disability means a person can work without support. In terms of access to support in employment (re-)integration, a disability certificate is a prerequisite.

According to Polish laws, such as the Act on Pensions from the Social Insurance Fund or the Act on Social Pension, terms such as "inability to work" and "inability to live independently/need for long term care" are used. This creates a nomenclature issue as these terms may be misleading: employers often think that people classified in these categories cannot work at all.

Policy provisions promoting stakeholder cooperation and integration of services

Inter-agency cooperation and integration of employment support services is not emphasised at policy level. The system of labour market activation for persons with disabilities is in general fragmented and cooperation between sectors is limited.

In order to increase users' and employers' involvement, NGOs can obtain funding from PFRON for services such as information centres, preparation of workshops and conferences. However, financial resources given to NGOs are limited.

Social campaigns are carried out by NGOs and government bodies to promote the employment of people with disabilities (e.g. "Wroclaw without barriers" by the Wroclaw commune). NGOs and organisations of persons with disabilities also hold information meetings with employers to encourage them to hire persons with impairments.

Legal advice is also often offered by service providers to help job seekers in administrative issues (e.g. information about disability certificates).

Policy provisions promoting persons-centred approach and individualised service provision

There is a legislative base for ensuring reasonable adaptation at the workplace and to address the individual needs of a person at work.

Policy provisions on localised and accessible employment service provision

Administrative reforms in the field of professional/social rehabilitation and employment of persons with disabilities have led to the transfer of the majority of relevant tasks to local governments. The voivodship-level local governments are responsible for carrying out tasks related to the (re-)integration of persons with disabilities in employment. The main three types of tasks include:

- Co-financing construction works: building infrastructure for rehabilitation of the disabled persons (including medical as well as professional and social rehabilitation);
- Co-financing professional activities within VRFs for professional activation of persons with disabilities;
- Cooperation with NGOs, co-financing of projects aimed at professional activation.

Despite the reforms, the system of professional activation of persons with disabilities in Poland remains to some extent centralised because it is organised around PFRON. Money from this state fund is transmitted to the local governments. According to some decentralization proponents, the tasks of PFRON should be further delegated to voivodeships. The current situation, where both national and local authorities are responsible for activation initiatives, creates a complicated system with entities with overlapping and duplicating tasks.

Under the Polish laws, both the national government and local governments finance and oversee actions aimed at supporting persons with disabilities in employment. However, they do not do it directly; services are provided by non-governmental organisations, often through projects. EU funding plays an important role in financing projects aimed at supporting persons with disabilities in work integration. Services provided through project do not have a commercial nature. However, when funding stops, it becomes unsustainable to continue service provision. Therefore, NGO Foundation Activation (www.aktywizacja.org.pl), for example, tries to provide commercial training courses and career counselling services, but finds it difficult to sustain.

Various counselling, training courses are provided through the frameworks of Occupational Therapy Workshops, Vocational Rehabilitation Facilities, NGOs and EU-funded projects. These entities may provide services internally or outsource them.

Employment support in the open labour market

Supported employment is not integrated in PES, but is rather provided on project basis. The PFRON provides funding for services such as job coaching.

Privileges in the open labour market:

Within the open labour market, persons with disabilities receive certain privileges such as additional 10 days of leave or reduced working hours. For example, the labour law gives a possibility to work 7 hours per day if health conditions do not allow persons with disabilities to work 8 hours per day. These provisions may, however, discourage employers from hiring persons with disabilities. In some cases, employers simply disregard these privileges.

Under the 1997 Act on Rehabilitation, employers that employ persons with disabilities for at least 36 months may receive reimbursement from the PFRON for adapting workplaces to the needs of people with disabilities, adapting or buying devices that help people with disabilities to function at work and recognising the needs of people with disabilities for medical services. In addition, since 2009, employers can be reimbursed for the costs of special software and assistive technologies and equipment. The reimbursement cannot exceed 20 times the average remuneration. When employing persons with disabilities who have been unemployed or seeking jobs for at least 36 months, reimbursement cannot exceed 15 times the average remuneration related to equipping workplaces. While legislation has provisions for reimbursement of workplace adaptation, in practice this often does not take place. The PFRON does not cover the full costs of adaptation and adaptation is usually very expensive.

Although PFRON and local authorities provide financial support and subsidies for employers to facilitate the employment of persons with disabilities, some employers find it burdensome to apply for funding. The system is quite complicated and bureaucratic, which requires companies to have experienced accountants and HR staff to handle the administrative procedures related to application for support and financing. Therefore, small companies with scarce financial and human resources lack capacities to engage in such programmes.

Employment support through Social enterprises or social cooperatives

In Poland, social cooperatives are established with at least five members who are unemployed or have various disabilities with an aim to promote the social and professional reintegration of their

members into the labour market. As of 2015, there were close to 1310 registered social co-operatives, although not all of them remain active[65].

Other forms of social enterprise are Occupational Therapy Workshops and Vocational Rehabilitation Facilities.

Occupational Therapy Workshops (warsztaty terapii zajęciowej) offer the first stage of the professional reintegration. Persons with disabilities are offered social rehabilitation and self-efficacy building in combination with activities aimed at professional activation. Personal predispositions are checked as well as manual skills, etc. When the individual reaches a certain level of social rehabilitation and there is a general idea of professional activity appropriate to this particular person, then she/he moves on to the next stage - Vocational Rehabilitation Facilities.

Vocational Rehabilitation Facilities (VRFs, zakład rehabilitacji zawodowej) are autonomous units of local authorities, NGOs or social co-operatives that offer employment for persons with disabilities and in addition provide social, medical and professional rehabilitation for them. VRFs run by districts, municipalities or NGOs and are financed by PFRON and/or local governments. In Malopolska voivodeship, for example, as of March 2016, there were eight VRFs employing a total of 278 persons, including seven run by NGOs and one run by a district. VRFs are engaged in economic activities (e.g. production, services) but, unlike sheltered workshops, they cannot keep the generated profit: all proceeds have to go back to PFRON or local authorities. Therefore, VRFs are not necessarily interested in being economically sustainable and come at a significant cost to the state.

For employees, VRFs offer individual development and activation programmes tailored to individual needs. There are also special commissions evaluating the development each person. When the VRF employee reaches a certain level of professional development, she/he can move on to the open labour market and her/his position is filled by another person with a disability. If no development is observed, the employee is transferred to sheltered work.

In practice, the problem with VRFs is that programme users often remain in the facilities without reaching the open labour market. One reason for this might be the inadequacy and ineffectiveness of individual development and activation measures. Another reasons is that VRFs run businesses and are not interested in losing valuable and experienced workers. Therefore they chose to keep them,

---

[65] A map of social enterprises and their eco-systems in Europe: Poland

which in turn blocks an opportunity for other candidates to take up a position at a Vocational Rehabilitation Facilities. Thus, instead of being transitory programmes intended to prepare candidates for an open market job, VRFs become relatively permanent solutions for persons with disabilities.

An example of a VRF is the U Pana Cogito guesthouse in Krakow.

In addition to the above-mentioned forms of social entities, there are also social firms. For example, LABORATORIUM COGITO SP. Z OO is a limited liability company established in 2005 by the Association for the Development of Psychiatry and Community Care. It is a special type of commercial companies that employs people with disabilities (more than 85% of employees have mental health issues). The company provides different types of services: hospitality, catering and bike rental.

Employment support through sheltered work

In Poland, sheltered work is a pathway for persons who were not able to transition into the open labour market after Occupational Therapy Workshops and Vocational Rehabilitation Facilities. Usually sheltered workshops employ persons with severe disabilities.

Incentives for persons with NCDs to participate in activation programmes

Persons with disabilities do not receive any additional benefits for participation in employment activation programmes.

Receiving disability pension can be combined with paid work but only to a certain threshold of the amount of income.

Financial incentives for employers to recruit/retain persons with NCDs

Employers with a workforce consisting of 50% of persons with disabilities are entitled to benefits such as tax exemption and exemption from stamp duty. They can also have a partial reimbursement for training of employees with disabilities.

Wage subsidies

As of 1 April 2014, a monthly wage subsidy is the same for all employers, irrespective of their status, and amounts to:

- PLN 1,800 for people with a severe disability;
- PLN 1,125 for people with a moderate degree of disability;
- PLN 450 for people with a minor disability.

The amounts mentioned above are increased by PLN 600 for people with mental disabilities, developmental disorders, epilepsy or visual impairments.

Non-financial incentives for employers to recruit/retain persons with NCDs

Awareness raising and social campaigns are launched by NGOs to promote diversity in employment and to support disadvantaged groups in employment. In addition, some businesses support social organizations working for disabled people (charity actions, money for removing of architectural barriers, purchase the computers for the people with visual impairments, etc.)

Duties of persons with NCDs

Persons with disabilities are not obliged to undergo vocational rehabilitation or to participate in employment activation programmes before being eligible for disability benefits.

Duties of employers

Public and private enterprises with 25 or more full-time employees are required to ensure that 6% of their workforce consists of persons with disabilities. In the sector of education, the quota is 2%. Employers are supposed to pay a penalty if they fail to fulfil the quota.

General and specialised employment services for persons with NCDs

- TELEKARIERA (www.telekariera.pl), which ran from 2011 to 2015 and aimed to support people with respiratory and circulatory system disabilities in employment. The project included three stages of support: 1) Psychological, psychosocial and professional counselling (individual sessions with a career counsellor, psychologist, and lawyer); 2) Training – social and professional competences (Self-management, professional goals, entrepreneurial skills, creative thinking, methods of time management, Basic computer skills, MS Office, other professional courses); 3) Three-month work placement (internships tailored to individual needs).
- Training and supported employment services for disabled people with mental health issues are provided by the Association for the Development of Community Psychiatry and Care. PFRON can provide funding to services of job coaches and personal assistants to help persons with disabilities overcome psychological and physical barriers at work.
- "Against Social Exclusion of people with mental disorders" project aimed at overcoming barriers in labour market for persons with mental health issues. It was co-funded by Norway Grants and the ESF. The project was run by the Open the Door Association (Stowarzyszenie Otwórzcie Drzwi) and targeted both persons with mental health problems and professionals working with them.

4.6. Comparative analysis

Main legislative frameworks on chronic diseases, mental health and employment

In all of the countries reviewed in this study, there are no legal frameworks specifically covering the employment integration of persons with chronic diseases. In most cases persons with NCDs are

considered as part of a group of persons with disabilities and reduced work capacity. Therefore, they are largely covered by legislation for persons with disabilities.

In the case of mental health issues, more specific frameworks are available. For instance, Norway has in place a National Strategic plan on Work and Mental Health, and in the UK, a national strategy, "Five Year Forward View for Mental Health," for the National Health Service in England has been published.

Main legislative frameworks on disability and employment

Legislative frameworks on disability in all countries provide a solid foundation for fighting against discrimination in employment and employment services. All countries have ratified the UNCRPD and at least at policy level recognise the rights of persons with disabilities to equal work opportunities. Depending on the definition of disability in different countries, persons with chronic health conditions can be recognised as disabled and be protected from unfair treatment or benefit from additional supports.

Policy provisions on mainstream and specialist employment programmes

In all countries PES aim to provide services to persons with reduced work capacity and have units or personnel that can refer jobseekers with specific needs to specialised services.

Policy provisions on access to employment support

All countries provide support to persons with disabilities or persons from vulnerable social groups in finding, getting and staying in employment. However, there may be cases when persons with NCDs may be denied access to such supports and rehabilitation measures. In all countries from the Mediterranean welfare model, disability is a prerequisite for additional support in job seeking. In Greece, for example, persons with NCDs with a disability level below 50% cannot access employment rehabilitation programmes, regardless if they still have support needs. A similar situation exists in Austria, but the assessment of disability varies in both countries. In some cases, as in Poland, people have no chance to work at all if they are found "incapable of doing any gainful

work". In Spain, persons with chronic diseases, however, still have a possibility to access assistance in job adaptation. In the UK and Norway, persons with health conditions are included in provisions for employment support. For example, in the UK, persons with cancer would automatically get access to same services that are available for persons with disabilities.

Policy provisions promoting stakeholder cooperation and integration of services

Differences exist in the degree of cooperation between healthcare and employment bodies in defining rehabilitation plans for persons with NCDs. At the stage of assessment, some countries still rely on a medical approach, without taking into account other factors (IT, SI, PL, EL).

In terms of the cooperation between companies and healthcare professionals, countries like UK and Norway have return-to-work or long-term absence management mechanisms that enforces the link between these stakeholders. In this sense, the IA-Agreement in Norway is an example of the government's attempt to ensure a greater involvement of employers. The Corporate Integration Management System (BEM) in Germany is another example.

Policy provisions promoting persons-centred approach and individualised service provision

The understanding of the importance of a person-centred approach and individualised services is seemingly shared by all countries, at least on paper. However, when it comes to the actual implementation of such services, a lot depends on the personnel of the employment services handling the case. Like in the case of Czech Republic, a lack of sufficient funding overwhelms labour offices, thus creating a risk that the needs of jobseekers are not adequately assessed.

Policy provisions on localised and accessible employment service provision

Mediterranean countries like Italy and Spain have differences between regions in terms of supports and services available. While difference can be regarded as a possibility to provide more diverse and locally suited support mechanisms, it may also create unequal services for people depending on where they live. In the UK and Norway, employment services that operate through local branches, are in this sense more uniform.

Employment support in the open labour market

Policies in all countries are targeted to integration into the open labour market. However, the effectiveness of such measures and the quality of their implementation can vary.

Supported employment schemes are embedded in national policies and strategies in some countries (DE, AT, SI, ES) and takes different form while keeping to the same principles in others. In Poland, for example, supported employment services are not mainstreamed but are rather available through individual projects (often funded by the EU).

Employment support through Social enterprises or social cooperatives

Social enterprises take different forms across countries. One of the most varied and business-focused forms of social economy presented in this study is the one of the UK. The market approach can be seen in the business approach and terminology that is used with regard to social enterprises as they draw resources from social 'investments," rather than government "subsidies." In some cases, social enterprises are so dependent on government support or not interested in economic sustainability, that their 'commercial' activities can be questioned (e.g. some VRFs in Poland).

Employment support through sheltered work

In most countries, sheltered workshops are considered as last resort for persons who are not able to be employed in the open labour market. They are often targeted at persons with (severe) mental disabilities and some people with mental health issues but are not entirely relevant for persons with other chronic diseases.

Incentives for persons with NCDs to participate in activation programmes

There are basically to ways in which persons with NCDs can be incentivised:

- Benefits that are conditional on participating in work-related activities (e.g. AAP, Qualification benefit, and Support when participating in measures in Norway, ESA for certain recipients in the UK)
- Possibility to keep benefits while working. Most countries give this possibility depending on the degree of disability (e.g. DE, CZ, and ES).

Financial incentives for employers to recruit/retain persons with NCDs

Most countries provide wage subsidies, nearly all temporary, with exception of the UK.

Non-financial incentives for employers to recruit/retain persons with NCDs

Non-financial incentives mainly take a form of corporate social responsibility commitments. Larger companies that have occupational health therapists also have a possibility to provide return-to-work programmes and manage long-term sick leaves with an aim to have a positive impact on their workforce. In the UK and Norway, where there are no quota obligations or substantial financial incentives for employers, emphasis on non-financial incentives is important.

Duties of persons with NCDs

There are mainly three groups of countries with regard to the responsibility of persons with disabilities (including NCDs) to participate in activation measures:

- Countries that have a rehabilitation-before-benefits rule (NO, UK);
- Countries that have rehabilitation-before-benefits provisions in place but are not (adequately) implemented (DE, AT);
- Countries that do not have the rehabilitation-before-benefits rule (All Mediterranean and Post-Communist model countries reviewed in this report).

Duties of employers

Most countries have quota systems with exception of the UK and Norway.

General and specialised employment services for persons with NCDs

Among the categories of chronic health conditions considered in this study, mental health condition is the one that most frequently has specialised services. Employment services for mental health patients are specific in a way that they require more psychological support and follow up from the side of coaches. For the rest of the categories of NCDs, the services are less specialised and persons with those condition use general services available for persons with disabilities. Exceptions are made when patients' associations, specialised in specific diseases, provide services to their target users (e.g. cancer associations, associations of persons with respiratory system problems). Such services usually focus on provision of information, support in coping strategies, etc.)

V. Conclusions and recommendations

The mapping of policies, systems and services facilitating the inclusion of persons with NCDs at European and national level has revealed that in most cases people from this group are considered as part of a group of persons with disabilities, including persons with reduced work capacity due to illnesses. Persons with specific chronic health conditions considered in this study can mainly receive support in employment if their condition can be recognised as a disability in their countries (reaching a certain eligible degree of disability) or have a negative impact on their work ability.

The study shows that there is a general consistence between European and national legal frameworks with regard to the activation of persons with disabilities and disadvantaged groups. Countries considered in this report do put in place provisions to support activation and greater labour market participation, but they do it in different ways.

In terms of policies, all countries have legislative frameworks against discrimination and provide some support to persons with disabilities and illnesses. Policy-level strategies targeted at activating persons with chronic diseases, are, on the other hand, more limited. They are targeted through strategies for broader groups (persons with disabilities, vulnerable social groups, elderly, etc.). Most policies highlight the significance of availability of mainstreamed, person-centred, integrated and accessible employment services. However, the implementation of policies often does not go in line with the initial commitments, thus hampering the effectiveness of policies and programmes. In addition, the existence of legal initiatives on work activation of persons with chronic conditions does not necessarily coincide with a change in attitudes towards their employment in the society.

In terms of systems, countries differ from each other based on how much emphasis they put on supports, incentives or obligations in order to facilitate the integration of persons with disabilities and reduced work capacity. For instance, as an integration policy-oriented country, the UK provides less categorised support services, no financial incentives to employers in a form of wage subsidies, and requires unemployed persons with reduced work capacity to participate in work-related activities. Norway, a Nordic welfare state, operates in a similar way, but it does provide wage subsidies to employers and provides a wide range of services aimed at empowering workers with health problems. Continental welfare states considered in this study have more categorisation in terms of disability recognition, which makes the access to certain employment supports more difficult. These countries provide financial incentives and use quotas to activate employers but do not impose additional requirements on jobseekers. In Mediterranean welfare states the situation is

fairly similar. Greece, however, due to financial difficulties has very limited supports and activation measures. There, as well as in Post-Communist states considered in this report, funding from the EU plays an important role in providing support.

In terms of systems, the range of specialised services for most categories of chronic conditions is limited. Persons with chronic conditions receive mainstream employment services or services tailored for persons with disabilities or reduced work capacity. Out of all the categories of chronic conditions considered, for mental health issues there are more specialised strategies in place. This may be explained by the markedly different needs of persons with such conditions and the fact that mental health has been high on the international agenda.

Based on the findings of this report, the following recommendations can be made:

More focus on chronic diseases

- The growing prevalence of chronic diseases and their impact on productivity and labour market participation necessitates an increased awareness of the need for extensive policy level strategies for the inclusion of persons with chronic conditions in employment.
- Emphasis should be made on the lack of policy-level strategies specifically targeting the employment activation of persons with NCDs and on the fact that strategies targeting persons with disabilities do not necessarily address the needs of patients with NCDs since the employment needs of these two groups are not the same.
- More research is needed into the effectiveness of existing strategies for (re-)integration of persons with disabilities when these are used for persons with NCDs
- More research is needed into the needs of persons with different chronic diseases in employment support.
- Innovative and needs-based programmes and support measures for professional (re-)integration of persons with NCDs should be put in place.

A more integrated and favourable service provision environment

- Persons with NCDs require integrated employment services because their needs encompass different areas such as healthcare, social and psychological support. Therefore, provisions for integrated employment support should be promoted.
- Better coordination between healthcare and employment should be ensured in order to ensure a better understanding of the relationship between work and chronic diseases.
- Health professionals should be trained to provide work-related advice to persons with NCDs to facilitate a quicker return to work.
- Better cooperation is also needed between employers and healthcare and employment service professionals as employers need to be informed about specific health-related need of workers in terms of work adjustments and overall inclusion in the labour force.

- Persons with NCDs should be able to access adequate and customised employment services in their localities. In addition, measures should be taken to ensure equal level of services availability in different regions.
- Provisions should be put in place to promote the development and availability of public and private employment service provision, ensuring the link between the sectors through cooperation and subsidiarity.
- Systems facilitating employment (re-)integration of persons with NCDs should not consider support programmes, systems of incentives and systems of obligations as separate elements. Instead, these should be seen as part of a comprehensive strategy.
- Emphasis should be made on employment in the open labour market. The move away from unsustainable sheltered work should be encouraged towards sustainable work.
- Labour market policies should be made more flexible with regard to the entry and exit of persons with NCDs and disabilities since the rigidity of overly protective measures may discourage employers from employing persons with health conditions.
- Support in financing workplace adjustments (including removal of physical and non-physical barriers) should be available not only to persons with a recognised disability but also to persons diagnosed with NCDs.
- Support measures such as job coaching, mentoring, counselling should be available for persons at all stages of employment (i.e. finding, getting and staying at a job).
- The economic sustainability of social enterprises should be encouraged through measures creating favourable conditions for competitive and commercial activity as well as through the availability of social investments.
- Sheltered workshops should be transformed in order to target the transition towards the open labour market.

More empowerment for persons with NCDs

- Employees and jobseekers with NCDs should be better informed about their work-related rights and about the availability of support. In most cases, employed persons who acquire chronic conditions often do not need basic labour market integration training but rather need help in understanding their conditions and the ways in which they can cope with the barriers created by their conditions in the work environment. In such cases, support from psychologists or peers with similar conditions may prove more useful.
- Legislative frameworks should ensure the accessibility of existing employment support to persons with NCDs. The eligibility criteria for employment support need to be more flexible and not conditional on disability certification, especially if the assessment is based on the medical approach to disability.
- Policy provisions should focus on the capacity to work and on the tasks that persons with NCDs can perform rather than on the inability to work.
- Specialised support to persons with reduced work capacity, including persons with NCDs, should not be segregated from mainstream employment services. The segregation may lead to the false perception by employers and jobseekers themselves that persons with NCDs and persons with disabilities in general are of lower grade.

- The person-centred approach towards supported employment service provision needs to be further promoted since, as mentioned above, persons with different chronic conditions may have different employment needs. Furthermore, supported employment should be explicitly made available for persons with NCDs.
- Persons with NCDs should be encouraged to first participate in employment activation programmes before considering to become passive recipients of disability benefits.
- Passive benefits payment should be avoided when there are other options as there is evidence that in countries with no permanent or temporary disability benefits, the inactivity rate among people with chronic diseases is lower (OECD, 2007).
- Measures need to be put in place to financially support persons with NCDs during the time when they participate in employment activation programmes.
- Measures allowing persons with NCDs to keep disability or similar benefits while working need to be in place. Persons with NCDs may receive lower salaries or less salary due to a reduced working capacity and therefore, they may still need welfare support. The possibility to combine work and financial assistance is important for avoiding the benefit trap.

More involvement from the part of employers

- Policies and systems should focus on a greater involvement of employers as they are the ones who at the end of the day should provide employment to persons with NCDs. Their cooperation is vital and there is a need for developing strong, innovative and consistent strategies for ensuring their commitment to the inclusion of persons with NCDs.
- The efficiency of financial incentives paid to employers in the form of wage subsidies and tax reductions should be further explored with regard to persons with chronic conditions.
- Efforts to increase the understanding among employers about operational benefits of employing and keeping persons with NCDs should be further promoted with emphasis on advantages for business such as the availability of a larger pool of talents, greater work commitment and loyalty, a possibility to increase a customer base by having a more diverse staff, corporate social responsibility, a greater work-satisfaction by the workforce, etc.
- Further exploration is needed of the applicability and effectiveness of the quota system for the integration of persons with NCDs in work.
- The needs of both employers and (potential) employees should be equally considered for effective job matching, which means that job matching should not be subject to unnecessary impositions and mandatory placements that disregard task-related requirements.
- Services aimed at supporting awareness raising and training for staff and management to better understand the needs of persons with NCDs should be strongly encouraged.
- More services for employers should be offered to better manage illness-related long-term absences and return-to work mechanisms.

Bibliography

American Diabetes Association (2004) “Diagnosis and Classification of Diabetes Mellitus”, *Diabetes Care*, 27(1): 55-60, Available at: http://sites.google.com/site/indicedelboticario/DiagnosisandClassificationofDiabetesMellitus.pdf

Academic Network of European Disability Experts (ANED) (2013) “European comparative data on Europe 2020 & People with disabilities”, Final report prepared by Stefanos Grammenos from Centre for European Social and Economic Policy (CESEP ASBL), Available at: http://www.humanconsultancy.com/_layouts/15/WopiFrame.aspx?sourcedoc={EAFFD3CF-6972-47A4-9BAC-44805B6E0B07}&file=ANED%202013%20Task%206%20-%20comparative%20data%20synthesis%20report%20-%20Europe2020_final.doc&action=default

Berkhout, E., Sattinger, M., Theeuwes, J. and Volkerink, M. (2012)” Into the Gap: Exploring Skills and Mismatches”, Commissioned by Randstad, SEO Economic Research, Amsterdam. Available at: https://www.randstad.gr/editor/uploads/files/Surveys/Into-the-Gap.pdf

Bevan, S., Quadrello, T., McGee, R., Mahdon, M., Vavrovsky, A. and Barham, L. (2009) “Fit For Work? Musculoskeletal Disorders in the European Workforce”, The Work Foundation, London, Available at: http://www.fitforworkeurope.eu/Website-Documents/Fit%20for%20Work%20pan-European%20report.pdf

Bloom, D.E., Cafiero, E.T., Jané-Llopis, E., Abrahams-Gessel, S., Bloom, L.R., Fathima, S., Feigl, A.B., Gaziano, T., Mowafi, M., Pandya, A., Prettner, K., Rosenberg, L., Seligman, B., Stein, A.Z., and Weinstein, C. (2011) “The Global Economic Burden of Noncommunicable Diseases”, World Economic Forum, Geneva http://www3.weforum.org/docs/WEF_Harvard_HE_GlobalEconomicBurdenNonCommunicableDiseases_2011.pdf

Busse, R., Blümel, M., Scheller-Kreinsen, D. and Zentner, A. (2010) “Tackling chronic disease in Europe Strategies, interventions and challenges”, World Health Organization, on behalf of the European Observatory on Health Systems and Policies, Available at: http://www.euro.who.int/__data/assets/pdf_file/0008/96632/E93736.pdf

Campos-Matos I. and Kawachi I. (2015) "Social mobility and health in European countries: Does welfare regime type matter?" *Social Science & Medicine*, 142 (2015): 241–248.

Caritas Europa (2012) "The Future of the Welfare State: A Comparative Study in EU-countries," Lambertus-Verlag, Freiburg im Breisgau, Available at: http://www.caritas.eu/sites/default/files/publication_caritas_europa_-_future_welfare_state_0.pdf

Corral, A. Durán, J. and Isusi, I. (2014) "Employment opportunities for people with chronic disease", IKEI Research and Consulting, Eurofund, Available at: http://www.eurofound.europa.eu/sites/default/files/ef1459en.pdf

Council of the European Union (2000) "Council Directive 2000/78/EC of 27 November 2000 establishing a general framework for equal treatment in employment and occupation", *Official Journal of the European Communities*, Available at: http://eur-lex.europa.eu/legal-content/EN/TXT/PDF/?uri=CELEX:32000L0078&from=EN

Dekkers-Sánchez, P. M., Wind, H., Sluiter, J. K., Frings-Dresen, M. H. W. (2010) "A Qualitative Study of Perpetuating Factors for Long-Term Sick Leave and Promoting Factors for Return to Work: Chronic Work Disabled Patients in Their Own Words," *Journal of Rehabilitation Medicine*, 42 (6): 544-552(9).

Dua, T., Garrido Cumbrera, M., Mathers, C. and Saxena, S. (2006) "Global Burden of Neurological Disorders: Estimates and Projections", in *Neurological Disorders: Public Health Challenges*, World Health Organization, Switzerland.

Economist Intelligence Unit (2012) "Never too Early: Tackling Chronic Disease to Extend Healthy Life Years", The Economist Intelligence Unit Limited, Available at: http://digitalresearch.eiu.com/extending-healthy-life-years/content/files/download/report/EIU-Abbott_TacklingChronicDisease_Report_final.pdf

Eikemo, T. A., Clare Bambra, C., Judge, K. And Ringdal K. (2008a) "Welfare state regimes and differences in self-perceived health in Europe: A multilevel analysis", *Social Science & Medicine,* 66 (2008): 2281-2295.

Eikemo, T.A., Huisman, M., Bambra, C. and Kunst, A.E. (2008b) "Health inequalities according to educational level in different welfare regimes: a comparison of 23 European countries", *Sociology of Health & Illness,* 30 (4): 565–582.

European Agency for Safety and Health at Work (EU-OSHA) (2007) "Work-related musculoskeletal disorders: Back to work report", Office for Official Publications of the European

Communities, Belgium, Available at: https://www.google.be/url?sa=t&rct=j&q=&esrc=s&source=web&cd=1&cad=rja&uact=8&ved=0ahUKEwin1dXn_pTKAhVJ1RQKHUFECyEQFggfMAA&url=https%3A%2F%2Fosha.europa.eu%2Fsites%2Fdefault%2Ffiles%2Fpublications%2Fdocuments%2Fen%2Fpublications%2Freports%2F7807300%2FTE7807300ENC_-_Work-related_musculoskeletal_disorders-_Back_to_work.pdf&usg=AFQjCNEfkXzEVKVaeI67iHfkujFVuMT7Kg&sig2=7KrdXbN9jIzKK5b1KpiLOg

European Association of Persons with Disabilities (EASPD) (2014) "Social Welfare Systems Across Europe", SensAge, Belgium, Available at: http://www.easpd.eu/sites/default/files/sites/default/files/SensAge/d4-social_welfare_systems_across_europe.pdf

European Commission (2010) "Europe 2020: A strategy for smart, sustainable and inclusive growth, Communication from the Commission", COM(2010), Brussels, Available at: http://eur-lex.europa.eu/LexUriServ/LexUriServ.do?uri=COM:2010:2020:FIN:EN:PDF

European Commission (2013) "Social economy and social entrepreneurship: Social Europe guide, Volume 4", Directorate-General for Employment, Social Affairs and Inclusion, European Union, Available at: http://ec.europa.eu/social/BlobServlet?docId=10010&langId=en

European Commission (2015) "Employment and Social Developments in Europe 2014", European Union, Brussels, Available at: http://ec.europa.eu/social/BlobServlet?docId=13404&langId=en

European Parliament (2008) "Resolution of 15 January 2008 on the Community strategy 2007– 2012 on health and safety at work", (2007/2146(INI)) European Parliament, Available at: http://www.europarl.europa.eu/sides/getDoc.do?pubRef=-//EP//NONSGML+TA+P6-TA-2008-0009+0+DOC+PDF+V0//EN

Eurostat (2010) "Health and safety at work in Europe (1999–2007): Statistical Portrait", European Union Publications Office, Available at: http://ec.europa.eu/eurostat/documents/3217494/5718905/KS-31-09-290-EN.PDF/88eef9f7-c229-40de-b1cd-43126bc4a946

Fenger, H. (2007) "Welfare regimes in central and Eastern Europe: incorporating post-communist countries in a welfare regime typology", *Contemporary Issues and Ideas in Social Sciences,* 3(2): 1-30

International Diabetes Federation (IDF) (2013) *Diabetes Atlas*, 6th edition, International Diabetes Federation, Brussels. Available at: https://www.idf.org/sites/default/files/EN_6E_Atlas_Full_0.pdf

International Labour Office (ILO) (2014) "World Social Protection Report 2014/15: Building economic recovery, inclusive development and social justice", Geneva: ILO, Available at: http://www.ilo.org/wcmsp5/groups/public/---dgreports/---dcomm/documents/publication/wcms_245201.pdf

Kessler, R. C. and Ustun, B. (eds.) (2008) *The WHO World Mental Health Surveys: Global Perspectives on the Epidemiology of Mental Disorders*, Cambridge University Press, New York, First Edition.

Leonardi, M., Steiner, T. J., Scher, A. T. and Lipton R. B. (2005) "The global burden of migraine: measuring disability in headache disorders with WHO's Classification of Functioning, Disability and Health (ICF)", *J Headache Pain* (2005)6:429–440.

Luttmann, A., Jager M. and Griefahn B. (2003) "Preventing Musculoskeletal Disorders in the Workplace, Protection Workers' Health Series No. 5", WHO Library Cataloguing-in-Publication Data, Available at: http://www.who.int/occupational_health/publications/en/oehmsd3.pdf

Midttun A., Gautesen, K. and Gjølberg, M. (2006) "The political economy of CSR in Western Europe", *Corporate Governance: The international journal of business in society*, 6(4): 369 – 385.

OECD (2003) "Transforming Disability into Ability: Policies to Promote Work and Income Security for Disabled People", OECD Publications, France, Available at: http://www.keepeek.com/Digital-Asset-Management/oecd/social-issues-migration-health/transforming-disability-into-ability_9789264158245-en#page216

OECD (2007) "New Ways of Addressing Partial Work Capacity: OECD Thematic Review On Sickness, Disability And Work Issues" Paper And Progress Report, OECD, Available at: http://www.oecd.org/social/soc/38509814.pdf

OECD (2010) *Sickness, Disability and Work: Breaking the Barriers. A Synthesis of Findings Across OECD Countries*, OECD Publishing, France, Available at: http://ec.europa.eu/health/mental_health/eu_compass/reports_studies/disability_synthesis_2010_en.pdf (Book)

OECD (2014) *Health at a Glance: Europe 2014*, OECD Publishing, France, Available at: http://ec.europa.eu/health/reports/docs/health_glance_2014_en.pdf

Paauw, D. (2015) “Comprehensive Care of the Patient with Chronic Illness”, *Medical Clinics of North America*, 99(5): i

Pohjola A. (2001) “Health problems and long-term unemployment”, *Social Work and Health Care,* 34(1-2):101-12.

Popova, Y. and Kozhevnikova, M. (2013) “Interdependence of HDI and budget redistribution within the Scandinavian and Continental Social Models”, *Economics & Management* 18(3), 562-575.

Saltman, R. B., Calltorp, J. and de Roo, A. (2011) “Health Sector Innovation and Partnership”, in: Health Reform: Meeting the Challenge of Ageing and Multiple Morbidities, OECD Publishing, France, Available at: http://www.oecd.org/els/health-systems/49151107.pdf

Social Protection Committee (2015) “Review of recent social policy reforms: 2015”, Report of the Social Protection Committee, Luxembourg: Publications Office of the European Union, Available at: http://ec.europa.eu/social/BlobServlet?docId=14927&langId=en

Schneider, E. and Irastorza, X. (2010) “OSH in figures: Work-related musculoskeletal disorders in the EU — Facts and figures”, European Risk Observatory Report, European Agency for Safety and Health at Work (EU-OSHA). Available at: https://osha.europa.eu/en/tools-and-publications/publications/reports/TERO09009ENC

Schuring Merel, Lex Burdorf, Anton Kunst, Johan Mackenbach (2007) “The effects of ill health on entering and maintaining paid employment: evidence in European countries”, *Journal of Epidemiology and Community Health*, 61(7): 597-604.

Taskila, T., Gulliford, J., and Bevan S. (2013) “Returning to Work: Cancer survivors and the Health and Work Assessment and Advisory Service”, Work Foundation, Available at http://www.theworkfoundation.com/downloadpublication/report/332_final%20returning%20to%20work%20150313.pdf

United Nations (UN) (2011) “UN General Assembly Resolution A/RES/66/2 of 19 September 2011, Political Declaration on the Prevention and Control of Non-communicable Diseases”, UN, Available at: http://daccess-dds-ny.un.org/doc/UNDOC/GEN/N11/458/94/PDF/N1145894.pdf?OpenElement

World Health Organization (WHO) (2002) “National cancer control programmes: policies and managerial guidelines”, 2nd ed. Printed in Italy, Available at http://apps.who.int/iris/bitstream/10665/42494/1/9241545577.pdf

World Health Organization (WHO) (2011) “Atlas of headache disorders and resources in the world 2011”, WHO, Italy, Available at:

http://www.who.int/mental_health/management/who_atlas_headache_disorders.pdf?ua=1

World Health Organization (WHO) (2013) "Mental Health Action Plan 2013-2020", WHO, Geneva, Available at: http://apps.who.int/iris/bitstream/10665/89966/1/9789241506021_eng.pdf?ua=1

World Health Organization (WHO) (2014) "Global Status Report on Noncommunicable Diseases 2014", WHO, Geneva, Available at: http://apps.who.int/iris/bitstream/10665/148114/1/9789241564854_eng.pdf?ua=1

World Health Organization (WHO) (2015) "Cardiovascular diseases (CVDs)", Fact sheet N°317, updated on January 2015 http://www.who.int/mediacentre/factsheets/fs317/en/

World Health Organization (WHO) (2016) "Chronic respiratory diseases", WHO, Webpage, Available at http://www.who.int/respiratory/en/

Zheltoukhova, K. (2013) "Musculoskeletal Disorders and Work: Results of a survey of individuals living with Musculoskeletal Disorders in six European countries", The Work Foundation, London, Available at: http://www.fitforworkeurope.eu/FFW_Patient_Survey_FINAL_2013.pdf

Annex 1: Questionnaire and interview respondents

Questionnaire respondents

	Organisation	Position
AT	Österreichische Diabetikervereinigung	Chairwoman at national level
	Österreichischer Herzverband (Landesverband Kärnten)	President at regional level in Kärnten (retired)
	Österreichische Krebshilfe Wien	Managing director of Krebshilfe
	SHÖ Schlaganfallhilfe Österreich	Chairwoman SHÖ - Schlaganfallhilfe Österreich (stroke help Austria) (retired)
	Pro mente oberösterreich	Head of 'pro mente arbeit' (branch of the organisation addressing issues around mental health issues & work/profession)
	Ottirol umbrella organisation for support groups	Head of regional umbrella association of support groups (Osttirol)
	Support group headaches	Assistant of managing director (head of support group)
	Behindertenanwaltschaft	"Behindertenanwalt" on national level
	Dachverband Selbsthilfe Kärnten	Managing director
CZ	General Labour Office of Czech Republic	Representative
	NGO Cerebrum	Representative
	Government Committee for people with disabilities in Czech Republic	Representative
	Department of Rehabilitation Medicine	Representative
	Occupational therapists	Occupational therapists
	Department of Rehabilitation Medicine	Head physician
	First Medical Faculty	Head of education
	NGO Rehalb	Director
	Prague committee of wheelchairs users	User
	Department of Rehabilitation Medicine, founder of prevocational rehabilitation	Former head
DE	Südwestfälische Industrie- und Handelskammer zu Hagen (SIHK)	Inclusion consultant

	CBP - Caritas Behindertenhilfe und Psychiatrie e.V.	Lawyer
	Sozialverband Deutschland SoVD	Lawyer
	Bundesarbeitsgemeinschaft Integrationsfirmen e. V. (bag if)	Social worker, managing director
	Federal Employment Agency, medical service	Physician
EL	Department of Special Education, University of Thessaly	Professors and Researcher A
	Manpower Employment Organisation - Employment Office for Special Social Groups	Job consultant
	The Greek Ombudsman	Scientific staff - Human Rights Department
	Panhellenic Union for the Psychosocial Rehabilitation and Work Integration	Employment counsellor
	Multiple Sclerosis Panthessalic Union	Secretarial and administrative support - unemployed
	Panhellenic Federation of Unions-Associations of persons with Diabetes Mellitus	Biologist
	Bone Health Society "Butterfly"	General Practitioner
	Association "Hellenic Pulmonary Hypertension"	President of the Association "Hellenic Pulmonary Hypertension"
	Social Cooperative Firm "La petite cantine"	Representative of a Social Cooperative Firm
	Greek Anticancer Society	General Practitioner
ES	Instituto de Mayores y Servicios Sociales (Institute for the Elderly and Social Services IMSERSO)	Worker of the State General Administration
	Asociación Española Contra el Cáncer (aecc) (Spanish Association Against Cancer (AECC))	Social worker
	Intecserveis CET (Fundació Germà Benito Menni) (Brother Benito Menni Foundation)	Social worker
	PSSJD	Job developer / Labor insertor
	PSSJD	Neurologist
	Provincial Directorate of the National Social Security Institute	Provincial Deputy Director of Support and Information of the National Institute of Social Security Barcelona
	APACOR – Asociación De Pacientes Coronarios (Association of coronary patients)	Retired. Voluntareer
	Fundación Lovexair (Lovexair foundation)	Psychologist
	PSSJD	Social Worker
	Servicio Andaluz de Saluz (SAS) (Andalusian Healthcare Service)	Doctor
	La Paz Hospital	Traumatologist
	Virgen del Rocio Hospital	Neurologist
	Foundation Carmen Pardo - Valcarce	Director of Employment services

	Hospital La princesa	MD
	Hospital La princesa	MD, Respiratory unit
	UAM and Hospital La princesa	Psychiatrist
	Hospital La princesa	MD
IT	Fondazione IRCCS, Istituto Neurologico Carlo Besta	Social worker
	LEDHA (Disabled persons's rights Association)	Social worker
	A.O. Luigi Sacco - Hospital	Psychiatric Consultant
	A.O. Mellino Mellini - Hospital	Mental Health Department Director
	A.O. San Carlo Borromeo - Hospital	Neurologist, Hospital Unit Director
	ADPMI, Association for patients with diabetes in Milan - CLAD, Coordination for associations of patients with diabetes in the Lombardy Region	President of the Milan Diabetes Association and Coordinator
	Rehabilitation Centre Villa Beretta - Ospedale Valduce	Social worker
	AIMaC, Italian Association for patients with cancer, caregivers and friends	Vice President
	U.O. Broncopneumologia, A.O. di Busto Arsizio - Tradate - Saronno (Hospital, Bronchopneumology Unit)	Healthcare assistent
	AICCA Onlus, Associazione Italiana dei Cardiopatici Congeniti Adulti. (Italian association of congenital heart patients)	Counselor - Peer Counselor
NO	NAV Buskerud	Advisor for persons with chronic diseases, HR management
	NAV Hamar	Advisor

Interview respondents

	Organisation	Occupation
AT	Austrian Chamber of Labour (Arbeiterkammer), Department of Labour Market and Integration	Expert
	Austrian Chamber of Labour (Arbeiterkammer), Department of Social Insurance	Expert
	Anwaltschaft für Menschen mit Behinderung	Expert
CZ	CEREBRUM - Brain Injured and their Families Czech Republic	Representative
	The Labour Office of the Czech Republic – General directorate	Representative
	Government committee for persons with disabilities	Head

DE	Corporate integration management (Betriebliches Eingliederungsmanagement), Klinikum Großhadern	Head of the department
	Medical Advisory Service of the statutory health insurer in Bavaria	Regional Chair of the Health Policy Working Group
EL	EDRA Social Cooperative Action for Vulnerable Groups	Representatives
	National Confederation of People with Disabilities	Member
	Special Service for Social Inclusion and Social Economy (EYKEKO)	Representative
ES	Madrid's health area	Psychiatrist, responsible for the continuity
	User, owner of enterprise of breast and feat prosthesis	Psychologist
	Mental Health Regional Office of Madrid	Deputy Director
	Patient with Obsessive Compulsory Disease	User
IT	Ministry of Labour and Social Policy	Representative
	A&I Onlus	Member
	AISM (Italian Multiple Sclerosis Society)	User
NO	"Stop the Discrimination"	Activist in disabled people's movement
	Norwegian Welfare Administration (NAV)	Job counsellor
	Norwegian Welfare Administration (NAV)	Sociologist
PL	Marschal's Office of Malopolska Voivodeship	Representative
	Foundation Activation	Representative
	Polish Association Of Disabled People, University of John Paul II in Krakow	Psychologist and career counselor
SI	Cveto Uršič	Representative
	Slovenian Association of Vocational Rehabilitation Service Providers	President of the Association
	Association of Persons with Disabilities	Vocational rehabilitation service user
UK	Remploy	Representatives

Annex 2: Questionnaire template

Section A. General Information

1) Respondent Information

1.1. Name (optional): ………………………………………………………………………………….

1.2. Occupation: ………………………………………………………………………………….

1.3. What is your expertise or experience in the domain of employability of persons with chronic health conditions/disabilities (and/or in the field of professional (re-)integration of persons with chronic diseases/disabilities)?

……

……

2) Organization details

2.1. Name of your organization ………………………………………………………………………………….

2.2. City and country ………………………………………………………………………………….

2.3. Website ………………………………………………………………………………….

2.3. Type of organisation:

- ☐ Public authority
- ☐ Non-profit service provider
- ☐ For-profit service provider
- ☐ Research institute
- ☐ Other ………………………………………………………………………………….

2.4. Please list the services that your organisation provides in the field of employability of persons with chronic diseases (and/or in the field of professional integration/reintegration of persons with chronic diseases).

Use this space to describe your services, mention who the recipients of your services are, specify whether your services are targeted at certain categories of chronic diseases and include any additional information if you find it relevant

……

……

……

Section B. Information on national-level legislation regulating the employment of persons with reduced work capacity

3) National legislation on employment of persons with reduced work capacity

3.1. How does the government in your country **assess** whether a person has a reduced capacity to work?

………
………
………

3.2. Does the government provide any <u>disability pensions</u> for workers with chronic health conditions/disability that cause them to perform at reduced work capacity?

☐ Yes
☐ No
☐ I do not know

Use this space if you would like to provide additional information to explain your answer

………
………

3.3. Does the government provide any <u>benefits</u> for workers with chronic health conditions/disability that cause them to perform at reduced work capacity?

☐ Yes
☐ No
☐ I do not know

Use this space if you would like to provide additional information to explain your answer

………
………

3.4. If your answer in Question 4.2. is "yes", please list the **conditions** under which these persons can receive the benefits?

………
………

3.5. Persons with reduced work capacity in your country:

☐ are **required by law** to prove that they have sought employment in the labour market before applying for disability or unemployment benefits
☐ **have a right** to seek employment and are entitled for disability or unemployment benefits
☐ Other ……
☐ I do not know

3.6. In your country, are there legal provisions on the **employment** of **persons with reduced work capacity?**

☐ Yes
☐ No
☐ I do not know

3.7. If yes, please specify in which of the following types of legislation these provisions are included:

	**Legislation**	**yes**	**no**	**I don't know**
a	legislation on non-discrimination	☐	☐	☐
b	legislation on employment protection	☐	☐	☐
c	legislation on occupational safety and health	☐	☐	☐
d	legislation on social security	☐	☐	☐
e	legislation on healthcare	☐	☐	☐
f	legislation on disability	☐	☐	☐

☐ Other ………………………………………………………………………………………

☐ I do not know

Use this space if you would like to provide additional information to explain your answer
………
………

3.8. Please indicate the status of the following rights of persons with reduced work capacity in employment in your country.

	**Rights**	**No legislation or policies**	**Binding law**	**Non-binding policies**	**Other**	**I do not know**
a	Right to physical workplace adaptation (reasonable accommodation)	☐	☐	☐	☐ ✏_ _ _ _ _ _ _ _ _ _ _ _ _ _	☐
b	Right to work schedule and working hours adaptation	☐	☐	☐	☐ ✏_ _ _ _ _ _ _ _ _ _ _ _ _ _	☐
c	Right to receive education and (re)training to obtain	☐	☐	☐	☐ ✏_ _ _ _ _ _ _	☐

	additional skills and qualifications				_ _ _ _ _ _ _	
d	Right to get support in job search (job coaching, human resources advice)	☐	☐	☐	☐ ✎_ _ _ _ _ _ _ _ _ _ _ _ _ _	☐
e	Right to paid or unpaid leave in order to undergo medical treatment while working	☐	☐	☐	☐ ✎_ _ _ _ _ _ _ _ _ _ _ _ _ _	☐
f	Right to internal redeployment	☐	☐	☐	☐ ✎_ _ _ _ _ _ _ _ _ _ _ _ _ _	☐
g	Right to ongoing human support	☐	☐	☐	☐ ✎_ _ _ _ _ _ _ _ _ _ _ _ _ _	☐
Use this space below to list any other employment-related rights of persons with reduced work capacity that exist in your country						

3.9. Is there a quota for employers in your country for hiring persons with disabilities?

☐ Yes, there is quota for public sector organisations only
☐ Yes, there is quota for private sector organisations only
☐ Yes, there is quota for both public and private sector organisations
☐ No
☐ I do not know

Use this space if you would like to provide additional information to explain your answer

....

....

3.10. Can employers who hire persons with reduced work capacity receive compensation or subsidies from the government for the possible loss in productivity?

☐ Yes
☐ No
☐ I do not know

Use this space if you would like to provide additional information to explain your answer

....

....

3.11. Is there national legislation on employment in which persons with **chronic diseases/disability** are singled out as a distinct group?

☐ Yes *(Please briefly explain the type of legislation)…………………………………………………………………………*

……
…..

☐ No

☐ I do not know

Use this space if you would like to provide additional information to explain your answer

……

……

3.12. Are there national laws on employment that **hinder** the professional (re-)integration of persons with reduced work capacity? Please explain your answer below:

……

……

……

3.13. Are there **gaps** in the national legislation between the employment needs of persons with reduced work capacity and legal provisions on their professional (re-)integration?

If yes, please describe the gaps below:

……

……

……

Section C. Information on disease/disability-specific legislation, schemes, and services

4) **Please select one group of persons with chronic conditions/disabilities for which you will provide your responses in the questionnaire:**

☐ Persons with **mental health issues**
☐ Persons with **neurological diseases (with a focus on headache disorders)**
☐ Persons with **metabolic disorders**
☐ Persons with **musculoskeletal disorders**
☐ Persons with **respiratory diseases**
☐ Persons with **cardiovascular diseases**
☐ Persons with **cancer**

From here on, the group that you have selected will be referred to as "persons from the selected group"

4.1. Based on your country's legislation, persons from the selected group**…**

☐ …can be legally recognized as unable to work
☐ …cannot be legally recognized as unable to work
☐ I do not know

4.2. Based on your legislation, when a person from the selected group is legally recognized as unable to work…

☐ … he or she has to undergo reassessment on a regular basis to maintain this status.
☐ … he or she does not have to undergo a reassessment to maintain this status.
☐ Other ………………………………………………………………………………………………
☐ I do not know

4.3. Your legislation provides measures allowing persons from the selected group to access:

☐ Sheltered work only
☐ Open labour market jobs, including sheltered work
☐ Open labour market jobs, excluding sheltered work
☐ I do not know

4.4. Is there legislation in your country that specifically focuses on the employment of persons from the selected group?

☐ Yes *(If yes, please explain the type and the objective of the legislation)*
……
……

……
………………………………

☐ No, persons from the selected group are not singled out but may be covered by the broader legislation on persons with disabilities.

☐ I do not know

4.5. Please name **legal acts, national strategies, national action plans or other national laws**, that specifically focus on the **professional (re-)integration** of persons from the selected group.

(Please briefly describe their nature and the main features of these laws. If there are no such laws, please put "N/A")

……
……
……

4.6. What **national employment programmes** are available in your country for the implementation of national policies on **professional (re-)integration** of persons from the selected group (e.g. related to education and training, financial subsidies, support in job seeking, workplace adjustments, etc.)?

……
……
……

4.7. Please fill in the following table to describe **services** known to you that are available in your country's for-profit, non-profit, and public sectors for professional (re-)integration of persons from the selected group.

**Brief description of service**	**Name of organisation that provides the service**	**Type of organisation (e.g. for-profit, non-profit, public)**	**Description of service recipients**
1.			
2.			
3.			
…			

Annex 3: Interview guidelines

Mapping of Existing Strategies on Professional (re-)integration of Persons with Chronic Diseases and Mental Health Issues (WP4)

Interview Design

The objective of this interview design is to provide guidance in interviewing three stakeholders (authorities, service providers, and users of services) as part of the WP4.

The interview design does not provide fixed sets of questions but rather identifies general areas of interest of the study.

You are flexible to formulate your own questions and adjust them depending on the local realities and responses of your interviewees. You are encouraged to use open-ended questions and ask additional and follow-up questions if you find them relevant.

1. Who is the respondent?

**A. For authority:**	**B. For service providers:**	**C. For users (self-advocates):**
i. Name of the authority<br>ii. To what governmental bodies is the authority subordinate?<br>iii. Main activities, services, responsibilities of authority<br>iv. Ways in which authority is involved in the employment (re-)integration of persons with chronic diseases/disorders (persons with a reduced work capacity)<br>v. Target group of the authority<br>vi. Scope of action (national, regional, local)	i. Name of the organisation<br>ii. Type of organisation (for-profit, non-profit, or public)<br>iii. Main activities, services, responsibilities of organisation<br>iv. Ways in which organisation is involved in the (re-)integration of persons with chronic diseases/disorders (persons with a reduced work capacity)<br>v. Main sources of funding (in relation to employment (re-)integration activities)<br>vi. Users of the organisation's services<br>vii. Scope of action (national, regional, local)	i. Name, age, gender, type of disease, disability.<br>ii. For self-advocates: main activities as a self-advocate?<br>iii. Ways in which he/she is involved in employment (re-)integration for persons with chronic diseases/disorders (persons with a reduced work capacity)?<br>iv. What is his/her experience in employment (re-)integration?<br>vii. Scope of action (national, regional, local)

2. What are the existing policies, programmes and services?	
**A.1. Are there any <u>government policies and strategies</u> that specifically regulate the employment of persons with chronic diseases/disorders?**<br><br>- <u>If yes:</u> Find out about various aspects of these policies and strategies.<br>*(title, objectives, provisions/eligibility, targeted groups, long-term vs short-terms, responsible organisation/office, type of sector, source of funding, potential partnerships, categories of chronic diseases covered, etc.).*<br><br>- <u>If no:</u> Are persons with chronic diseases/disorders included in other broader categories (e.g. persons with disabilities)? Which ones? Find out about these policies and strategies.<br>*(title, objectives, provisions/eligibility, targeted groups, long-term vs short-terms, responsible organization/office, type of sector, source of funding, potential partnerships, categories of chronic diseases potentially covered, etc.).*<br><br>**A.2. Is information about these policies and strategies accessible online and in English?**<br>*(URLs, keywords, names of online reports and publications, names of portals, etc.)*	**A.3. Respondent's opinion about policies and strategies identified by him/her:**<br>*(advantages and disadvantages, challenges and opportunities, adequacy of financial support by government, adequacy of coordination, possible gaps, cost-effectiveness, effectiveness in (re)integrating persons with chronic diseases/disorders into the open labour market)*<br><br>**A.4. Formal evaluation:**<br>Was the effectiveness of policies and strategies previously evaluated? If yes, provide information about the results of evaluation.
**B.1. Are there programmes and schemes aimed at promoting the employment (re-)integration of persons with chronic diseases?**<br><br>*(title, objectives, provisions/eligibility, targeted groups, long-term vs short-terms, responsible organisation/office, type of sector, source of funding, sheltered workshops vs open labour market, potential partnerships, categories of chronic diseases covered, etc.).*<br><br>**B.2. Is information about these programmes and schemes accessible online and in English?**<br>*(URLs, keywords, names of online reports and publications, names of portals, etc.)*	**B.3. Respondent's opinion about programmes and schemes identified by him/her:**<br>*(advantages and disadvantages, challenges and opportunities, adequacy of financial support by government, adequacy of coordination, possible gaps, cost-effectiveness, effectiveness in (re)integrating persons with chronic diseases/disorders into the open labour market)*<br><br>**B.4. Formal evaluation:**<br><br>Was the effectiveness of programmes and schemes previously evaluated? If yes, provide information about the results of evaluation.

C.1. Are there services facilitating the employment (re-)integration of persons with chronic diseases?

(title, objectives, provisions/eligibility, targeted groups, long-term vs short-terms, responsible organisation/office, type of sector, type of service provider (for-profit, non-profit or public organisations) source of funding, sheltered workshops vs open labour market, potential partnerships, categories of chronic diseases covered, etc.).

C.2. Is information about these services accessible online and in English?
(URLs, keywords, names of online reports and publications, names of portals, etc.)

C.3. Respondent's opinion about services identified by him/her:
(advantages and disadvantages, challenges and opportunities, adequacy of financial support by government, adequacy of coordination, possible gaps, cost-effectiveness, effectiveness in (re)integrating persons with chronic diseases/disorders into the open labour market)

C.4. Formal evaluation:
Was the effectiveness evaluated? If yes, provide information about the results of evaluation?

Annex 4: Statistical data on prevalence of and mortality from chronic diseases

Prevalence of chronic diseases

According to the EU-SILC survey, around 32.0% of EU-28 residents of all ages and both sexes declared having a long-standing illness or health problem in 2013, which was 1.7 percentage points higher than in 2005 (30.2%)[66]. Furthermore, 8.3% of EU-28 residents declared having severe long-standing limitations, and 18.2% reported some long-standing limitations in usual activities due to health problems[67].

The 2011 LFS data, the most common longstanding health condition reported by EU citizens was found to be MSDs[68] (41%), followed by CVDs[69] (13.4%), psychosocial problems[70] (8.9%), respiratory diseases[71] (6.1%), neurological conditions[72] (4.9%), diabetes (4.8%), cancer (2.2%) and other[73] (18.7%) (Figure 7). Among people who have more than one longstanding disease, MSDs appear to be most widespread also: it is reported as the second main longstanding health condition by about 41.3% of people. Psychosocial problems come second (12%) (Figure 7).

---

[66] Source: Eurostat: hlth_silc_05
(http://appsso.eurostat.ec.europa.eu/nui/show.do?dataset=hlth_silc_05&lang=en)
[67] Source: Eurostat: hlth_silc_07
(http://appsso.eurostat.ec.europa.eu/nui/show.do?dataset=hlth_silc_07&lang=en)
[68] This category includes problems with arms or hands; legs and feet; and back and neck (including arthritis or rheumatism),
[69] This category includes heart, blood pressure or circulation problems
[70] This category includes chronic anxiety, depression and other mental, nervous or emotional problems.
[71] This category includes chest or breathing problems, including asthma and bronchitis.
[72] This category includes epilepsy (including fits) and severe headache such as migraine.
[73] This category includes skin conditions, including allergic reactions and severe disfigurement; stomach, liver, kidney or digestive problems; learning difficulties (reading, spelling or math disability); other progressive illnesses (which include multiple sclerosis, HIV, Alzheimer's disease, Parkinson's disease); other longstanding health problems.

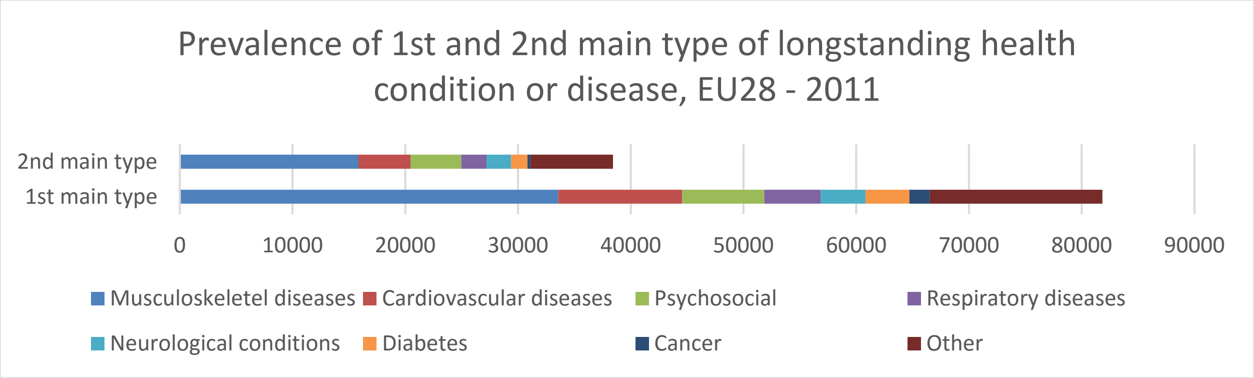

Figure 7. *The first and second main type of longstanding health condition or disease, EU-28 (2011), all persons of 20-64 years of age. Source: Eurostat, 2011 LFS special module on disability.*

Mortality from chronic diseases

Chronic diseases are largely responsible for mortality in Europe. According to WHO Global Health Estimates, seven disease categories considered in this study account for 70-80% of all years of life lost (YLL) in 2012 in 28 EU countries plus Norway and Switzerland (Figure 8). As seen from Figure 8, two main contributors to deaths are cardiovascular and malignant neoplasms, or cancer. MSDs, on the other hand, accounted only for a smaller share of YLL in 2012.

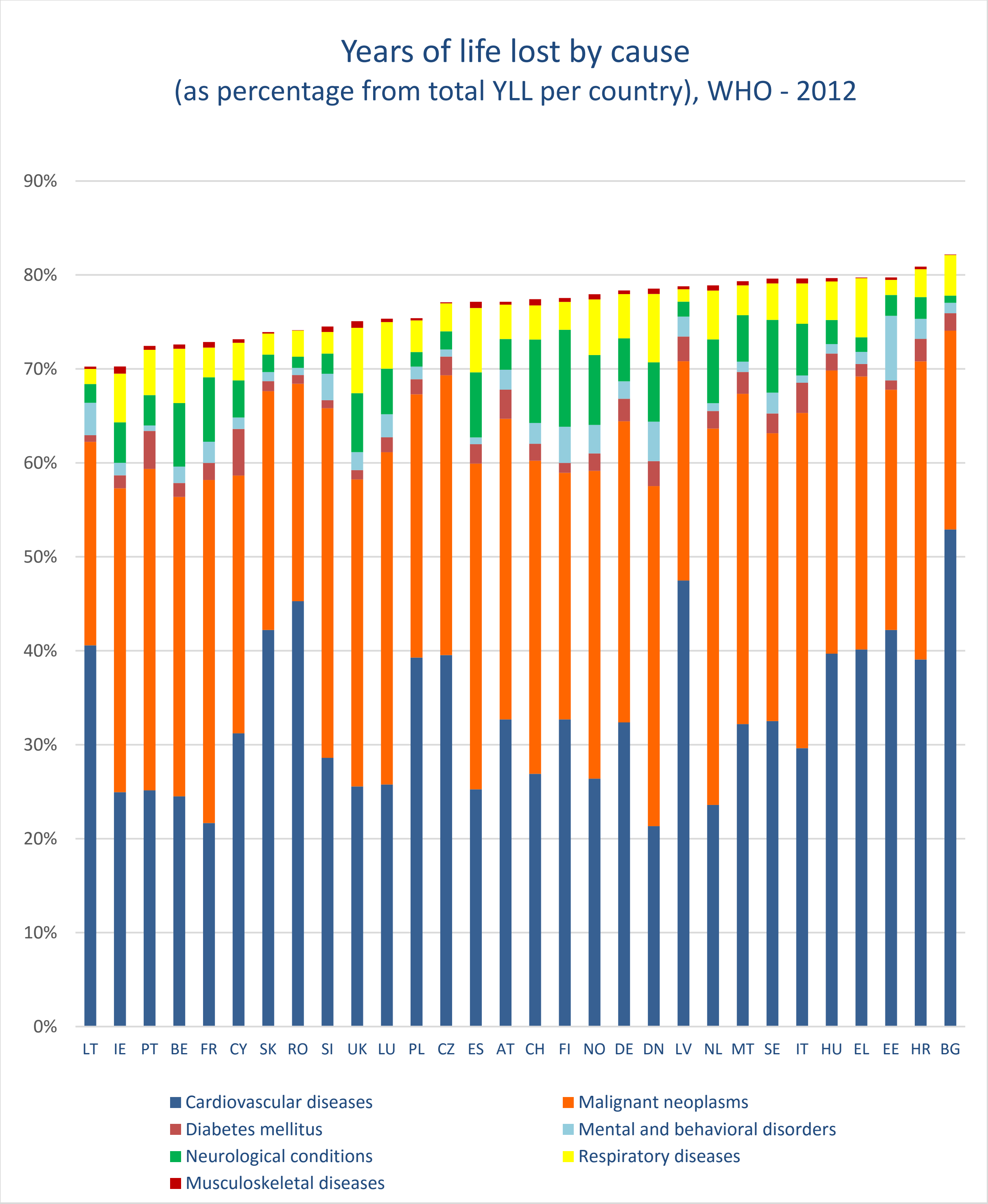

Figure 8. *YLL per country and per disease category, persons of all age groups and both sexes. Source: WHO Health Estimates.*

According to WHO estimates, cancer alone was responsible for almost 29.3 million years of life lost in 2012 in 28 EU countries plus Norway and Switzerland, which is equivalent to nearly 31.8% of all-cause deaths, followed by CVDs that are responsible for nearly 29.2 million YLL[74] (Figure 9).

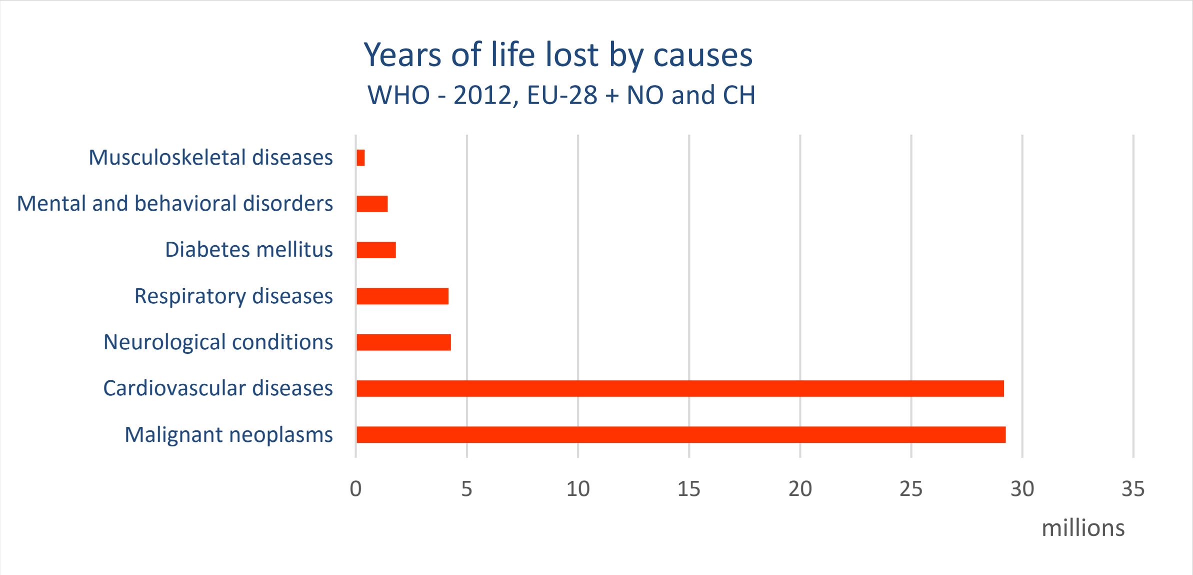

Figure 9. *Comparison of chronic disease categories based on their contribution to years of life lost (YLL), Source: WHO Health Estimates.*

In terms of estimated deaths caused by NCDs, CVDs appear to take the lead, accounting for an estimated 1.9 million deaths (38.9% of all deaths) in 2012 in EU-28 plus Norway and Switzerland, whereas cancer accounted for nearly 1.4 million deaths in 2012, which constitutes 27.2% of all deaths (Figure 7). Out of all NCD categories considered in this study, MSDs account for the smallest amount of deaths, according to WHO data.

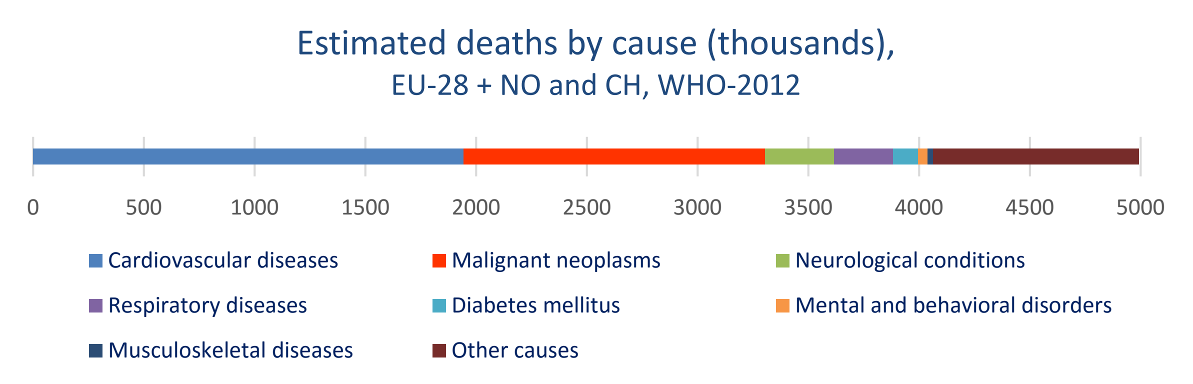

Figure 7*. Causes of death by categories of chronic diseases for persons of all age groups and both sexes. Source: WHO Health Estimates*

[74] Source: WHO Global Health Estimates, 2012, available at http://www.who.int/entity/healthinfo/global_burden_disease/GHE_YLL_2012_country.xls?ua=1 .

The death rate due to chronic diseases[75] in the EU-27 was 11.6% in 2009, with CEE countries in general accounting for higher death rates and Nordic states showing lower rates (Figure 8).

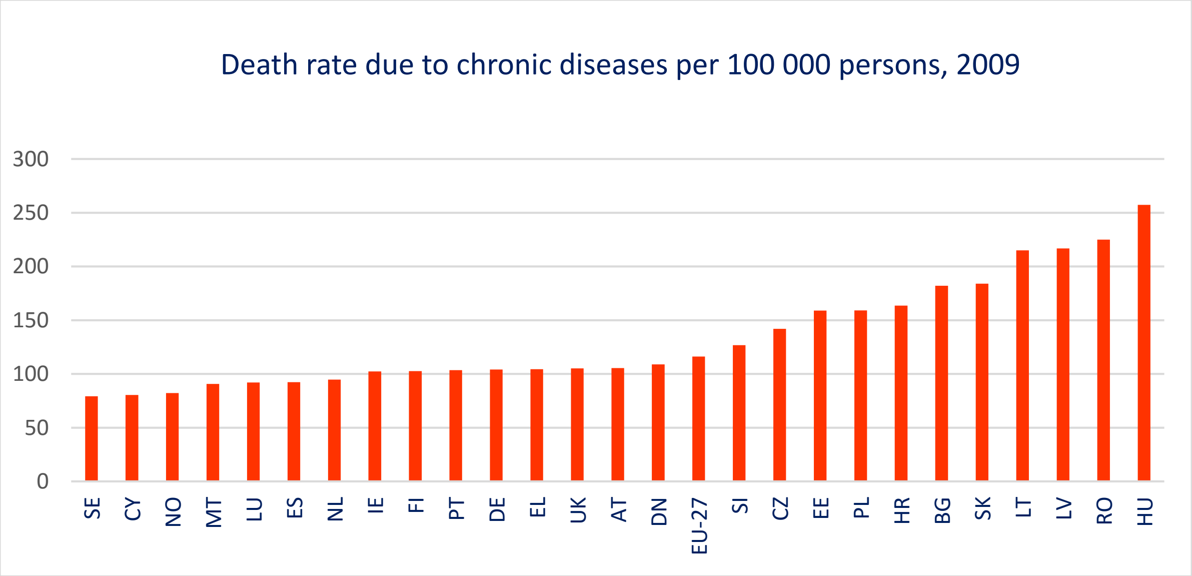

Figure 8*. Death rate due to chronic diseases (tsdph210), per 100,000 persons in 2009, Source: Eurostat*

[75] The indicator is defined as the standardised death rate of certain chronic diseases for persons aged less than 65 years. The following diseases have been considered: malignant neoplasms, diabetes mellitus, ischemic heart diseases, cerebrovascular diseases, chronic lower respiratory diseases, and chronic liver diseases.

Annex 5: Employment rate for chronic disease sub-categories

Categories of chronic disease	Sub-categories of chronic disease	Not employed	Employed
MSDs	Problems with arms or hands (which includes arthritis or rheumatism)	55.1%	44.9%
	Problems with legs or feet (which includes arthritis or rheumatism)	60.7%	39.3%
	Problems with back or neck (which includes arthritis or rheumatism	51.3%	48.7%
Neurological disorders	Epilepsy (including fits)	76.0%	24.0%
	Severe headache such as migraine	43.6%	56.4%
Respirato	Chest or breathing problems, including asthma and bronchitis	61.9%	38.1%

ry disorders			
Metabolic disorders	Diabetes	70.4%	29.6%
Cancer	Cancer	70.8%	29.2%
Cardiovascular disorders	Heart, blood pressure or circulation problems	71.7%	28.3%
Psycho-social probl	Chronic anxiety	68.4%	31.6%
	Depression	76.4%	23.6%
	Other mental, nervous or emotional problems	79.4%	20.6%

ems			
Other	Skin conditions, including allergic reactions and severe disfigurement	48.4%	51.6%
	Stomach, liver, kidney or digestive problems	64.1%	35.9%
	Learning difficulties (reading, spelling or math disability)	73.8%	26.2%
	Other progressive illnesses (which include multiple sclerosis, HIV, Alzheimer's disease, Parkinson's disease)	69.5%	30.5%
	Other longstanding health problems	62.4%	37.6%

Annex 6: Descriptive statistics for seven disease categories

Labour market participation of people with long-standing health conditions or diseases. Evidence from the Labour Force Survey 2011 - Special module on disability

The EU LFS is conducted in the 28 EU Member States and in a few other countries. It is a large household sample survey providing quarterly results on labour participation of people aged 15 and over, as well as on persons outside the labour force. The target population is composed of people living in private households, excluding those living in collective households or in institutions. The national statistical institutes are responsible for selecting the sample, preparing the questionnaires, conducting the face-to-face interviews with households, and forwarding the results to the statistical office of the European Union Eurostat.

Standard EU LFS questionnaires do not contain any questions on health or disability. However, each year a special block of questions or so-called "ad hoc module" is used to obtain more detailed information on a specific subject. In 2011 the topic was health and disability.

Many of the questions in the disability module are used to define a target group of people with a long-standing health condition or disease in order to allow for comparison of this group with the other respondents on the core variables of the LFS. To that end respondents are asked to indicate *their most important and second most important* long-standing health problem or disease in a fixed list of 18 items. They are also asked whether they have problems performing 10 listed activities of daily living (ADL) and whether they experience limitations in the amount of work (i.e. the number of hours) or the type of work they could do or in getting to and from work. Based on the lists of health problems and of ADL problems and on the 3 work-related limitations, a group of people with work limitations (PWL) caused by a long-standing health condition or an ADL problem" is identified.

As part of the EU project *Pathways*. *Participation to Healthy Workplaces And inclusive Strategies in the Work Sector* a questionnaire was sent to professionals in participating countries to collect information on labour market programmes for people with 7 types of diseases: mental health issues, MSDs, metabolic disorders, CVDs, respiratory diseases, neurological diseases (severe headaches) and cancer.

In the following analysis the data from the EU LFS ad hoc module on disability (EU-28) have been regrouped according to 6 of these categories, cancer being left *aside due to lack of data. Because our focus is on work, the population has been restricted to people aged* 20-64.

More EU LFS results on disability are available on the Eurostat website:

- http://ec.europa.eu/eurostat/statistics-explained/index.php/Disability_statistics_-_labour_market_access
- http://ec.europa.eu/eurostat/statistics-explained/index.php/Disability_statistics
- http://ec.europa.eu/eurostat/statistics-explained/index.php/EU_labour_force_survey_-_ad_hoc_modules

1. Mental health issues

Three LFS items - *chronic anxiety* (13), *depression* (14), and *other mental, nervous or emotional problems (15)* - can be combined into the category "mental health issues", each contributing to it for about one third.

3.3% of all the respondents report mental health issues as first or second most important long-standing health condition or disease; for 40% of them this is their sole health problem. Half of these respondents - 1.7% of all respondents - report limitations in work. About one third have mental health issues as sole problem.

The following figures relate to all the people with mental health problems, whether they have other health problems or not.

Table 1. People with mental health issues and work limitations by sex

Sex	Mental health issues	No mental health issues	Mental health issues + work limitations	All others
Male	40.9	50.1	42.1	49.9
Female	59.1	49.9	57.9	50.1
Total %	100.0	100.0	100.0	100.0
N (sample)	10 200	295 000	5 100	300 000

Eurostat – LFS2011 – Special module on disability – EU28 – 20-64 years

59% of all the people with mental health issues are female, compared to half of those with no such issues. Among people with mental health issues experiencing limitations in work 58% are female, compared to 50% among all others.

Table 2. People with mental health issues and work limitations by age

Age	Mental health issues	No mental health issues	Mental health issues + work limitations	All others
20-29	14.1	20.9	12.7	20.8
30-39	19.4	23.0	18.3	22.9
40-49	26.7	24.3	27.2	24.3
50-59	28.7	21.8	31.0	21.8
60-65	11.1	10.0	10.9	10.0
Total %	100.0	100.0	100.0	100.0
N (sample)	10 200	295 000	5 100	300 000

Eurostat – LFS2011 – special module on disability – EU28 – 20-64 years

Table 3 clearly indicates that mental health issues occur in every age group but are more common among people aged 40 and over. The same pattern shows in the subgroup of people with mental health issues experiencing work limitations.

Table 3. People with mental health issues and work limitations by education attainment level

Education attainment	Mental health issues	No mental health issues	Mental health issues + work limitations	All others
Low	41.9	25.4	47.5	25.5
Medium	39.5	48.9	38.8	48.8
High	18.7	25.7	13.7	25.7
Total %	100.0	100.0	100.0	100.0
N (sample)	10 200	295 000	5 100	300 000

Eurostat – LFS2011 – special module on disability – EU28 – 20-64 years

The education attainment level of people with mental health issues is much lower than that of people with no such issues: 41.9% versus 25.4% have a low level of education. The gap is even wider for those also reporting work limitations: 47.5% vs. 25.5%.

Table 4. People with mental health issues and work limitations by labour market status

Labour market status	Mental health issues	No mental health issues	Mental health issues + work limitations	All others
Employed	40.9	69.6	25.6	69.4
Unemployed	10.3	6.9	9.2	7.0
Inactive	48.8	23.5	65.1	23.7
Total %	100.0	100.0	100.0	100.0
N (sample)	10 200	295 000	5 100	300 000

Eurostat – LFS2011 – special module on disability – EU28 – 20-64 years

The employment rate is much lower among people with mental health issues than among people with no such problems (28.7 percentage points lower); among people with mental health issues and

work limitations it is even 43.8 percentage points lower. By contrast, the unemployment rate and especially the inactivity rate is always much higher.

Table 5. Active people with mental health issues and work limitations by employment status

Employment status	Mental health issues	No mental health issues	Mental health issues + work limitations	All others
Full-time	71.0	82.1	56.0	82.0
Part-time	29.0	17.9	44.0	18.0
Total %	100.0	100.0	100.0	100.0
N (sample)	4 000	205 000	1300	208 300

Eurostat – LFS2011 – special module on disability – EU28 – 20-64 years

A large majority of active people with mental health issues having work limitations are employees (85%), 14% are self-employed (with or without employees) and 1% are family workers. Their employment status is not significantly different form that of all other people (figures not shown in tables).

Active people with mental health issues are more often in part-time work than people who don't have such problems (29% - 18% = 11 percentage points difference). The difference is even greater among those with work limitations (44% - 18% = 26 pp). Of the active people with mental health issues 19% attribute part-time work to disability or illness, compared to 4% of the people with no such issues. Among those with work limitations, the figures are 38% compared to 4%.

People with mental health issues also more often hold a temporary job but the difference with other people is not significant (14% vs. 13% for all and 15% vs. 13% for those with work limitations).

A very high number of people with mental health issues experiencing work limitations are limited in the type of work (89%) or the amount of work (79%) they can perform. Nearly half (49%) have problems getting to and from work. However it should be kept in mind that some of these limitations may be caused by other long-standing health conditions or diseases.

Some 40% of people with mental health issues experiencing work limitations report a need for special working arrangements, 26% need personal assistance and 17% need workplace adaptations, with the proviso that some of these needs may be related to other long-standing health conditions or diseases.

2. Musculoskeletal disorders (MSD)

Three LFS items - *problems with arms or hands (which includes arthritis or rheumatism)* (1), *problems with legs or feet (which includes arthritis or rheumatism)* (2), and *problems with back or neck (which includes arthritis or rheumatism)* (3) - can be combined into the category "musculoskeletal disorders". Problems with back or neck is the largest subcategory (about 50%), problems with arms or hands the smallest (about 20%).

A total of 13.4% of all the respondents report MSDs as first or second most important long-standing health condition or disease; for 60% of them it is the sole health problem. About half of these respondents – 6.3% of all respondents – report limitations in work. Just over half have MSDs as a sole problem.

The following figures relate to all the people with MSDs, whether they have other health problems or not.

Table 6. People with MSDs and work limitations by sex

Sex	MSDs	No MSD	MSD + work limitations	All others
Male	46.1	50.3	44.4	50.1
Female	53.9	49.7	55.6	49.9
Total %	100.0	100.0	100.0	100.0
N (sample)	41 000	264 000	19 000	286 000

Eurostat – LFS2011 – special module on disability – EU28 – 20-64 years

About 54% of the respondents with MSDs are female, compared to half of those without. Among people with MSD experiencing limitations in work, 56% are female compared to 50% of all others.

Table 7. People with MSDs and work limitations by age

Age	MSDs	No MSD	MSD + work limitations	All others
20-29	7.3	22.8	5.8	21.7
30-39	14.1	24.2	11.5	23.6
40-49	24.1	24.4	22.5	24.5
50-59	35.4	19.9	38.6	20.9
60-65	19.1	8.7	21.7	9.3
Total %	100.0	100.0	100.0	100.0
N (sample)	41 000	264 000	19 000	286 000

Eurostat – LFS2011 – special module on disability – EU28 – 20-64 years

Table 7 clearly indicates that MSDs occur in every age group but are more common among people aged 50 and over. The same pattern shows in the subgroup of people with MSD experiencing work limitations.

Table 8. People with MSDs and work limitations by education attainment level

Education attainment	MSDs	No MSD	MSD + work limitations	All others
Low	35.6	24.4	40.6	24.9
Medium	46.7	48.9	46.3	48.7
High	17.7	26.7	13.1	26.3
Total %	100.0	100.0	100.0	100.0
N (sample)	41 000	264 000	19 000	286 000

Eurostat – LFS2011 – special module on disability – EU28 – 20-64 years

The education attainment level of people with MSDs is lower than that of people with no such disorders: 35.6% vs. 24.4% have a low level. The gap is even wider for those also reporting work limitations: 40.6% vs. 24.9%.

Table 9. People with MSDs and work limitations by labour market status

Labour market status	MSDs	No MSD	MSD + work limitations	All others
Employed	58.1	70.3	43.2	70.3
Unemployed	6.6	7.1	8.4	6.9
Inactive	35.3	22.7	48.4	22.8
Total %	100.0	100.0	100.0	100.0
N (sample)	41 000	264 000	19 000	286 000

Eurostat – LFS2011 – special module on disability – EU28 – 20-64 years

The employment rate is lower among people with MSDs than among people with no such disorders (12.2 percentage points lower); among those with MSD experiencing work limitations the gap widens to 27.1 percentage points. By contrast, the inactivity rate is always much higher.

Table 10. Active People with MSDs and work limitations by employment status

Employment status	MSDs	No MSD	MSD + work limitations	All others
Full-time job	78.0	82.4	69.1	82.4
Part-time job	22.0	17.6	30.9	17.6
Total %	100.0	100.0	100.0	100.0
N (sample)	24 000	186 000	8 000	202 000

Eurostat – LFS2011 – special module on disability – EU28 – 20-64 years

A large majority of the active people with MSD experiencing work limitations are employees (81%), 17% are self-employed (with or without employees) and 2% are family workers. The employment status of all other active people is not much different (figures not shown in tables).

Active people with MSDs are a bit more often in part-time jobs than people without such disorders (22.0% vs 17.6% = 4.4 percentage points difference). The difference is greater for those with work limitations (30.9% vs 17.6% = 13.3 pp).

Of the active people with MSD 15% attribute part-time work to disability or illness compared to 3% of the people with no such disorders. Among those with work limitations the figures are 28% compared to 3%.

People with MSDs less often hold a temporary job (10.5% vs 13.1%); those with work limitations have a pattern similar to others (12.6% vs 12.8%).

A very high number of people with MSD experiencing work limitations are limited in the type of work (94%) or the amount of work (64%) they can perform. About a third (35%) have problems getting to and from work. However it should be kept in mind that some of these limitations may be caused by other long-standing health conditions or diseases.

A third (33%) of the people with MSD experiencing work limitations report a need for special working arrangements, 18% need workplace adaptations and 15% need personal assistance with the provision that some of these needs may be related to other long-standing health conditions or diseases.

3. Metabolic disorders

Two LFS items *stomach, liver, kidney or digestive problems* (8) and *diabetes* (9) can be combined into the category “metabolic disorders”, each contributing for about half to the new category.

3.7% of all the respondents report “metabolic disorders” as first or second most important long-standing health condition or disease; for 50% this is their sole health problem. Less than half of these respondents - 1.4% of all the respondents – report limitations in work. About 30% have a metabolic disorder as sole health problem.

The following figures relate to all the people with metabolic disorders, whether they have other health problems or not.

Table 11. People with metabolic disorders and work limitations by sex

Sex	Metabolic disorders	No metabolic disorders	metabolic disorders + work limitations	All others
Male	52.3	49.7	48.5	49.8
Female	47.7	50.3	51.5	50.2
Total %	100.0	100.0	100.0	100.0
N (sample)	11 000	294 000	4 000	301 000

Eurostat – LFS2011 – special module on disability – EU28 – 20-64 years

A little over half of the respondents with metabolic disorders are male, compared to half of those without such disorders. Among the people with metabolic disorders experiencing limitations in work 51.5% are female compared to 50%.

Table 12. People with metabolic disorders and work limitations by age

Age	Metabolic disorders	No metabolic disorders	Metabolic disorders + work limitations	All others
20-29	7.5	21.2	4.7	20.9
30-39	12.5	23.3	9.1	23.1
40-49	21.3	24.5	18.2	24.5
50-59	35.8	21.5	40.5	21.7
60-65	22.9	9.6	27.4	9.8
Total %	100.0	100.0	100.0	100.0
N (sample)	11 000	294 000	4 000	301 000

Eurostat – LFS2011 – special module on disability – EU28 – 20-64 years

Table 12 clearly indicates that metabolic disorders occur in every age group but are more common among people aged 50 and over. The same pattern shows in the subgroup of people with metabolic disorders experiencing work limitations.

Table 13. People with metabolic disorders and work limitations by education attainment level

Education attainment	Metabolic disorders	No metabolic disorders	Metabolic disorders + work limitations	All others
Low	36.0	25.5	42.7	25.7
Medium	44.9	48.7	45.0	48.6
High	19.1	25.7	12.3	25.7
Total %	100.0	100.0	100.0	100
N (sample)	11 000	294 000	4 000	301 000

Eurostat – LFS2011 – special module on disability – EU28 – 20-64 years

The education attainment level of all the people with metabolic disorders is much lower than that of people with no such disorders (36% vs. 25.5% have a low level). The gap is even wider for those also reporting work limitations (42.7% vs. 25.7%).

Table 14. People with metabolic disorders and work limitations by labour market status

Labour market status	Metabolic disorders	No metabolic disorders	Metabolic disorders + work limitations	All others
Employed	52.8	69.2	32.1	69.1
Unemployed	7.0	7.0	6.9	7.0
Inactive	40.2	23.8	60.9	23.9
Total %	100.0	100.0	100.0	100.0
N (sample)	11 000	294 000	4 000	301 000

Eurostat – LFS2011 – special module on disability – EU28 – 20-64 years

The employment rate is much lower among people with metabolic disorders than among people with no such disorders (16.4 percentage points lower); among those with metabolic disorders and work limitations it is even 37 percentage points lower. By contrast, the inactivity rate is always much higher.

Table 15. Active People with metabolic disorders and work limitations by employment status

Employment status	Metabolic disorders	No metabolic disorders	Metabolic disorders + work limitations	All others
Full-time job	80	82	67	82
Part-time job	20	18	33	18
Total %	100.0	100.0	100.0	100.0
N (sample)	6 000	203 000	1 300	208 000

Eurostat – LFS2011 – special module on disability – EU28 – 20-64 years

A large majority of active people with metabolic disorders are employees (81.3%), 17.4% are self-employed (with or without employees) and 1.3% are family workers. The employment status of active people without metabolic disorders is very similar. However among those experiencing work limitations, there are less employees (74% vs 84%), more self-employed (23% vs 14%) and more family workers (3% vs 1%) than among all others (Figures not shown in tables).

Active people with metabolic disorders are somewhat more often in part-time jobs than people with no such disorders (20% - 18% = 2 percentage points difference) but the difference is greater among those experiencing work limitations (33% - 18% = 15 pp). Of the active people with metabolic disorders 14% attribute part-time work to disability or illness compared to 4% of the people with no

such disorders. Among those with work limitations, the figures are 33% compared to 4%. People with metabolic disorders less often hold temporary jobs than those without (11% vs 13%) but those experiencing work limitations more often hold temporary jobs than all others (14% vs 13%).

A very high number of the people with metabolic disorders experiencing work limitations are limited in the type of work (91%) or the amount of work (76%) they can perform; 43% have problems getting to and from work. However it should be kept in mind that some of these limitations may be caused by other long-standing health conditions or diseases.

Nearly four out of ten (38%) people with metabolic disorders experiencing work limitations report a need for special working arrangements, 19% need personal assistance and 17% need workplace adaptations with the provision that some of these needs may be related to other long-standing health conditions or diseases.

4. Cardiovascular diseases

The LFS item *heart, blood pressure or circulation problems (6)* corresponds to the category CVDs.

A total of 5.1% of all the respondents report CVDs as first or second most important long-standing health condition or disease, for 42% of them it is the sole health problem.

Less than half of these respondents - 2.1% of all the respondents – report limitations in work. One out of four has a CVD as their sole health problem.

The following figures relate to all the people with CVDs, whether they have other health problems or not.

Table 16. People with CVDs and work limitations by sex

Sex	CVDs	No CVDs	CVDs + work limitations	All others
Male	51.9	49.7	51.0	49.8
Female	48.1	50.3	49.0	50.2
Total %	100.0	100.0	100.0	100.0
N (sample)	15 000	290 000	6 000	299 000

Eurostat – LFS2011 – special module on disability – EU28 – 20-64 years

A total of 51.9% of all the respondents with CVDs are male, compared to 49.7% of all the respondents without. Among people with CVDs experiencing limitations in work 51% are male compared to 49.8% of all others.

Table 17. People with CVDs and work limitations by age

Age	CVDs	No CVDs	CVDs + work limitations	All others
20-29	2.6	21.7	1.9	21.1
30-39	6.6	23.7	4.6	23.3
40-49	17.8	24.7	13.9	24.6
50-59	42.5	20.9	44.3	21.5
60-65	30.5	9.0	35.2	9.5
Total %	100.0	100.0	100.0	100.0
N (sample)	15 000	290 000	6 000	299 000

Eurostat – LFS2011 – special module on disability – EU28 – 20-64 years

Nearly three quarters (73%) of all the people with CVDs are 50 or older; for those experiencing work limitations it is even more (81.5%).

Table 18. People with CVDs and work limitations by education attainment level

Education attainment	CVDs	No CVDs	CVDs + work limitations	All others
Low	33.3	25.5	39.0	25.6
Medium	48.9	48.6	50.3	48.6
High	17.8	25.9	10.7	25.8
Total %	100.0	100.0	100.0	100.0
N (sample)	15 000	290 000	6 000	299 000

Eurostat – LFS2011 – special module on disability – EU28 – 20-64 years

The education attainment level of people with CVDs is lower than that of people with no such diseases (33.3% low vs. 25.5% have a low level). The gap is even wider for those also reporting work limitations (39.0% vs. 25.6%).

Table 19. People with CVDs and work limitations by labour market status

Labour market status	CVDs	No CVDs	CVDs + work limitations	All others
Employed	49.8	69.7	29.1	69.5
Unemployed	5.1	7.1	5.4	7.0
Inactive	45.1	23.2	65.5	23.5
Total %	100.0	100.0	100.0	100.0
N (sample)	15 000	290 000	6 000	299 000

Eurostat – LFS2011 – special module on disability – EU28 – 20-64 years

The employment rate is much lower among people with CVDs than among people with no such diseases (19.9 percentage points lower); among those with CVDs experiencing work limitations the gap widens to 40.4 percentage points. The unemployment rate is somewhat lower but the inactivity rate is much higher.

Table 20. Active people with CVDs and work limitations by employment status

Employment status	CVDs	No CVDs	CVDs + work limitations	All others
Full-time job	81.1	81.9	68.1	82.0
Part-time job	18.9	18.1	31.9	18.0
Total %	100.0	100.0	100.0	100.0
N (sample)	8 000	201 000	2 000	207 000

Eurostat – LFS2011 – special module on disability – EU28 – 20-64 years

A majority of the active people with CVDs experiencing work limitations are employees (72%), 23% are self-employed (with or without employees) and 5% are family workers. The employment status of active people without CVDs and experiencing work limitations differs significantly (84%, 14% and 1% respectively).

Active people with CVDs are slightly more often in part-time jobs than people without such diseases (18.9% - 18.1% = 0.8 percentage points difference). However, the difference is much greater among those also experiencing work limitations (31.9% - 18.0% = 13.9 pp).

Of the active people with CVDs 16% attribute part-time work to disability or illness compared to 4% of the people with no such disease. Among those with work limitations, the figures are 35% compared to 4%.

People with CVDs less often hold a temporary job (9.4% vs 12.9%) but for those with work limitations there is no difference (12.7% vs 12.8%).

A very high number of the people with CVDs experiencing work limitations are limited in the type of work (94%) or the amount of work (76%) they can perform and 42% have problems getting to and from work. However it should be kept in mind that some of these limitations may be caused by other long-standing health conditions or diseases.

More than one third (38%) of the people with CVDs experiencing work limitations report a need for special working arrangements, 18% need personal assistance and 15% need workplace adaptations, with the provision that some of these needs may be related to other long-standing health conditions or diseases.

5. Respiratory diseases

The LFS item *Chest or breathing problems, including asthma and bronchitis (7) corresponds* to the category “respiratory diseases”.

A total of 2.4% of all the respondents report respiratory diseases as first or second most important long-standing health condition or disease, for 44% of them this is their sole health problem. Less than half of these respondents – 0.9% of all respondents – report limitations in work. About one out of four have a respiratory disease as their sole health problem.

The following figures relate to all the people with respiratory diseases, whether they have other health problems or not.

The absolute number of people with respiratory diseases also reporting work limitations is too small in the sample (some 2 600) to perform a valid statistical analysis. Therefore we provide figures on the total of 2.4% people with respiratory diseases; comments and figures for the subgroup with work limitations are placed between brackets.

Half of the people with respiratory diseases are men, half are women; (the same goes for the subgroup).

Table 21. People with respiratory diseases by age

Age	Respiratory diseases	No respiratory diseases
20-29	16.4	20.8
30-39	18.7	23.0
40-49	21.7	24.4
50-59	27.2	21.9
60-65	15.9	9.9
Total %	100.0	100.0
N (sample)	7 000	298 000

Eurostat – LFS2011 – special module on disability – EU28 – 20-64 years

Respiratory diseases are more frequent in the age categories above 50 but are also present in younger age categories. (The pattern is the same for the subgroup).

Table 22. People with respiratory diseases by education attainment level

Education attainment	Respiratory diseases	No respiratory diseases
Low	32.4	25.8
Medium	45.1	48.7
High	22.5	25.6
Total %	100.0	100.0
N (sample)	7 000	297 000

Eurostat – LFS2011 – special module on disability – EU28 – 20-64 years

The education attainment level of all the people with respiratory diseases is lower than that of people with no such disease (32.4% vs. 25.8% have a low level). (The gap is twice as large for the subgroup).

Table 23. People with respiratory diseases by labour market status

Labour market status	Respiratory diseases	No respiratory diseases
Employed	58.4	68.9
Unemployed	8.1	7.0
Inactive	33.5	24.1
Total %	100.0	100.0
N (sample)	7 000	298 000

Eurostat – LFS2011 – special module on disability – EU28 – 20-64 years

The employment rate is 10 percentage points lower among people with respiratory diseases than among people with no such diseases. (The gap is about 30 percentage points for the subgroup). The unemployment rate is slightly higher but the inactivity rate is much higher. (The same goes for the subgroup).

A majority of the people with respiratory diseases are employees (86%), 13% are self-employed (with or without employees) and 1% are family workers. This is not different from the pattern for people with no respiratory diseases.

Active people with respiratory diseases are more often in part-time jobs than people with no such diseases (22% vs 18%) (the difference is twice as large for the subgroup).

Of all the active people with respiratory diseases 9% attribute part-time work to disability or illness compared to 4% of the people with no such disease (the difference is twice as large for the subgroup). People with respiratory diseases also more often hold a temporary job than people with no such diseases (15% vs 13%) (the same difference is found for the subgroup).

The following findings should be interpreted with caution because the absolute numbers of respondents were very small.

A very high number of people with RD experiencing work limitations are limited in the type of work (93%) or the amount of work (67%) they can perform and 39% have problems getting to and from work. However it should be kept in mind that some of these limitations may be caused by other long-standing health conditions or diseases.

One third (33%) of the people with RD experiencing work limitations report a need for special working arrangements, 18% need personal assistance and 16% need workplace adaptations, with the provision that some of these needs may be related to other long-standing health conditions or diseases.

6. Neurological diseases

The category "neurological diseases" is limited to the LFS item *severe headaches such as migraine* (11).

A total of 1.7% of all the respondents report neurological diseases as first or second most important long-standing health condition or disease, for 33% of them this is their sole health problem.

Less than half of these respondents – 0.5% of all respondents – report limitations in work. For 18% this is their sole health problem.

The following figures relate to all the people with neurological diseases whether they have other health problems or not.

The absolute number of people with neurological diseases reporting limitations in work is too small in the sample (some 1 600) to perform a valid statistical analysis. Therefore we only provide figures for the total of 1.7% people with neurological diseases; comments and figures for the subgroup with work limitations are placed between brackets.

Among the people with neurological diseases 27% are male and 73% female (the same goes for the subgroup).

The age distribution is identical to that of people without neurological diseases.

The level of education attainment (29% low, 45% medium, and 26% high) is about the same as for people without neurological diseases.

The employment rate (66%) is only slightly under that of all the other people (69%).

There are nearly 4% more employees among the people with neurological diseases than among all the others.

People with neurological diseases are more often in part-time jobs than others (25% vs 18%) but they have as many permanent work contracts.

Although the number of respondents with neurological diseases reporting work limitations is very small, we provide the figures for the work limitations and the needs.

A high number of people with neurological diseases and work limitations are limited in the type of work (84%) or the amount of work (67%) they can perform and 35% have problems getting to and from work. However it should be kept in mind that some of these limitations may be caused by other long-standing health conditions or diseases.

Nearly one third (30%) of the people with neurological diseases and work limitations report a need for special working arrangements, 14% need personal assistance and 13% need workplace adaptations, with the provision that some of these needs may be related to other long-standing health conditions or diseases.

To conclude we present a summary table of employment rates clearly showing the relationship between health conditions or diseases and labour market participation.

Table 24. Employment rates of people with longstanding health conditions or diseases

Type of health condition or disease	Employment rate (%)	
	All respondents	Respondents with work limitations
All without health condition or disease	73	-
All with health condition or disease**	56	39
Neurological (severe headaches)	66	*
Musculoskeletal disease	58	43
Respiratory disease	58	*
Metabolic disorders	53	32
Cardiovascular	50	29
Mental health issues	41	26

Eurostat – LFS2011 – special module on disability – EU28 – 20-64 years
**not available / **including diseases not mentioned in the table.*

Annex 7: International frameworks

United Nations

UN Convention on the Rights of Persons with Disabilities (UNCRPD)[76]. The treaty on the rights of persons with disabilities was adopted in 2006. Article 27 of the Convention recognises the right of persons with disabilities to work on an equal basis with others. The European Union ratified the Convention on December 23, 2010; by 2016, 26 EU Member States have ratified the Convention. According to the UNCRPD, persons with disabilities include people with "long-term physical, mental, intellectual or sensory impairments which in interaction with various barriers may hinder their full and effective participation in society on an equal basis with others". Many of the above-mentioned impairments can be caused by NCDs and, therefore, it may be concluded that the definition of persons with disabilities can also include persons with chronic diseases.

UN Political Declaration on Prevention and Control of Non-communicable Diseases[77]***.*** On September 19, 2011, the UN General Assembly adopted the political declaration on prevention and control of NCDs, which acknowledged that chronic diseases undermine social and economic development and challenge the achievement of internationally agreed development goals in the 21st century. It also recognised that effective disease prevention and control require mainstreaming of health policies in various areas including labour, employment and industry. The EU supported the adoption of the Declaration.

Transforming our world: the 2030 Agenda for Sustainable Development[78] is a resolution adopted by the UN General Assembly on 25 September 2015. The document sets 17 Sustainable Development Goals with 169 targets to be achieved by 2030. The agenda includes the following targets:

- Empower and promote the social, economic and political inclusion of all, irrespective of age, sex, disability, etc.
- Achieve full and productive employment and decent work for all, including for persons with disabilities, and equal pay for work of equal value.

---

[76] http://www.un.org/disabilities/documents/convention/convoptprot-e.pdf
[77] http://www.who.int/nmh/events/un_ncd_summit2011/political_declaration_en.pdf
[78] http://www.un.org/ga/search/view_doc.asp?symbol=A/RES/70/1&Lang=E

World Health Organisation

Gaining Health: European Strategy for the Prevention and Control of Noncommunicable Diseases[79]**.** The strategy was written in 2006 and is mainly focused on reducing the burden of NCDs through prevention and control measures. However, within the framework of action proposed by the strategy, there is a call to enable all persons to reach their full potential through advocacy for health in all policies, better educational and employment opportunities for people with mental health issues, removal of barriers to employment, such as unfair discrimination against people with disabilities and long-term illness, and support for older people in staying physically active and independent.

Action Plan for implementation of the European Strategy for the Prevention and Control of NCDs 2012–2016[80]. The document, published in 2012, derived from the European Strategy for the Prevention and Control of Noncommunicable Diseases and identified specific action areas and deliverables to which Member States of the European Region, WHO and partners can commit. The action plan includes the development of a policy framework for promoting healthy workplaces, reducing inequality in health and endorsing disease prevention programmes. A progress report on the Action plan finds that an increasing number of Member States in the European Region has included NCDs in their social and economic development plans. Furthermore, the "health in all policies" principle is being activated in a variety of settings to address NCDs across various sectors including education and employment. [81]

WHO Global Disability Action Plan 2014-2021[82] was endorsed in 2014 and called for removing barriers in accessing various services and programmes, including those related to employment and education, rehabilitation, assistive devices and support services, and community-based rehabilitation.

WHO Mental Health Action Plan 2013-2020[83] was adopted by the 66th World Health Assembly in May 2013. The action plan is based on a multi-sectoral and empowerment approach, and aims to achieve equity through universal health coverage. One of the major objectives of the document is to provide comprehensive, integrated mental health and social care services in community-based

[79] Available at http://www.euro.who.int/__data/assets/pdf_file/0008/76526/E89306.pdf
[80] Available at http://www.euro.who.int/__data/assets/pdf_file/0019/170155/e96638.pdf?ua=1
[81] Available at http://www.euro.who.int/__data/assets/pdf_file/0004/235975/Prevention-and-control-of-noncommunicable-diseases-in-the-European-Region-A-progress-report-Eng.pdf?ua=1
[82] Available at http://apps.who.int/iris/bitstream/10665/199544/1/9789241509619_eng.pdf?ua=1
[83] Available at http://apps.who.int/iris/bitstream/10665/89966/1/9789241506021_eng.pdf?ua=1

settings. It also stresses the importance of strengthening information systems, evidence and research.

- To recognise people with mental health conditions as a vulnerable group requiring "prioritized attention and engagement within development and poverty-reduction strategies, for example, in education, employment and livelihood programmes, and the human rights agenda" (WHO, 2013: 13).
- To ensure that community-based service delivery for mental health encompasses a "recovery-based approach that puts the emphasis on supporting individuals with mental health conditions and psychosocial disabilities to achieve their own aspirations and goals. The core service requirements include: listening and responding to individuals' understanding of their condition and what helps them to recover; working with people as equal partners in their care; offering choice of treatment and therapies, and in terms of who provides care; and the use of peer workers and supports, who provide each other with encouragement and a sense of belonging, in addition to their expertise. In addition, a multisectoral approach is required whereby services support individuals, at different stages of the life course and, as appropriate, facilitate their access to human rights such as employment (including return-to-work programmes)", etc. (WHO, 2013: 14).
- "Integrated and responsive care: Integrate and coordinate holistic prevention, promotion, rehabilitation, care and support that aims at meeting both mental and physical health care needs and facilitates the recovery of persons of all ages with mental health conditions within and across general health and social services (including the promotion of the right to employment, housing, and education] through service user-driven treatment and recovery plans and, where appropriate, with the inputs of families and carers" (WHO, 2013: 15).
- "Build the knowledge and skills of general and specialized health workers to deliver evidence-based, culturally appropriate and human rights-oriented mental health and social care services, for children and adolescents, inter alia, by introducing mental health into undergraduate and graduate curricula; and through training and mentoring health workers in the field, particularly in non-specialized settings, in order to identify people with mental health conditions and offer appropriate treatment and support as well as to refer people, as appropriate, to other levels of care"(WHO, 2013: 15).

The European Mental Health Action Plan (2013-2020)[84]. WHO Regional Committee for Europe in 2013 sets out seven objectives. The first objective is to provide equal opportunities for all to realize mental wellbeing throughout their lifespan, particularly for those who are most vulnerable or at risk. One of the outcomes of this objective is increased return to work of people with mental health conditions. Actions proposed by the plan include awareness raising, dissemination of successful workplace interventions, early intervention programmes, etc.

Mental Health Declaration for Europe[85]***.*** In 2005, the Ministers of Health of Member States in the European Region of the WHO endorsed a declaration calling for, among others, to tackle stigma and discrimination, to ensure the protection of human rights and dignity and to implement the necessary legislation in order to empower people at risk or with mental health issues and disabilities to participate fully and equally in society. They committed themselves to enhancing inclusion by increasing public awareness and empowering people at risk, as well as to promoting mental health in employment and to creating incentives for the provision of support at work or the earliest return for those who have recovered from mental health issues.

International Labour Organisation (ILO)

Managing disability in the workplace: ILO code of practice[86]. The code was adopted at the tripartite meeting of experts in Geneva in 2001 with an aim to guide employers to adopt a positive strategy in managing disability related issues in the workplace. The code is based on the principle that employers benefit from the employment of people with disabilities, who can make a significant contribution at their place of employment, in jobs matched to their skills and abilities, if disability-related issues are appropriately managed.

Disability Inclusion Strategy and Action Plan 2014-17[87]. The ILO strategy aims to promote and reflect disability-specific actions in various aspects of its work, including international standards, programming, technical cooperation, research, internal practices and work with constituents.

Organisation for Economic Co-operation and Development (OECD)

---

[84] Available at http://www.euro.who.int/__data/assets/pdf_file/0020/280604/WHO-Europe-Mental-Health-Acion-Plan-2013-2020.pdf?ua=1
[85] Available at http://www.euro.who.int/__data/assets/pdf_file/0008/88595/E85445.pdf?ua=1
[86] Available at http://www.ilo.org/wcmsp5/groups/public/---ed_emp/documents/publication/wcms_103324.pdf
[87] Available at http://www.ilo.org/wcmsp5/groups/public/---ed_emp/---ifp_skills/documents/genericdocument/wcms_370772.pdf

Transforming Disability into ability[88] is a report released in 2003, in which OECD proposes a set of policy recommendations for promoting the employment of persons with disabilities. Based on a review of policies in 20 OECD member countries, the report proposes the following (OECD, 2003):

- Recognition of the status of disability independent of the work and income situation;
- Introducing a "culture of mutual obligation," i.e. ensuring that both employers and persons with disabilities understand their responsibility to put effort towards better work (re-)integration;
- Offering work/benefit packages adapted to the individual needs of persons with disabilities and containing both employment (re-)integration services and disability benefits;
- Introducing new obligations for persons with disabilities to make an effort to participate in employment; making benefits conditional on participation in employment activation activities.
- Activating employers through various measures, be it anti-discrimination legislation, quotas or financial incentives;
- Putting in place early intervention measures to avoid long-term benefit dependence;
- Making cash benefits more flexible so that persons are not penalised when becoming employed;
- Reforming programme administration in a way that disability gatekeepers use a one-stop approach to assist individuals in accessing various active interventions and services;
- Making disability programs more active and more integrated with general labour market interventions.

[88] Available at http://www.keepeek.com/Digital-Asset-Management/oecd/social-issues-migration-health/transforming-disability-into-ability_9789264158245-en#page13
